# Genome-wide meta-analyses of cross substance use disorders in diverse populations

**DOI:** 10.1038/s41380-025-03294-5

**Published:** 2025-10-07

**Authors:** Dongbing Lai, Michael Zhang, Nick Green, Marco Abreu, Tae-Hwi Schwantes-An, Clarissa C. Parker, Shanshan Zhang, Fulai Jin, Anna Sun, Pengyue Zhang, Howard J. Edenberg, Yunlong Liu, Tatiana Foroud

**Affiliations:** 1 Department of Medical and Molecular Genetics, Indiana University School of Medicine, Indianapolis, IN 46202 USA; 2 Department of Psychology and Program in Neuroscience, Middlebury College, Middlebury, VT 05753 USA; 3 Department of Genetics and Genome Sciences, Case Western Reserve University, Cleveland, OH 44106 USA; 4 Computer Science and Artificial Intelligence Lab, Massachusetts Institute of Technology, Cambridge, MA 02139 USA; 5 Department of Biostatistics and Heath Data Science, Indiana University School of Medicine, Indianapolis, IN 46202 USA; 6 Department of Biochemistry and Molecular Biology, Indiana University School of Medicine, Indianapolis, IN 46202 USA

**Keywords:** Addiction, Genetics

## Abstract

Substance use disorders (SUDs, including alcohol, cannabis, opioids, and tobacco) represent significant public health challenges. The estimated heritability of SUDs is ~50% and many individuals experience multiple SUDs concurrently. Studies have demonstrated the existence of genes shared across multiple SUDs, and identifying these SUD-shared genes is critical to developing novel prevention and treatment strategies. Here, we conducted the largest cross SUD meta-analysis to date to identify SUD-shared genes using samples genetically similar to 1000 Genomes Project European (1kg-EUR-like), African (1kg-AFR-like), and American mixed (1kg-AMR-like) populations. We defined variants that had the same direction of effects across different SUDs (i.e., concordant variants) as SUD-shared. In total, we identified 220 loci, including 40 novel loci that were not reported as SUD-associated in previous genome-wide association studies. Through gene-based analyses, gene mapping, and gene prioritization, we identified 785 SUD-shared genes. These genes are highly expressed in the amygdala, cortex, hippocampus, hypothalamus, and thalamus; and are primarily highly expressed in neuronal cells, suggesting that more brain regions may be involved in SUDs than previously reported. Concordant variants explained 56–96% of the SNP-heritability of each SUD in the 1kg-EUR-like sample. Furthermore, the top 10% of individuals in the 1kg-EUR-like and 1kg-AMR-like samples with the highest polygenic scores had odds ratios ranging from 1.95–2.87 to develop SUDs, and these polygenic scores could potentially be used to identify high-risk individuals. Lastly, using a real-world dataset, we identified seven SUD-shared genes targeting drugs that may be repurposed for treating SUDs, particularly in those suffering from comorbid SUDs.

## Introduction

Substance use disorders (SUDs) have devasting consequences on affected individuals, their families, and society. Globally, approximately 5.5% of disability-adjusted life-years are attributable to SUDs [1]. The estimated heritability of each SUD is ~50% [2] and identifying SUD-associated genes will not only help us understand the genetic etiologies of SUDs, but also facilitate the development of novel prevention and treatment strategies.

SUDs share common features such as uncontrolled use of substances and withdrawal/negative affect [3]. Many people suffer from multiple SUDs simultaneously with about one in four individuals diagnosed with SUD having two or more SUDs [4]. Furthermore, in individuals with an SUD diagnosis other than alcohol use disorder, 56.8 to 97.5% had at least one other comorbid SUD diagnosis as well [5]. Twin studies [6–8] and genetic correlation studies [9, 10] have confirmed the existence of genes shared across multiple SUDs. Identification of these SUD-shared genes can elucidate the common genetic pathways to SUDs and importantly, facilitate the development of novel and more effective treatment methods. This is particularly important for individuals with comorbid SUDs as they typically experience more severe mental and physical health challenges and require complex treatment approaches. One way to identify genes shared across multiple SUDs is to perform a univariate analysis by defining SUD cases as having any SUD and defining controls as not having any SUD. However, individual level genotype data is typically required, therefore studies using this method are often limited by small to moderate sample sizes [11–15], making replication difficult. Multivariate methods such as genomic structural equation modeling (genomic SEM) [16] and meta-analysis [17] can utilize summary statistics from large-scale genome-wide association studies (GWAS) and thus are more powerful. Genomic SEM derives a common risk factor by using LD (linkage disequilibrium) score regression [18] and has successfully identified multiple SUD-shared genes [9, 19]. However, model misspecifications and LD pattern mismatches among different studies can lead to results that cannot be replicated by other studies. Additionally, while findings are supposed to reflect genes shared across multiple SUDs, some studies report genes with opposite directions of effects across different SUDs [9]. For example, two variants identified using genomic SEM, rs2424952 and rs28567725, had discordant effects for problematic alcohol use and cannabis use disorder [9]. Although these discordant findings may still reflect shared genetic factors across SUDs, the underlying mechanisms are difficult to interpret. Furthermore, drugs developed targeting these discordant genes may be less effective in individuals with comorbid SUDs.

In this study, we performed the largest cross SUD meta-analysis to date to search for SUD-shared genes in diverse populations. We chose meta-analysis because it does not estimate a common risk factor and thus avoids model misspecifications and LD pattern mismatches. This in turn provides a more powerful approach [20–22]. Since many individuals suffer from comorbid SUDs simultaneously, it is less likely that SUD-shared variants increase the risk for one SUD but decrease the risk for another SUD. Therefore, we considered variants with the same direction of effects in different SUDs (i.e., concordant variants) as SUD-shared for easier interpretation and drug development purposes. Multiple approaches were used to map identified concordant variants to genes, then a series of steps were used to prioritize identified genes. We also performed gene enrichment analyses, estimated the SNP-heritability explained by concordant variants, and calculated genetic correlations. Polygenic scores (PGS) were calculated by using concordant variants and their predictive power for SUD risk was tested using independent datasets. Lastly, we identified drugs targeting prioritized SUD-shared genes and tested whether they could be repurposed to treat SUDs by using a large-scale real-world dataset.

## Materials and methods

### GWAS summary statistics and meta-analysis

GWAS summary statistics of problematic alcohol use (PAU) [23], cannabis use disorder (CUD) [24], opioid use disorder (OUD) [25, 26], tobacco use disorder (TUD) [27], and substance abuse from the FinnGen consortium [28] were included. We selected these four SUD GWAS as they have large sample sizes to ensure sufficient statistical power. Meta-analyses in samples genetically similar to the 1000 Genomes Project (1 kg) [29] European (1kg-EUR-like), African (1kg-AFR-like), and American mixed (1kg-AMR-like) populations were performed. Then three cross-population meta-analyses in 1kg-AFR + EUR-like samples, 1kg-AMR + EUR-like samples, and 1kg-AFR + AMR + EUR-like samples were performed. Table 1 lists the GWAS summary statistics included in this study. The effective sample sizes after correcting for overlapping samples were 1,458,999 (1kg-EUR-like), 240,296 (1kg-AFR-like), 58,370 (1kg-AMR-like), and 1,683,439 (cross-population total). Using the 1kg-EUR-like sample, the genetic correlations among PAU, OUD, CUD and SUD ranged from 0.48 to 0.75 (P-values ≤ 6.46E-26, Supplemental Table 1), indicating shared genetic underpinning across SUDs.

**Table 1:** SUD GWAS included in meta-analyses.

Population	Trait	Diagnosis	# Samples	Cohorts	Authors	PMID
1kg-EUR-like	Problematic alcohol use	DSM-IV, ICD9/10, AUDIT-P	903,147	MVP, FinnGen, UKBB, PGC, QIMR, iPSYCH, YP	Zhou et al. [23]	38062264
	Cannabis use disorder	DSM-IV, ICD9/10	886,025	PGC, deCODE, iPSYCH, MVP, MGB, YP	Levey et al., [24]	37985822
	Opioid use disorder	DSM-IV, ICD9/10	308,733	PGC, MVP	Polimanti et al., [26]; Kember et al., [25]	32099098, 36171425
	Tobacco use disorder	ICD9/10	739,895	UKBB, BioVU, MGB, PMBB, MVP	Toikumo et al. [27]	38632388
	Total		1,458,999			
1kg-AFR-like	Alcohol use disorder	DSM-IV, ICD9/10	122,571	MVP, PGC, YP	Zhou et al. [23]	38062264
	Cannabis use disorder	DSM-IV, ICD9/10	123,208	PGC, MVP, YP	Levey et al. [24]	37985822
	Opioid use disorder		91,026	PGC, MVP	Polimanti et al., [26]; Kember et al., [26]	32099098, 36171425
	Tobacco use disorder	ICD9/10	114,420	PMBB, MVP	Toikumo et al. [27]	38632388
	Total		240,296			
1kg-AMR-like	AUD	ICD9/10	38,962	MVP	Zhou et al. [23]	38062264
	Opioid use disorder	DSM-IV, ICD9/10	34,861	MVP	Kember et al. [25]	36171425
	Total		58,370			
Cross Population total			1,683,439			

the total samples sizes are effective sample sizes, i.e., samples sizes after correcting for sample overlapping. 1kg-AFR-like, 1kg-EUR-like, and 1kg-AMR-like are samples genetically similar to 1000 Genomes Project African, European, and American mixed populations, respectively. For Kember et al. [25], we used the results from the Million Veteran Program.

*PMID* PubMed ID; *MVP* Million Veteran Program; *FinnGen* FinnGen concortium; *UKBB* UK Biobank; *PGC* Psychiatric Genomics Consortium; *QIMR* QIMR Berghofer cohorts; *iPSYCH* The Lundbeck Foundation Initiative for Integrative Psychiatric Research; *YP* Yale-Penn study; *MGB* Mass General Brigham Biobank; *BioVU* Vanderbilt University Medical Center’s biobank; *PMBB* Penn Medicine BioBank.

For each SUD GWAS, variants with strand ambiguities (e.g., those having A/T or C/G alleles) were removed. Then variants with the same rs names, reference alleles, and alternative alleles as those in 1 kg [29] were retained. There were two 1kg-EUR-like and 1kg-AFR-like OUD GWAS [25, 26]; therefore, they were meta-analyzed first to get one GWAS for OUD in each population. All meta-analyses were performed using Metal with sample overlapping correction by using the sample covariance matrix [17]. For analyses in each population, concordant variants were defined as those having the same direction of effects in different SUDs. For cross-population analyses, we further required that concordant variants had the same direction of effects in different populations. Only these concordant variants were considered as SUD-shared and used in the downstream analyses. All results were uploaded to FUMA (Functional mapping and annotation of genetic associations) [30] to identify independent lead variants and significant loci. Independent lead variants were those genome-wide significant (GWS) variants with the smallest P-values and LD r^2^ <0.1 with other GWS variants. A significant locus was defined as a chromosome region surrounding an independent lead variant bordered by variants having LD r^2^ > 0.6 with the independent lead variant. If the distance between two loci was <250 kb, they were merged. European, African, and American mixed samples from 1 kg [29] were used to determine LD for 1kg-EUR-like, 1kg-AFR-like, and 1kg-AMR-like samples, respectively. FUMA [30] was also used to make Manhattan plots. LocusZoom plots were generated by using LocusZoom.js [31]. Allele frequencies of variants were obtained from 1 kg [29] by using ANNOVAR [32].

### Ethics approval and consent to participate

All methods were performed in accordance with the relevant guidelines and regulations. This study was approved by the Indiana University Institutional Review Borad (protocol number: 1610935245). Informed consent was obtained from all participants at each study.

### Gene-based analysis

MAGMA [33] was used to perform gene-based analyses. Since SUDs are psychiatric disorders, only genes (N = 33,761) having median transcripts per kilobase million (TPM) > 0 in at least one of 13 brain tissues from GTEx [34] were included in gene-based analyses. Within each population, the top χ^2^ statistic option in MAGMA [33] was used to perform the gene-based analysis. Cross-population gene-based analyses were performed by using the meta-analysis method in MAGMA [33] with gene-based analysis results from each population as the input files. Genes presented in the analysis results of only one population were excluded.

### Mapping significant variants to genes

Three strategies were used to map significant variants to genes: 1) Positional mapping using FUMA [30], i.e., whether a significant variant was located within a gene ( ± 10 kb from transcription start and end sites). Gene annotations were obtained from ENSEMBL gene v102 [35] by using ANNOVAR [32]. 2) eQTL mapping using FUMA [30], i.e., whether a significant variant was an eQTL of a gene (cis-eQTL only). eQTL datasets from PsychENCODE [36] were used. 3): Chromatin interaction mapping using FUMA, i.e., whether a variant contacted a gene by chromosomal looping (within 2 Mb). Similar to our gene-based analysis, we limited our analysis to genes that are known to be expressed in at least one brain tissue from GTEx [34]. Genes mapped by any of these strategies were included in gene prioritization.

### Gene prioritization

Some mapped genes may be physically close to SUD-shared genes but may not be biologically relevant to SUDs. Therefore, we prioritized mapped genes using a series of steps. First, we checked whether mapped genes were in SUD-related pathways (alcoholism, amphetamine addiction, cocaine addiction, morphine addiction, nicotine addiction, dopaminergic synapse, GABAergic synapse, glutamatergic synapse, and MAPK signaling) defined by the Kyoto Encyclopedia of Genes and Genomics (KEGG: https://www.genome.jp/kegg/). Given the fact that these pathways were derived from animal studies and different genes with similar functions may cause SUDs in humans, we also checked whether any of the prioritized genes belonged to the same gene families as those in KEGG SUD pathways, or if they directly interacted with genes in those pathways. Gene-gene interactions were obtained from the STRING database (https://stringdb.org/) [37]. Genes with high confidence interactions (i.e., a confidence score ≥0.7 or obtained from high-throughput laboratory experiments and previous knowledge in databases) were retained. Finally, we queried the GWAS catalog [38] and retained genes that were associated with psychiatric traits, brain measurements, or brain functions. Genes identified by any of these steps were prioritized.

### Brain dissection and cell type enrichment analyses

Four enrichment analyses were performed to identify: 1) in which brain dissections prioritized genes were highly expressed, 2) in which brain dissections prioritized genes were expressed at low levels, 3) in which brain cell types prioritized genes were highly expressed, and 4) in which brain cell types prioritized genes were expressed at low levels. We used single-cell RNA sequencing data from 105 dissections and 461 cell types generated by the BRAIN Initiative cell census network [39]; and to be consistent with the original publications, we used the same naming system. For the first enrichment analysis, the mean and standard error (SE) of RNA expression across all dissections were calculated for each gene; then we tested whether the expression of that gene in a particular dissection was significantly higher than the mean expression (> mean + 1.645*SE; α = 0.05). Each gene in each dissection was labeled as either highly or not highly expressed. Then, for each dissection, we tested whether the frequency of highly expressed prioritized genes was significantly greater than the frequency of all other genes using the Fisher’s exact test. Benjamini-Hochberg false discovery rate [40] was used to correct for multiple testing. The same procedure was performed in the second enrichment analysis except we tested whether the gene expression was significantly lower than the mean expression (<mean - 1.645*SE; α = 0.05). The other two enrichment analyses used the same procedure but tested cell types.

### SNP-heritability estimation and calculating SNP-heritability explained by concordant variants for each SUD

Complex Traits Genetics Virtual Lab (CTG-VL https://vl.genoma.io/data) was used to estimate SNP-heritability using LD score regression [18]. SNP-heritability explained by concordant variants was estimated by using the LD score regression model [18, 41] implemented in LDAK (v5.2) [42, 43] on the observed scale. In order to most accurately estimate LD patterns, we limited this analysis to the 1kg-EUR-like sample due to lack of access to individual level data for the 1kg-AFR-like and 1kg-AMR-like samples. To estimate SNP-heritability in 1kg-EUR-like sample, we made a European reference panel by including Finnish samples from 1 kg [29] following the instruction of LDAK [42, 43] as data from the FinnGen consortium were included in our analyses. SNP-heritability was estimated by 1) using all available variants in each SUD GWAS, and 2) limited to concordant variants only. The estimate in 2) divided by the estimate in 1) was the SNP-heritability explained by concordant variants for each SUD.

### Calculating genetic correlations

CTG-VL was also used to perform genetic correlation analyses using LD score regression [18]. To accomplish this, every individual SUD GWAS was uploaded to CTG-VL to calculate genetic correlations between the different SUDs. All variants from each SUD GWAS were included. We also calculated genetic correlations between our cross SUD meta-analysis (concordant variants only) and other publicly available GWAS, which are available at CGT-VL. Significant correlations were determined by Bonferroni correction. Genetic correlation analyses were limited to the 1kg-EUR-like sample for the same reason as the SNP-heritability estimations.

### Polygenic score analyses

Samples from the All of Us research program (AOU) [36] and the Indiana Biobank (IB) [44] were used as the target datasets to perform PGS analyses. Genetically determined 1kg-EUR-like, 1kg-AFR-like, and 1kg-AMR-like samples from AOU version 8 dataset were used; variants with a population-specific allele frequency >1% or a population-specific allele count >100 in any population were included. In the AOU dataset, SUD status was obtained from participants’ electronic health records (EHR), then refined and validated by the AOU Data and Research center. IB data processing and QC were performed as described previously [44, 45]. The IB dataset did not have a sufficient sample size for the 1kg-AMR-like sample, therefore PGS analyses were performed only in 1kg-AFR-like and 1kg-EUR-like samples. SUD status was also obtained from EHRs and determined based on ICD9/10 codes. In both AOU and IB datasets, SUD cases were defined as those individuals having any SUD (i.e., not limited to AUD, CUD, OUD, and TUD) and controls were defined as everyone else. For any related samples (as determined by identity by descent), we kept only one individual from each family by first selecting those having SUDs and/or higher call rates. In cases where the related individuals were all controls or had similar call rates, then we randomly selected one individual from each family.

For the 1kg-EUR-like sample, we used the 1kg-EUR-like meta-analysis results as the discovery dataset and European samples from 1 kg [29] as the reference panel to estimate posterior effect sizes of concordant variants using PRS-CS [46]. For the 1kg-AFR-like and 1kg-AMR-like samples, in addition to the meta-analyses results from 1kg-AFR-like and 1kg-AMR-like, respectively, 1kg-EUR-like results were also used as the discovery dataset to increase the statistical power. However, since variants with discordant effects across different populations decrease the statistical power, we only kept 1kg-EUR-like variants with concordant effects in the1kg-AFR-like or 1kg-AMR-like samples. Combined posterior effect sizes across different populations were estimated by using PRS-CSx (i.e., meta-analysis results from PRS-CSx) [47]. African (for 1kg-AFR-like) and American Mixed (for 1kg-AMR-like) samples from 1 kg [29] were used as the reference panels. In the AOU dataset, PGS were calculated as the sum of the numbers of alleles multiplied by the posterior effect sizes. If a genotype was not successfully sequenced, then it was imputed as mean allele counts based on the allele frequencies via PLINK [48, 49]. In the IB dataset, PGS were calculated as the sum of imputation dosages multiplied by the posterior effect sizes.

To identify high-risk individuals based on their PGS, we compared the top 10% of individuals with the highest PGS to the remaining 90%. We used the top 10% as this approximately represents the prevalence of SUDs in the AOU 1kg-EUR-like and 1kg-AMR-like populations. Additionally, since the prevalence of SUDs differs between males and females, sex-stratified analyses were performed. Logistic regression was used with sex (not used in sex-stratified analyses), age, and the first 10 principal components of genetic ancestries as covariates. For SUD cases, age was defined as the age at the first diagnosis; for controls, age was defined as the age at the last interview. An array indicator was also included as a covariate in the analyses of IB as IB data were genotyped on two arrays. We also meta-analyzed the AOU and IB results in 1kg-EUR-like and 1kg-AFR-like samples weighted by the inverse of standard error using Metal [17].

### Drug repurposing

The Drug Gene Interaction Database (DGIdb, v4.2.0, https://www.dgidb.org/) [50] was queried to identify drugs targeting our list of prioritized genes. Only drugs approved by the Food and Drug Administration (FDA) were included. The Anatomical Therapeutic Chemical (ATC) codes of drugs were obtained from the KEGG: https://www.genome.jp/kegg/drug/). Repurposable drugs were selected based on four criteria: 1) have an ATC code N, 2) are not currently approved to treat any SUD, 3) are shown to be related to SUD treatment through a literature search [51–58], and 4) have comparators (i.e., drugs with the same ATC level 4 codes as drugs we identified but do not target any gene we identified). Since the identified drugs belong to four ATC level 4 classes (N03AX, N05AX, N06AA, and N06BA), four cohorts corresponding to the four ATC classes using the active comparator design were derived. Data were obtained from Optum’s de-identified Clinformatics® Data Mart Database, which is a longitudinal database derived from medical claims and pharmacy claims data of a large national insurer cohort (https://www.optum.com/business/life-sciences/commercial-analytics/managed-markets.html). In each cohort, individuals with pharmacy claims belonging to the corresponding ATC class were identified with the year of drug initiation between 2008 to 2021. All individuals were 21 years or older. The clock-0 was defined as the first exposure date of repurposable drug or comparator and individuals with >365 days of enrollment prior to the clock-0 were included. Individuals concurrently initiated on both repurposable drugs and comparators on clock-0 were excluded.

In the active comparator design, the “repurposable drugs” were those drugs identified and the comparators were the remaining drugs in the corresponding repurposable drugs’ ATC level 4 classes. The outcome was time from clock-0 to diagnosis of SUDs. Individuals without an outcome were censored on the earliest date of drug discontinuation (i.e., without exposure for >60 days), enrollment discontinuation, or concurrent of initial of repurposable drug and comparator. The R package comorbidity was used to define the outcome and comorbidities. Cox proportional hazard models were used to investigate the association between repurposable drugs and the outcome. Cox proportional models were adjusted for age at clock-0, sex, race, and comorbidities, and stratified by the year of drug initiation.

## Results

### Identification of SUD-shared loci

**Fig. 1:**
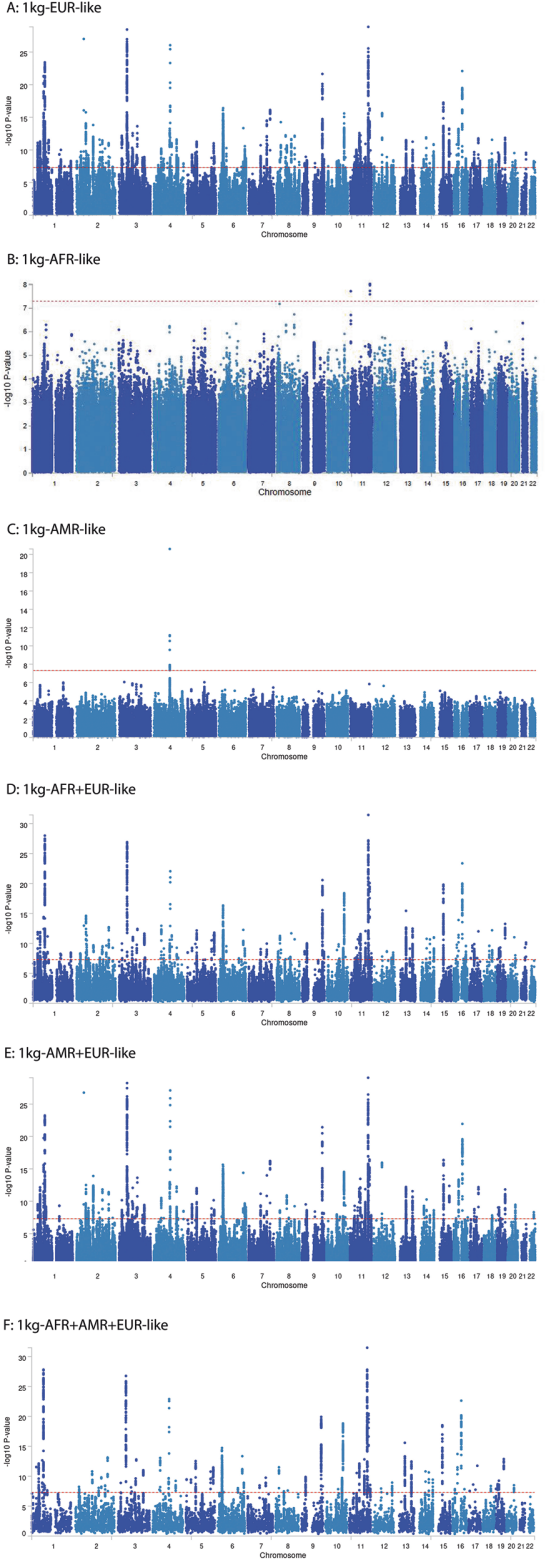
Manhattan plots. Only concordant variants are shown. **A**: 1kg-EUR-like; **B**: 1kg-AFR-like; **C**: 1kg-AMR-like; **D**: meta-analysis of 1kg-AFR-like and 1kg-EUR-like; **E**: meta-analysis of 1kg-EUR-like and 1kg-AMR-like; **F**: meta-analysis of 1kg-AFR-like, 1kg-EUR-like, and 1kg-AMR-like.

We identified 428 independent lead variants (184 loci) in the 1kg-EUR-like sample, two independent lead variants (2 loci) in the 1kg-AFR-like sample, and three independent lead variants (1 locus) in the 1kg-AMR-like sample (Supplemental Table 2–4). In the 1kg-EUR-like sample, there were 8,220 GWS variants, and among 184 loci identified, 155 loci had ≥2 GWS variants. Locus 1 in the 1kg-AFR-like sample was an intergenic variant without any LD support; therefore, it was likely a false positive and was excluded from further analysis. The locus identified in the 1kg-AMR-like sample included alcohol metabolism genes, which are associated with AUD [59]. The 1kg-AMR-like sample only had AUD and OUD GWAS, and it is likely that this finding was driven by AUD. However, we included this locus in subsequent analyses as we could not rule out its role in other SUDs. In the cross-population meta-analysis, we identified 226 independent lead variants (135 loci) in the 1kg-AFR + EUR-like samples, 243 independent lead variants (113 loci) in the 1kg-EUR + AMR-like samples, and 144 independent lead variants (82 loci) in the 1kg-AFR + AMR + EUR-like samples (Supplemental Tables 5–7). Manhattan plots of each meta-analysis are displayed in Fig. 1 (only showing concordant variants). After merging loci identified in all meta-analyses if they had inter-loci distances <250 kb, there were 220 loci in total with 40 novel loci not previously reported as associated with any SUD related phenotype in the GWAS catalog [38] (Table 2). Among those 40 novel loci, 32 were associated with psychiatric traits such as insomnia, depression, and educational attainment, but the other eight were not associated with psychiatric traits in the GWAS catalog [38]. Thus, further studies are needed to confirm and understand these findings. LocusZoom plots of these 220 loci from the various meta-analyses are displayed in Supplemental Fig. 1 (only showing concordant variants). Notably, locus 158 was significant in both the 1kg-EUR-like and 1kg-AFR-like samples, but no other locus was significant in more than one population. Additionally, 152 loci were identified by multiple meta-analyses (Table 2), and many of these had different independent lead variants in different meta-analyses (Supplemental Fig. 1).

**Table 2:** Genome-wide significant loci and their lead variants.

Locus	Chr	Locus start	Locus end	Lead variant	BP	Effect Allele	Non-Effect Allele	Z	EUR.P-value	AFR.P-value	AMR.P-value	AFR.EUR.P-value	AMR.EUR.P-value	AFR.AMR.EUR.P.value
1	1	28,634,681	29,169,593	rs2236855	29,161,999	A	C	7.09	**1.17E-09**	6.20E-04	0.60	***1.32E-12***	**2.32E-09**	**2.89E-12**
2	1	32,168,381	32,221,913	rs3766823	32,197,257	A	G	5.66	2.62E-07	NA	7.56E-04	NA	***1.49E-08***	NA
3	1	43,760,236	44,295,047	rs951740	44,011,737	A	G	7.18	**8.04E-12**	NA	8.45E-03	NA	***7.08E-13***	NA
**4**	1	45,168,480	45,751,415	rs35809187	45,412,276	A	C	5.68	**3.54E-08**	NA	0.09	NA	***1.32E-08***	NA
5	1	46,041,100	46,598,273	rs61784801	46,179,022	A	C	−5.98	**3.49E-09**	0.40	0.16	1.79E-07	***2.30E-09***	7.27E-08
6	1	49,463,925	50,591,851	rs241501	49,765,438	T	G	5.95	***2.75E-09***	0.16	NA	NA	NA	NA
7	1	66,304,167	66,690,704	rs7519259	66,434,743	A	G	9.65	***4.72E-22***	NA	NA	NA	NA	NA
8	1	71,489,252	71,920,314	rs4650105	71,841,727	A	G	5.46	1.47E-07	NA	0.07	NA	***4.89E-08***	NA
9	1	72,749,726	72,954,641	rs9425089	72,765,082	A	C	−5.61	9.51E-07	0.01	NA	***2.07E-08***	NA	NA
10	1	73,275,828	74,131,749	rs12031120	73,860,821	A	G	−11.12	**3.83E-24**	8.61E-05	0.11	***1.02E-28***	**5.78E-24**	**1.03E-27**
**11**	1	79,278,918	79,488,107	rs12754818	79,392,756	A	C	−5.74	***9.60E-09***	NA	NA	NA	NA	NA
12	1	80,784,642	80,871,734	rs67263181	80,858,910	T	TA	−6.35	***2.13E-10***	NA	NA	NA	NA	NA
13	1	91,189,731	91,234,126	rs1526480	91,209,986	T	C	−6.86	**9.43E-12**	0.13	0.55	***6.93E-12***	NA	NA
14	1	97,490,949	97,605,851	rs79313673	97,595,236	T	C	5.59	1.18E-05	1.59E-03	0.06	5.47E-08	5.05E-06	***2.34E-08***
15	1	97,894,619	97,944,059	rs4950035	97,920,746	T	C	6.98	***3.02E-12***	NA	NA	NA	NA	NA
**16**	1	98,565,234	98,664,952	rs61787321	98,632,436	T	C	−5.45	***4.96E-08***	NA	NA	NA	NA	NA
17	1	165,086,665	165,126,202	rs10753661	165,119,792	A	G	−6.23	***4.66E-10***	NA	0.24	NA	**4.72E-10**	NA
18	1	171,809,976	171,906,903	rs6425479	171,881,168	A	G	−5.83	**2.80E-08**	0.01	NA	***5.63E-09***	NA	NA
19	1	173,773,928	175,003,074	rs2142633	174,515,935	A	G	6.47	***9.76E-11***	NA	0.85	NA	NA	NA
20	1	197,312,162	197,821,769	rs34451672	197,767,841	CT	C	−5.52	***3.41E-08***	NA	NA	NA	NA	NA
21	1	227,217,412	227,512,190	rs7556248	227,320,893	T	C	5.53	***3.20E-08***	0.16	NA	2.18E-07	NA	NA
22	1	236,868,733	236,905,585	rs4659711	236,902,364	T	C	5.90	**4.98E-09**	0.08	0.70	***3.55E-09***	NA	NA
23	2	1,896,243	1,966,952	rs10180442	1,958,049	T	C	−5.84	***5.22E-09***	NA	NA	NA	NA	NA
**24**	2	4,824,073	4,909,777	rs6725602	4,845,377	T	C	−5.47	***4.64E-08***	NA	NA	NA	NA	NA
25	2	22,045,717	22,174,521	rs368693555	22,129,730	T	C	−5.86	1.20E-06	2.80E-03	0.42	***4.77E-09***	1.39E-06	**5.99E-09**
26	2	22,621,296	22,918,025	rs1355175	22,761,803	T	C	−6.07	***1.29E-09***	NA	NA	NA	NA	NA
**27**	2	23,541,309	23,582,960	rs11674807	23,541,471	T	C	−5.81	**3.84E-08**	0.05	NA	***6.39E-09***	NA	NA
28	2	27,498,734	27,752,871	rs3739095	27,556,721	A	G	6.37	***1.84E-10***	NA	NA	NA	NA	NA
29	2	43,512,764	44,359,876	rs75120545	44,271,496	T	C	−6.38	***1.75E-10***	NA	0.94	NA	**3.03E-10**	NA
30	2	45,121,466	45,175,585	rs472140	45,139,904	T	C	10.91	***9.96E-28***	0.27	0.14	NA	**1.70E-27**	NA
**31**	2	56,616,218	57,427,953	rs72618684	56,828,238	T	C	−6.36	**8.48E-09**	NA	4.97E-05	NA	***2.08E-10***	NA
32	2	57,942,987	58,505,679	rs2678892	58,167,140	A	G	8.24	***1.72E-16***	NA	NA	NA	NA	NA
33	2	60,115,069	60,527,297	rs359257	60,485,161	A	C	−6.43	**2.68E-09**	0.02	NA	***1.24E-10***	NA	NA
34	2	61,429,568	61,843,183	rs778766	61,786,848	T	C	−5.59	***2.31E-08***	NA	NA	NA	NA	NA
35	2	62,710,273	62,780,325	rs56044100	62,735,706	A	G	−6.91	**2.71E-09**	3.97E-04	NA	***4.75E-12***	NA	NA
36	2	73,552,542	74,051,360	rs1246094	73,707,335	T	C	6.18	***6.52E-10***	NA	0.52	NA	**1.13E-09**	NA
37	2	101,233,020	101,311,552	rs4851350	101,234,362	T	C	5.98	**9.64E-09**	NA	0.04	NA	***2.28E-09***	NA
38	2	104,056,454	104,696,491	rs4851030	104,160,261	A	G	7.71	**1.51E-14**	NA	0.04	NA	***1.26E-14***	NA
39	2	105,915,711	105,994,827	rs4851758	105,952,417	T	C	6.26	2.63E-06	2.53E-05	0.24	***3.97E-10***	2.24E-06	**4.08E-10**
40	2	138,077,405	138,394,805	rs2375518	138,270,224	A	G	−5.82	**3.87E-08**	NA	0.03	NA	***6.00E-09***	NA
41	2	144,144,663	144,272,229	rs4233566	144,272,229	A	G	−7.00	***2.64E-12***	NA	NA	NA	NA	NA
42	2	145,341,259	145,568,183	rs72991962	145,420,704	A	C	−5.75	4.64E-07	8.50E-03	0.06	**2.47E-08**	1.44E-07	***8.73E-09***
43	2	147,828,502	147,981,914	rs72859290	147,981,914	A	G	6.73	***1.68E-11***	NA	NA	NA	NA	NA
**44**	2	156,884,809	156,933,105	rs72904197	156,887,825	A	G	6.02	***1.78E-09***	NA	NA	NA	NA	NA
45	2	159,310,846	159,539,677	rs6437184	159,354,852	T	C	−6.09	2.09E-06	1.04E-04	NA	***1.13E-09***	NA	NA
46	2	161,606,161	162,792,864	rs57761252	161,865,998	A	G	−7.07	**3.44E-12**	0.26	0.10	**3.55E-09**	***1.51E-12***	**8.88E-09**
47	2	164,733,530	164,928,899	rs268257	164,906,685	T	C	−5.72	***1.04E-08***	NA	NA	NA	NA	NA
48	2	178,565,913	178,608,411	rs17400325	178,565,913	T	C	6.54	***6.20E-11***	NA	NA	NA	NA	NA
49	2	185,541,022	186,057,716	rs1594166	185,787,586	T	G	−6.34	**9.97E-09**	0.01	NA	***2.36E-10***	NA	NA
50	2	201,080,113	201,244,261	rs3754797	201,242,293	A	G	7.48	**1.02E-11**	9.16E-03	0.04	**1.97E-13**	**4.00E-12**	***7.55E-14***
51	2	202,842,998	202,848,662	rs55846056	202,848,214	A	C	−5.61	***2.08E-08***	0.14	0.24	**3.35E-08**	**4.35E-08**	**3.11E-08**
**52**	2	208,219,840	208,300,941	rs13392734	208,255,975	A	C	−6.05	***1.46E-09***	NA	0.42	NA	NA	NA
53	2	226,336,692	226,400,416	rs6436561	226,370,779	A	G	6.21	**3.85E-08**	0.03	NA	***5.47E-10***	NA	NA
54	3	16,729,269	17,119,238	rs6800583	16,851,755	A	G	7.18	***6.77E-13***	NA	NA	NA	NA	NA
55	3	18,610,030	18,824,298	rs9835977	18,793,340	T	C	−7.01	***2.38E-12***	NA	NA	NA	NA	NA
56	3	24,902,655	24,923,743	rs12495571	24,902,655	T	C	−5.67	***1.47E-08***	NA	0.37	NA	**1.48E-08**	NA
57	3	48,412,246	49,940,078	rs7625857	49,596,462	T	C	−11.21	***3.60E-29***	NA	0.16	NA	**5.71E-29**	NA
58	3	53,840,207	53,858,541	rs3774611	53,840,665	A	G	−5.75	**4.96E-08**	NA	0.05	NA	***8.92E-09***	NA
59	3	55,936,417	56,414,387	rs7641315	55,987,823	A	G	−6.87	**8.13E-12**	NA	0.38	NA	***6.46E-12***	NA
**60**	3	59,777,008	59,786,032	rs2629879	59,777,685	A	G	−5.83	***5.71E-09***	NA	0.69	NA	NA	NA
61	3	81,236,994	81,985,189	rs6804453	81,873,269	T	C	−6.58	***4.85E-11***	NA	0.69	NA	**1.27E-10**	NA
62	3	84,841,679	84,951,716	rs112987098	84,883,600	CA	C	−7.31	***2.71E-13***	NA	NA	NA	NA	NA
63	3	85,481,557	86,209,081	rs7644873	85,720,888	T	C	−6.83	**4.11E-10**	0.01	NA	***8.68E-12***	NA	NA
**64**	3	98,489,915	98,770,189	rs9811449	98,499,298	T	G	5.59	**2.74E-08**	NA	0.22	NA	***2.32E-08***	NA
**65**	3	104,647,183	104,805,201	rs10804436	104,670,412	A	C	−5.69	***1.31E-08***	0.18	0.37	6.56E-08	**1.89E-08**	6.56E-08
66	3	110,103,471	110,300,607	rs6437918	110,225,767	T	C	6.39	**4.83E-09**	0.02	0.45	***1.70E-10***	**7.07E-09**	**2.42E-10**
67	3	114,135,165	114,233,728	rs7630111	114,165,901	A	C	7.63	***2.29E-14***	0.04	0.13	**3.84E-13**	**2.37E-14**	**1.61E-13**
68	3	147,095,294	147,268,599	rs2279829	147,106,319	T	C	−6.04	***1.54E-09***	NA	NA	NA	NA	NA
69	3	157,829,953	158,534,111	rs7629432	157,895,285	T	C	7.01	**6.60E-09**	4.74E-04	0.68	***2.34E-12***	**1.34E-08**	**1.21E-11**
70	3	161,703,030	161,801,528	rs9868656	161,797,034	T	C	5.58	4.43E-07	0.03	NA	***2.40E-08***	NA	NA
71	4	28,247,071	28,572,856	rs35236641	28,499,286	A	AG	−5.60	***2.11E-08***	NA	NA	NA	NA	NA
72	4	42,036,458	42,185,424	rs4861023	42,150,774	A	G	−5.63	2.26E-07	0.05	0.18	**3.48E-08**	8.81E-08	***1.78E-08***
73	4	46,879,127	47,035,087	rs10033762	46,907,959	T	G	−7.46	**9.82E-11**	5.17E-03	0.04	**1.15E-13**	**5.25E-11**	***8.88E-14***
**74**	4	59,864,305	60,078,065	rs78040411	59,919,626	T	C	5.85	***4.82E-09***	NA	NA	NA	NA	NA
**75**	4	62,007,737	62,098,937	rs1394950	62,083,448	T	C	−5.53	9.78E-07	8.47E-03	NA	***3.20E-08***	NA	NA
**76**	4	62,758,896	62,845,490	rs734644	62,800,728	T	C	−6.16	**2.30E-09**	0.15	NA	***7.21E-10***	NA	NA
**77**	4	85,485,247	85,965,865	rs111669166	85,485,793	A	G	−5.73	***1.03E-08***	NA	NA	NA	NA	NA
78	4	99,630,017	101,037,327	rs1229984	100,239,319	T	C	−9.48	NA	1.47E-05	***2.57E-21***	NA	NA	NA
79	4	102,702,364	103,387,161	rs13107325	103,188,709	T	C	−10.94	**9.04E-27**	0.41	2.18E-03	**9.76E-22**	***7.70E-28***	**2.81E-23**
80	4	112,208,748	112,503,872	rs72678859	112,406,961	T	C	−6.92	**2.18E-11**	0.11	0.73	***4.59E-12***	NA	NA
81	4	143,486,962	144,207,201	rs2120162	143,547,293	A	C	7.21	**2.48E-12**	NA	0.02	NA	***5.55E-13***	NA
82	4	151,296,089	151,936,204	rs12509782	151,605,828	A	G	−6.02	***1.70E-09***	NA	NA	NA	NA	NA
83	4	152,238,328	152,724,537	rs3792689	152,637,700	T	C	6.02	***1.70E-09***	NA	NA	NA	NA	NA
**84**	4	186,658,747	186,688,223	rs56110010	186,658,747	T	C	−5.92	**1.16E-08**	NA	0.04	NA	***3.19E-09***	NA
85	5	3,405,495	3,417,995	rs10462767	3,417,930	T	C	6.28	6.84E-07	2.10E-03	1.32E-03	**3.18E-09**	7.87E-08	***3.45E-10***
86	5	30,783,801	30,845,465	rs10940922	30,818,059	T	C	6.31	**5.75E-10**	NA	0.07	NA	***2.85E-10***	NA
87	5	43,054,747	43,193,052	rs10941611	43,162,608	T	C	−5.57	***2.54E-08***	NA	NA	NA	NA	NA
**88**	5	51,704,512	51,852,586	rs11742575	51,822,955	T	C	−6.44	**2.82E-10**	0.19	NA	***1.23E-10***	NA	NA
89	5	59,842,971	60,614,879	rs7709444	60,095,726	T	C	7.30	**5.44E-12**	0.05	0.01	**6.94E-13**	**9.64E-13**	***3.00E-13***
90	5	61,397,967	61,959,339	rs4700486	61,517,281	A	G	−5.99	***2.14E-09***	NA	NA	NA	NA	NA
91	5	153,303,901	153,626,546	rs2199626	153,507,230	T	C	−6.73	**4.68E-08**	7.83E-04	0.03	**3.16E-11**	**1.20E-08**	***1.72E-11***
92	5	166,985,224	167,055,936	rs1549212	166,996,722	T	C	−6.92	**9.65E-12**	0.02	NA	***4.49E-12***	NA	NA
93	5	170,284,624	170,846,151	rs4286697	170,454,681	A	G	−7.06	**1.43E-09**	6.20E-04	0.59	***1.63E-12***	**2.76E-09**	**3.47E-12**
94	6	11,470,694	11,614,620	rs821443	11,538,534	T	C	−5.49	1.08E-06	0.02	0.67	***4.05E-08***	1.69E-06	6.41E-08
**95**	6	19,212,286	19,307,114	rs1858513	19,228,938	T	C	5.74	***9.58E-09***	NA	0.79	NA	**2.20E-08**	NA
96	6	25,480,328	29,607,101	rs71559024	27,352,750	A	G	−8.42	***3.92E-17***	0.01	NA	**1.04E-15**	NA	NA
97	6	33,203,908	33,306,235	rs464921	33,240,506	A	G	−5.69	***1.29E-08***	NA	0.08	NA	NA	NA
98	6	37,609,457	37,632,322	rs3846873	37,619,600	A	G	5.46	8.66E-06	9.20E-03	0.02	2.40E-07	1.41E-06	***4.68E-08***
**99**	6	47,802,504	47,885,971	rs559028	47,809,908	T	C	6.57	***5.18E-11***	NA	0.82	NA	NA	NA
100	6	126,657,472	127,080,700	rs9398809	126,789,144	T	C	−5.73	2.95E-07	0.05	0.03	**3.17E-08**	9.01E-08	***9.79E-09***
101	6	143,141,800	143,197,874	rs72995085	143,193,971	T	C	5.50	***3.82E-08***	NA	NA	NA	NA	NA
102	6	152,201,201	152,264,529	rs6557171	152,234,593	T	C	5.52	***3.33E-08***	NA	NA	NA	NA	NA
103	6	154,215,240	154,390,607	rs1799971	154,360,797	A	G	7.85	**4.52E-14**	0.12	1.80E-03	**5.53E-13**	***4.03E-15***	**4.56E-14**
104	6	162,931,993	163,209,495	rs62430881	162,988,202	A	C	−5.87	**4.29E-08**	NA	2.46E-03	NA	***4.28E-09***	NA
105	6	163,792,420	164,006,012	rs1764021	163,805,682	T	C	−6.67	***2.64E-11***	NA	NA	NA	NA	NA
106	6	169,799,110	170,248,792	rs4236177	170,073,543	T	C	6.14	**1.89E-09**	NA	0.10	NA	***8.05E-10***	NA
107	7	75,607,155	75,910,452	rs6467958	75,622,281	T	C	−6.86	**1.91E-11**	NA	0.09	NA	***6.93E-12***	NA
108	7	97,264,194	97,310,892	rs10275909	97,298,821	A	G	−5.63	**3.48E-08**	NA	0.07	NA	***1.79E-08***	NA
109	7	109,928,395	110,028,559	rs78971860	109,989,952	T	C	−5.62	3.53E-06	4.83E-03	NA	***1.88E-08***	NA	NA
110	7	113,998,993	114,287,116	rs2189010	114,119,430	A	G	7.88	***3.19E-15***	NA	NA	NA	NA	NA
111	7	114,940,159	115,025,708	rs1155397	114,953,597	A	G	7.74	**1.38E-14**	NA	0.09	NA	***1.02E-14***	NA
112	7	117,497,811	117,593,308	rs6965740	117,514,840	T	G	−6.72	***1.87E-11***	NA	NA	NA	NA	NA
113	7	135,050,259	135,221,170	rs11772832	135,073,047	T	C	−8.36	**1.33E-16**	NA	0.26	NA	***6.12E-17***	NA
114	8	9,894,125	10,005,367	rs7832431	9,947,661	A	C	5.96	2.22E-07	4.35E-03	NA	***2.54E-09***	NA	NA
115	8	12,662,159	12,694,291	rs7812870	12,667,421	T	C	−5.99	7.66E-07	6.08E-04	NA	***2.06E-09***	NA	NA
116	8	21,770,372	21,872,853	rs2054711	21,818,060	A	G	6.97	**3.96E-09**	7.83E-04	0.18	**5.66E-12**	**3.07E-09**	***3.28E-12***
117	8	27,406,353	27,470,919	rs11783093	27,425,349	T	C	−7.82	***5.51E-15***	NA	0.17	NA	NA	NA
118	8	52,573,769	52,870,877	rs2395827	52,786,700	T	C	−5.52	4.54E-06	0.02	0.01	1.47E-07	1.04E-06	***3.36E-08***
119	8	57,221,079	57,437,154	rs2246873	57,433,941	A	G	−7.21	***5.68E-13***	NA	NA	NA	NA	NA
120	8	64,505,710	65,097,747	rs7017705	64,982,843	T	C	6.77	**3.89E-11**	NA	0.05	NA	***1.28E-11***	NA
121	8	76,705,544	77,021,947	rs830432	76,941,945	A	G	−5.57	2.64E-06	6.41E-03	0.19	**3.49E-08**	1.84E-06	***2.50E-08***
**122**	8	77,588,548	77,695,732	rs76132822	77,679,194	T	C	5.51	1.42E-07	NA	0.08	NA	***3.57E-08***	NA
**123**	8	87,139,254	87,241,715	rs2954344	87,238,494	T	G	−5.63	1.35E-05	3.00E-04	NA	***1.80E-08***	NA	NA
124	8	92,976,563	93,180,965	rs9297901	93,036,795	T	C	7.03	**2.95E-12**	0.01	NA	***2.10E-12***	NA	NA
125	8	113,811,442	114,725,064	rs6469450	114,179,599	A	C	7.18	***6.80E-13***	NA	NA	NA	NA	NA
**126**	8	142,021,655	142,031,024	rs55793749	142,028,409	A	G	−5.78	***7.31E-09***	NA	NA	NA	NA	NA
**127**	8	143,345,007	143,354,963	rs7014279	143,352,528	A	C	5.62	***1.96E-08***	NA	NA	NA	NA	NA
128	9	16,089,196	16,102,759	rs62552202	16,092,730	A	C	6.00	**2.44E-08**	0.03	0.42	***2.00E-09***	NA	NA
**129**	9	17,044,769	17,128,036	rs425455	17,061,693	T	C	−5.72	***1.07E-08***	NA	NA	NA	NA	NA
130	9	23,747,791	23,849,297	rs9298850	23,765,511	T	C	−6.30	**1.13E-09**	NA	0.07	NA	***3.05E-10***	NA
131	9	30,121,131	30,456,131	rs12348434	30,247,380	A	G	−6.47	**5.12E-09**	0.02	0.21	***9.97E-11***	**4.99E-09**	**1.43E-10**
132	9	81,331,339	81,508,309	rs7029483	81,483,995	A	G	−5.89	**4.59E-08**	NA	9.76E-04	NA	***3.93E-09***	NA
133	9	127,594,420	128,411,969	rs4837011	127,923,014	T	G	−9.73	***2.27E-22***	0.14	0.14	**2.72E-21**	**3.75E-22**	**1.23E-20**
**134**	9	134,267,022	134,269,789	rs182293592	134,269,789	T	G	5.58	***2.47E-08***	NA	NA	NA	NA	NA
135	9	134,802,170	134,946,805	rs9411334	134,896,887	T	C	−5.78	**1.58E-08**	NA	0.07	NA	***7.49E-09***	NA
136	9	136,876,021	136,973,826	rs467387	136,907,005	A	G	5.96	**3.65E-08**	7.54E-03	NA	***2.51E-09***	NA	NA
137	10	10,559,149	10,700,036	rs7924306	10,573,209	T	G	5.63	6.13E-07	0.02	NA	***1.78E-08***	NA	NA
138	10	64,874,754	65,400,271	rs7075302	65,381,740	A	G	6.07	1.57E-07	7.56E-03	0.14	***1.30E-09***	NA	NA
139	10	75,430,874	75,661,162	rs12775558	75,570,868	A	G	6.41	**8.35E-09**	0.01	NA	***1.47E-10***	NA	NA
**140**	10	80,094,714	80,159,503	rs938244	80,113,953	T	C	−5.67	1.97E-07	0.08	0.06	**3.41E-08**	8.44E-08	***1.46E-08***
**141**	10	84,735,682	84,968,882	rs10885703	84,843,021	T	C	5.63	***1.80E-08***	NA	0.41	NA	**2.49E-08**	NA
142	10	102,912,264	105,165,256	rs1046411	104,837,816	A	G	6.36	1.03E-07	4.95E-03	9.72E-03	**9.63E-10**	**2.12E-08**	***1.96E-10***
143	10	110,461,674	110,758,956	rs7074871	110,507,806	A	G	−9.04	**8.20E-14**	9.85E-06	3.90E-03	**4.19E-19**	**1.02E-14**	***1.53E-19***
**144**	10	118,902,004	118,919,575	rs73385301	118,909,289	A	G	6.24	**1.43E-09**	NA	0.09	NA	***4.37E-10***	NA
**145**	11	24,932,312	25,098,152	rs12278803	24,941,827	A	G	5.66	***1.55E-08***	NA	NA	NA	NA	NA
146	11	27,646,247	27,736,207	rs1808124	27,686,904	T	C	5.49	***4.01E-08***	NA	NA	NA	NA	NA
147	11	27,996,573	28,709,434	rs10835368	28,643,299	T	C	−6.22	**9.78E-10**	NA	0.12	NA	***5.15E-10***	NA
148	11	38,401,216	38,998,930	rs2007212	38,490,584	T	C	−6.48	***9.35E-11***	NA	0.86	NA	NA	NA
149	11	46,336,995	46,751,495	rs7476	46,342,834	A	C	−5.86	***4.61E-09***	0.17	NA	5.99E-08	NA	NA
150	11	47,372,377	47,946,836	rs7107356	47,676,170	A	G	−6.70	**6.03E-10**	0.02	0.40	***2.10E-11***	**8.80E-10**	**2.93E-11**
151	11	57,385,856	57,681,828	rs12785533	57,486,651	A	G	7.31	***2.61E-13***	NA	NA	NA	NA	NA
152	11	63,869,596	64,218,143	rs113421210	64,133,658	T	G	7.58	**5.77E-13**	0.25	0.01	**3.89E-12**	***3.47E-14***	**3.80E-12**
153	11	73,305,859	73,312,252	rs4433590	73,310,668	A	G	−5.78	***7.34E-09***	NA	0.25	NA	**1.77E-08**	NA
154	11	88,470,041	89,055,293	rs681820	88,615,547	A	G	−5.99	***2.08E-09***	NA	0.72	NA	NA	NA
155	11	95,440,480	95,681,028	rs1150361	95,633,805	A	G	−7.38	**1.19E-11**	0.01	5.02E-03	**8.74E-13**	**1.36E-12**	***1.54E-13***
156	11	112,826,311	114,008,053	rs61902812	113,374,420	A	C	−11.81	**1.40E-29**	4.44E-03	2.26E-03	**3.66E-32**	**8.50E-30**	***3.40E-32***
157	11	116,073,168	116,103,543	rs10891932	116,103,543	A	G	7.03	**1.18E-10**	3.94E-03	3.99E-03	**1.63E-11**	**1.30E-11**	***2.15E-12***
158	11	121,495,141	121,662,150	rs1944678	121,646,580	A	C	9.44	**5.40E-13**	**9.49E-09**	0.02	**5.25E-20**	**4.57E-14**	***3.69E-21***
159	11	126,898,083	126,998,347	rs12362296	126,983,715	A	G	−6.44	***1.17E-10***	NA	NA	NA	NA	NA
160	11	132,038,207	132,321,312	rs11223043	132,254,814	A	G	−5.94	7.15E-07	5.99E-03	0.05	**4.64E-09**	2.86E-07	***2.90E-09***
**161**	11	133,303,819	133,370,476	rs6590712	133,349,706	A	C	5.68	2.50E-06	9.08E-04	0.34	**2.28E-08**	2.54E-06	***1.33E-08***
162	12	2,285,731	2,402,246	rs11062148	2,298,085	T	C	5.51	***3.57E-08***	NA	0.46	NA	NA	NA
163	12	51,780,136	51,919,819	rs10783447	51,884,821	A	G	−8.29	**3.13E-16**	NA	0.02	NA	***1.10E-16***	NA
164	12	53,730,164	54,028,059	rs61928096	53,780,633	A	G	5.46	6.75E-07	0.05	0.36	7.73E-08	5.23E-07	***4.70E-08***
165	12	81,469,429	81,673,764	rs35166008	81,571,248	A	G	−6.08	***1.22E-09***	NA	NA	NA	NA	NA
166	12	83,863,748	84,165,561	rs1599264	84,122,022	A	G	−5.49	***4.07E-08***	NA	NA	NA	NA	NA
167	12	110,501,407	111,281,108	rs7971825	111,061,652	A	G	5.59	***2.22E-08***	NA	0.73	NA	NA	NA
168	12	120,879,294	121,185,145	rs12829722	121,155,622	T	C	6.09	2.53E-06	4.64E-04	0.02	**3.44E-09**	6.98E-07	***1.17E-09***
**169**	12	122,154,617	122,203,680	rs28651018	122,186,268	T	C	−5.89	***3.93E-09***	7.92E-03	NA	NA	NA	NA
170	13	55,603,644	56,250,005	rs145720647	55,708,612	A	G	8.19	**3.18E-12**	2.45E-04	0.02	**3.69E-16**	**7.32E-13**	***2.64E-16***
**171**	13	79,855,297	80,141,911	rs9318638	79,986,153	T	C	−6.15	2.18E-07	1.65E-03	NA	***7.65E-10***	NA	NA
172	13	96,589,737	97,029,792	rs4771935	96,996,902	A	C	−7.28	**4.89E-10**	1.85E-03	0.06	***3.38E-13***	**2.22E-10**	**4.52E-13**
173	14	41,952,029	42,183,025	rs12431444	42,068,689	T	C	6.21	**3.63E-09**	NA	3.45E-03	NA	***5.19E-10***	NA
174	14	47,243,109	47,409,360	rs12889080	47,291,881	T	C	5.50	4.52E-07	NA	1.46E-03	NA	***3.76E-08***	NA
175	14	55,569,578	55,936,577	rs17743595	55,901,926	A	G	5.50	2.82E-07	0.04	0.51	***3.81E-08***	4.06E-07	5.93E-08
176	14	57,338,977	57,381,487	rs864279	57,349,958	T	C	7.11	***1.20E-12***	NA	0.31	NA	NA	NA
177	14	58,664,909	58,856,709	rs6573198	58,759,762	A	G	−6.74	**5.06E-11**	0.08	0.23	**1.70E-11**	**5.29E-11**	***1.59E-11***
178	14	79,453,862	79,614,043	rs10143998	79,525,531	T	C	−6.70	6.10E-08	1.18E-04	0.29	***2.16E-11***	5.85E-08	**2.34E-11**
179	14	99,733,954	99,750,520	rs1405238	99,733,954	T	C	6.14	***8.04E-10***	NA	NA	NA	NA	NA
180	14	104,021,141	104,363,528	rs6576006	104,322,394	A	C	6.81	**1.68E-11**	5.30E-03	NA	***9.79E-12***	NA	NA
181	15	47,613,403	47,971,793	rs175979	47,674,812	A	G	9.29	**5.62E-18**	1.34E-03	NA	***1.59E-20***	NA	NA
**182**	15	82,443,939	82,537,354	rs7162177	82,474,069	T	C	6.34	***2.26E-10***	NA	NA	NA	NA	NA
183	15	83,413,041	83,977,166	rs35563832	83,904,049	T	G	−7.52	***5.45E-14***	NA	0.75	NA	**4.25E-13**	NA
184	15	91,416,550	91,429,042	rs4702	91,426,560	A	G	−6.26	***3.76E-10***	NA	NA	NA	NA	NA
185	16	13,580,639	13,763,942	rs12925872	13,752,356	T	C	5.67	3.79E-06	4.91E-03	0.03	**3.66E-08**	1.25E-06	***1.47E-08***
**186**	16	19,246,136	19,279,464	rs12921478	19,272,075	A	C	−6.75	***1.45E-11***	NA	0.53	NA	NA	NA
**187**	16	23,758,347	23,901,376	rs194795	23,843,121	T	G	−5.80	***6.55E-09***	NA	NA	NA	NA	NA
188	16	29,924,422	30,120,442	rs8060511	30,101,596	A	C	−7.71	**7.55E-14**	0.05	0.35	***1.26E-14***	**4.69E-14**	**1.96E-14**
189	16	53,797,908	53,848,561	rs56137030	53,825,905	A	G	−10.12	**8.17E-23**	0.01	0.12	***4.35E-24***	**1.14E-22**	**2.39E-23**
**190**	16	61,583,172	61,650,572	rs9635513	61,631,362	T	C	5.50	1.52E-07	0.10	NA	***3.72E-08***	NA	NA
191	16	72,052,034	72,249,332	rs8047881	72,193,906	A	G	−5.54	5.41E-07	0.04	NA	***3.12E-08***	NA	NA
192	16	73,594,142	73,606,553	rs825704	73,603,649	T	C	5.66	3.24E-07	0.02	0.21	**1.70E-08**	1.82E-07	***1.48E-08***
193	16	76,419,114	76,519,827	rs1506812	76,507,956	A	C	−6.32	**3.09E-09**	0.04	NA	***2.62E-10***	NA	NA
194	16	87,430,239	87,518,815	rs3748400	87,445,839	T	C	−5.54	2.41E-07	NA	0.02	NA	***3.04E-08***	NA
195	17	7,802,968	7,924,748	rs12943962	7,818,004	T	G	−5.55	9.54E-07	0.02	0.72	***2.88E-08***	1.63E-06	**2.89E-08**
**196**	17	18,040,690	18,128,474	rs854784	18,040,690	T	C	6.34	**1.17E-08**	0.01	0.01	***2.34E-10***	NA	NA
197	17	29,389,026	29,735,829	rs2525570	29,681,245	A	G	−6.42	**1.71E-09**	0.03	NA	***1.33E-10***	NA	NA
198	17	44,040,626	44,056,097	rs3785883	44,054,433	A	G	5.63	**3.28E-08**	0.13	0.37	**2.92E-08**	**2.76E-08**	***1.80E-08***
**199**	17	48,200,834	48,234,411	rs847680	48,224,075	T	C	−5.55	2.35E-07	0.13	NA	***2.91E-08***	NA	NA
200	17	55,655,932	55,675,058	rs72833102	55,663,552	A	G	7.19	**1.71E-12**	NA	0.13	NA	***6.62E-13***	NA
201	17	65,822,573	66,098,979	rs62084675	66,027,025	A	G	5.86	***4.62E-09***	NA	1.82E-03	NA	NA	NA
202	18	35,125,113	35,204,858	rs17570353	35,154,734	A	G	6.33	**4.38E-09**	0.05	NA	***2.52E-10***	NA	NA
203	18	39,253,947	39,322,811	rs112226573	39,305,644	T	TCTC	5.49	***4.04E-08***	NA	NA	NA	NA	NA
204	18	50,555,225	51,054,765	rs35916734	50,762,061	A	ATT	6.27	***3.59E-10***	NA	NA	NA	NA	NA
**205**	18	52,369,516	52,523,477	rs60512641	52,523,477	A	G	7.19	**2.77E-12**	3.68E-03	NA	***6.51E-13***	NA	NA
206	18	53,195,249	53,463,661	rs12458015	53,305,735	T	C	−6.06	**3.77E-09**	0.11	NA	***1.40E-09***	NA	NA
207	18	77,551,586	77,580,712	rs11664298	77,578,986	A	G	5.64	***1.69E-08***	NA	NA	NA	NA	NA
208	19	612,234	637,909	rs41542013	623,728	T	C	−5.69	6.40E-06	2.07E-03	0.07	**4.37E-08**	1.62E-06	***1.27E-08***
209	19	5,033,752	5,084,579	rs16992771	5,046,070	T	C	−6.32	***2.57E-10***	NA	0.94	NA	**9.20E-10**	NA
210	19	10,744,807	10,853,296	rs10405617	10,752,968	A	G	−6.22	***5.13E-10***	0.26	NA	**9.54E-10**	NA	NA
211	19	18,519,997	18,637,194	rs2278238	18,576,484	T	C	−6.22	2.28E-06	3.28E-05	0.35	***5.00E-10***	2.19E-06	**5.64E-10**
212	19	19,355,537	19,643,907	rs12463074	19,445,869	A	G	5.49	1.67E-07	NA	0.06	NA	***4.12E-08***	NA
213	19	49,168,942	49,254,955	rs2548458	49,209,325	T	C	7.52	**1.41E-12**	0.02	0.23	***5.59E-14***	**1.52E-12**	**1.37E-13**
214	20	31,093,514	31,187,504	rs911527	31,165,105	T	C	5.78	***7.41E-09***	NA	NA	NA	NA	NA
215	20	41,956,451	42,016,520	rs6030812	42,001,011	A	G	−6.81	**2.99E-10**	0.04	NA	***9.54E-12***	NA	NA
216	20	48,435,242	48,645,999	rs7272308	48,583,726	A	G	6.28	**5.61E-09**	NA	3.88E-03	NA	***3.45E-10***	NA
217	21	40,512,918	40,718,354	rs114399588	40,653,958	A	G	6.22	4.07E-07	3.69E-03	0.49	***5.10E-10***	NA	NA
218	21	46,485,406	46,551,702	rs2150438	46,485,941	A	G	6.52	**5.04E-10**	6.45E-03	NA	***7.28E-11***	NA	NA
219	22	42,363,812	42,690,311	rs9306356	42,567,090	T	C	5.85	**1.02E-08**	NA	0.26	NA	***4.84E-09***	NA
**220**	22	49,324,829	49,355,688	rs5770456	49,349,639	T	G	−5.78	***7.60E-09***	NA	0.30	NA	NA	NA

Significant P-values are in bold. The lowest P-values in each locus is italicized. Bolded loci are those not reported as associated with any SUD related phenotype in the GWAS catalog.

*Chr* chromosome; *BP* base pair positions; *Z* Z score corresponding to the lowest P-value; *EUR* 1kg-EUR-like; *AFR* 1kg-AFR-like; *AMR* 1kg-AMR-like; *AFR.EUR* 1kg-AFR + EUR-like; *AMR.EUR* 1kg-AMR + EUR-like; *AFR.AMR.EUR* 1kg-AFR + AMR + EUR-like.

By design, we only included variants with concordant effects; however, some concordant GWS variants also showed certain degree of associations (e.g., P-value <0.05) across all SUDs. For example, there were 2999 (1kg-EUR-like), 8 (1kg-AFR-like), 11 (1kg-AFR + EUR-like), and 146 (1kg-AMR + EUR-like) GWS variants with P-values <0.05 in all SUD GWAS (Supplemental Table 2, 3, 5, and 6). No GWS variants had P-values <0.05 in all SUD GWAS in the 1kg-AMR-like and 1kg-AFR + AMR + EUR-like samples.

### Gene-based analysis

We used MAGMA [33] and identified 494 significant genes in the 1kg-EUR-like sample, one significant gene in the 1kg-AFR-like sample, four significant genes in the 1kg-AMR-like sample, 552 significant genes in the 1kg-AFR + EUR-like samples, 492 significant genes in the 1kg-AMR + EUR-like samples, and 526 significant genes in the 1kg-AFR + AMR + EUR-like samples (Supplement tables 8–13). No gene was significant in more than one population. In total, 637 unique genes were identified by gene-based analysis. Manhattan plots of gene-based analyses are shown in Supplemental Fig. 2.

### Mapping significant variants to genes

We used positional mapping, eQTL mapping, and chromatin interaction mapping and identified 711 genes in the 1kg-EUR-like sample, seven genes in the 1kg-AFR-like sample, six genes in the 1kg-AMR-like sample, 495 genes in the 1kg-AFR + EUR-like samples, 482 in 1kg-AMR + EUR-like samples, and 373 genes in the 1kg-ARF + AMR + EUR-like samples (Supplemental Tables 14–18). In total, we identified 837 unique genes.

### Gene prioritization

We used a combination of gene-based analysis and gene mapping and identified a total of 1,044 genes. Among them, 785 genes were prioritized, which included 702 protein coding genes, 35 non-coding RNAs, 31 antisense genes, and 12 pseudogenes (Supplemental Table 19). Three genes were prioritized in both the 1kg-EUR-like and 1kg-AMR-like samples (*ADH1C*, *ADH5*, and *RP11-696N14.1*), and no other genes were prioritized in more than one population.

### Brain dissection and cell type enrichment analyses

Of the 785 prioritized genes, they were highly expressed in 48 dissections with one from amygdala, two from basal nuclei, 26 from cortex, one from epithalamus, five from hippocampus, four from hypothalamus, one from midbrain, two from paleocortex, and six from thalamus (Supplemental Table 20). Prioritized genes with low expression levels were observed in 17 dissections with one from basal forebrain, two from basal nuclei, three from cerebellum, two from hippocampus, two from midbrain, three from myelencephalon, one from perirhinal cortex, two from pons, and one from spinal cord (Supplemental Table 21). Prioritized genes were highly expressed in 251 of the 461 cell types with all but four in neuronal cells (Supplemental Table 22). Most of these cell types were from amygdala, basal forebrain, cerebral cortex, hippocampus, hypothalamus, and thalamus, which is in agreement with what was observed in the dissection enrichment analysis. Prioritized genes with low expression levels were observed in three cell types (astrocyte, microglia, and vascular) (Supplemental Table 23).

### SNP-heritability estimation and calculating SNP-heritability explained by concordant variants for each SUD

There were 799,022 concordant variants (representing 11.76, 15.17, 11.26, and 9.97% of the total number of variants in PAU, OUD, CUD, and TUD, respectively) in the 1kg-EUR-like sample. Using these concordant variants, we estimated a SNP-heritability of SUD (using LD score regression [41]) of 0.10 (SE = 0.006). These concordant variants explained 77.96%, 95.95%, 83.88%, and 56.01% of SNP-heritability of PAU, OUD, CUD and PTU, respectively in the 1kg-EUR-like sample (Supplemental Table 24).

### Genetic correlations

We identified 567 significant correlations in our cross SUD meta-analysis in the 1kg-EUR-like sample (Supplemental Table 25). Among those correlations, 24 were related substance use, 35 were related to psychiatric diseases, 46 were related to behavior, and six were related to life events.

### Polygenic score analyses

**Fig. 2:**
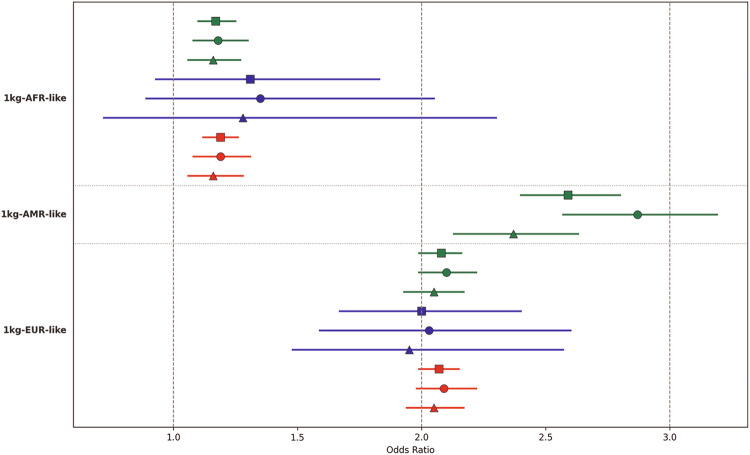
PGS analyses results. Odds ratios were calculated by comparing the top 10% of individuals with the highest PGS to the remaining 90% in the group. Green: AOU; blue: IB; red: meta-analysis; square: male and female combined; circle: females only; triangle: males only. Lines indicate 95% confidence interval.

In the 1kg-AFR-like sample, there were 69,159 individuals in the AOU dataset (12,353 cases) and 1829 in the IB dataset (912 cases). In the 1kg-AMR-like sample, the AOU dataset included 62,489 individuals (5925 cases). In the 1kg-EUR-like sample, the AOU dataset comprised 209,615 individuals (20,910 cases) and the IB dataset included 6375 individuals (2801 cases) (Supplemental Table 26). PGS were significant in all analyses except in the 1kg-AFR-like IB samples (Fig. 2, Supplemental Table 26). In both the 1kg-AMR-like and 1kg-EUR-like samples, individuals in the top 10% of genetic risk were approximately twice as likely to have SUDs as compared to those in the remaining 90%, across both the AOU and IB datasets. This pattern was consistent in analyses combining males and females, as well as in sex-stratified analyses, with odds ratios (OR) ranging from 1.95 to 2.87. PGS were slightly higher in females than in males, but the 95% CIs overlapped. In the meta-analyses, all PGS were significant, and results were similar to AOU.

### Drug repurposing

By querying DGIdb [50], we identified 260 FDA approved drugs with ATC code N targeting 51 genes (Supplemental Table 27). Seven drugs met the criteria for repurposing: Topiramate in ATC N03AX (treating epilepsy, targeting *GABRA4*); Aripiprazole and Cariprazine in ATC N05AX (treating schizophrenia, bipolar disorder, and major depressive disorder, targeting *DRD2*); Desipramine, Imipramine, and Nortriptyline in ATC N06AA (treating depression and anxiety, targeting *BDNF*); and Methylphenidate in ATC N06BA (treating ADHD, targeting *BDNF*). In the large-scale real-world data from the Clinformatics® database, users of these medications exhibited lower hazard ratios for developing SUDs compared to users of comparator drugs (Supplemental Table 28). For example, after adjusting for comorbidities, the hazard ratios were 0.44 for users of Topiramate (95% CI: 0.42–0.47), 0.88 for users of Aripiprazole or Cariprazine (95% CI: 0.78–0.88), 0.89 for users of Desipramine, Imipramine, or Nortriptyline (95% CI: 0.84–0.94); and 0.84 for users of Methylphenidate (95% CI: 0.78–0.91). These findings suggest that these seven drugs may have potential for repurposing in the treatment of SUDs.

## Discussion

In this study, we identified 220 SUD-shared loci in multiple populations, with the majority of variants detected in the 1kg-EUR-like population, likely due to its substantially larger sample size. Of these loci, 40 had not been previously reported as SUD related in the GWAS catalog [38] and thus represent novel associations. All identified loci showed consistent directions of effect across individual SUDs, supporting their role as shared genetic risk factors. Through gene-based analyses, gene mapping, and gene prioritization, we identified 785 SUD-shared genes. These genes are highly expressed in amygdala, cortex, hippocampus, hypothalamus, and thalamus, primarily in neuronal cells. Identified concordant variants explained 56–96% of SNP-heritability of each SUD in the 1kg-EUR-like sample. In the 1kg-EUR-like and 1kg-AMR-like samples, the top 10% of individuals with the highest PGS were approximately twice as likely to have an SUD as compared to the remaining 90% in their groups. Lastly, we identified seven FDA approved drugs to treat other diseases that could potentially be repurposed to treat SUDs.

Identifying genes shared across different SUDs is challenging due to the heterogeneity of individual SUD diagnoses, which are each based on multiple criteria. Combining SUDs as one phenotype to identify SUD-shared gene make it even more heterogenous. Consequently, some findings may be SUD-specific but could be mistakenly identified as SUD-shared due to their small P-values. Since SUD-specific variants may have discordant effects in other SUDs, only considering concordant variants removed most of them. Additionally, we did not use LD score regression to estimate the common factor, and thereby avoided findings due to model misspecifications or LD pattern mismatches and increased the statistical power. We validated our findings through the out-of-sample PGS analyses using two independent samples. The PGS had high predictive power in the 1kg-EUR-like and 1kg-AMR-like samples, demonstrating that the concordant variants included in calculating PGS were likely SUD-shared. For comparison purposes, we also performed cross SUD GWAS by using genomic SEM in 1kg-EUR-like sample (data not shown). Genomic SEM identified 161 loci and 186 lead variants, fewer than those identified by using meta-analysis but only considering concordant variants (184 loci and 428 lead variants). For those top 10% having the highest PGS calculated using genomic SEM results, the OR was 1.77 (95% CI: 1.47–2.12) in the IB 1kg-EUR-like sample, which was smaller than the OR estimated by using concordant variants from meta-analysis (OR = 2.0, 95% CI: 1.67–2.40).

We prioritized 785 genes. The large numbers of genes are expected as SUDs are highly polygenic [25, 26, 60–65]. All prioritized genes were expressed in brain tissue and associated with psychiatric or brain related traits, making them promising targets for functional validation studies to investigate their putative roles in SUD. When calculating genetic correlations using LD score regression, all variants included are assumed to have contributions to the trait [18]; therefore, by retaining concordant variants, most irrelevant variants were excluded from LD score regression and thus increased the power and accuracy of genetic correlation analyses. Some of the identified genetic correlations are worth noting. For instance, while traits such as going to the pub or social club were positively correlated with SUDs (genetic correlation (rG) = 0.67, P-value = 3.30E-41), traits such as going to a sports club or the gym, or attending group activities such as adult education classes and religious groups were negatively correlated with SUDs (rG < = −0.40, P-value <3.65E-09). This suggests that the latter leisure/social activities can be used to reduce the incidence of SUDs. In addition, maternal smoking around birth was highly positively correlated with SUDs (rG = 0.91, P-value = 2.28E-125), indicating a significant maternal genetic contribution to the future development of SUDs in offspring.

Koob and Volkow proposed a heuristic framework of SUDs based on animal studies and human brain imaging data [3]. The framework proposed three brain regions related to three stages of SUDs: prefrontal cortex (preoccupation/anticipation stage), basal ganglia (binge/intoxication stage), and extended amygdala (withdrawal/negative affect stage) [3]. We found that our prioritized genes were highly expressed in prefrontal cortex, in support of the proposed framework. However, we also observed that among all 34 dissections from cortex, prioritized genes were highly expressed in 26 of them. This suggests that most parts of the cortex may be related to SUDs, not just the prefrontal cortex. We found prioritized genes were highly expressed in caudate and putamen, but not other basal nuclei dissections. Prioritized genes were highly expressed in one amygdala dissection but not in the extended amygdala. Finally, while prioritized genes were not highly expressed in some basal nuclei dissections and extended amygdala, they were not expressed at particularly low levels in these regions either. Therefore, they were not necessarily unrelated to SUDs but may play less important roles in SUDs than other regions genetically (but may still play important roles via non-genetic mechanisms). Collectively, our findings indicate that more brain regions may be related to SUDs than previous studies have suggested [3]. We found that prioritized genes were highly expressed mostly in neuronal cells, indicating that the expression of SUD related genes is also cell type specific. To the best of our knowledge, this is the first comprehensive study of SUD-shared genes in different cell types in human brain. Our work sheds light on the etiologies of SUDs and further research is needed to confirm our findings.

In the 1kg-EUR-like and 1kg-AMR-like samples, the ORs for developing SUDs when comparing the top 10% individuals with the highest PGS to the remaining samples were approximately 2 or greater. These are the first SUD PGS that meet the suggested threshold for incorporation into clinical settings to provide additional information for the risk evaluation [66, 67]. In the 1kg-AFR-like sample, although PGS were statistically significant in both the AOU dataset and meta-analyses, the observed ORs ranged between 1.16 and 1.19, indicating that substantially larger sample sizes are needed to improve power. It is worth noting that the size of the 1kg-AMR-like sample was much smaller than that of the 1kg-AFR-like sample, but their PGS had much higher predictive power. One possible explanation for this result is that the 1kg-AMR-like sample is genetically closer to the 1kg-EUR-like sample, and thus using the 1kg-EUR-like sample GWAS significantly increased the statistical power. PGS meta-analysis results were similar to those in the AOU dataset. This is expected as the AOU dataset had much larger sample sizes than the IB dataset. In all analyses, PGS had slightly better predictive power in females than in males. Since PGS were calculated using autosomal variants only; we expected the PGS to perform similarly in males and females. The slight difference in our analyses and the different prevalence could be due to variants on the X and Y chromosomes, but more importantly, interactions between genetic and environmental factors. Adjusting PGS in epidemiological studies could help identify non-genetic factors that reduce the incidence of SUDs in females and thus facilitate the development of novel prevention strategies.

In the 1kg-EUR-like and 1kg-AFR-like samples, for those at high genetic risk, early interventions could be applied to reduce the incidence of SUDs. Additionally, PGS can be measured at any time, even before the initiation of risky substance use or risky behavior to further improve the effectiveness of prevention/intervention programs. PGS can also be used to stratify patients based on their risk levels to provide personalized treatments and improve the probability of recovery. That said, we want to caution readers that the predictive power of PGS is bounded by the estimated heritability of SUDs (~50%). Therefore, only using PGS will generate unreliable risk estimates as environmental factors are not considered. To reliably predict the risk for SUDs, both PGS and environmental factors, as well as their interactions should be carefully considered.

We used a large-scale real-world dataset and a robust active comparator design to identify repurposable drugs. For those seven drugs identified, previous studies have demonstrated their potential in treating just one or a few specific SUDs [51–58]—for example, Topiramate has been shown to reduce alcohol consumption [51, 68]. In contrast, our results suggest that these drugs may have broader therapeutic potential across all SUDs, as they target genes shared among multiple SUDs. This is especially crucial for affected individuals suffering from comorbid SUDs. It is worth noting that with the exception of Topiramate, the other six drugs target *BDNF* and *DRD2*-- genes that are also targeted by FDA-approved medications to treat substance-specific SUDs such as Bupropion (tobacco), Disulfiram (alcohol), and Methadone (opioids). This may facilitate their approval process, and these drugs should be prioritized for testing their efficacy in clinical trials.

Our study has several limitations. First, while considering concordant variants makes our findings more easily interpretable, it will miss those SUD-shared variants that are not present in a study due to a variety of reasons (e.g. not passing QC). Second, the sample sizes of both the 1kg-AFR-like and 1kg-AMR-like discovery and target datasets are small to moderate. This resulted in limited statistical power and thus genetic variants with small effects may not be detected. Consequently, the majority of our findings were from the 1kg-EUR-like sample and some of them may not be associated with SUDs in other populations. Additionally, in the 1kg-AMR-like sample, we only have GWAS of AUD and OUD; therefore, our results may be limited to these two SUDs only and not generalizable to other SUDs. Third, due to small GWAS sample sizes in the 1kg-AFR-like and 1kg-AMR-like samples, we cannot accurately estimate SNP-heritability and genetic correlations in these populations. Fourth, real-world data-based analyses were subject to unmeasured confounding and inconsistency between health insurance records and true health statuses.

Although we identified many more variants and observed greater PGS predictive power in the 1kg-EUR-like sample compared to the 1kg-AFR-like and 1kg-AMR-like samples, these differences are primarily due to the smaller sample sizes in the latter groups as well as the lack of appropriate statistical methods for analyzing admixed populations. Importantly, these findings should not be interpreted as evidence for different underlying genetic mechanisms of SUDs across these populations. Rather, our results highlight the critical and urgent need to increase the sample sizes of under-represented populations to reduce health disparities. We also emphasize that genetic factors or PGS do not determine SUD status, and environmental factors also substantially reduce or amplify the effect of genetic factors. Our findings should never be used to discriminate or to stigmatize people, or to deny access to prevention and treatment programs, especially for those from vulnerable populations. Our findings should only be used for research purposes to promote health.

In conclusion, by applying meta-analyses and retaining concordant variants across multiple SUDs, we identified a set of genes shared among different SUDs, providing new insight into their common genetic mechanisms. PGS calculated from these concordant variants had improved predictive power, particularly in the 1kg-EUR-like and the 1kg-AMR-like populations. Finally, we identified seven drugs that target SUD-share genes, highlighting their potential for repurposing in the treatment of SUDs. Together, these findings advance our understanding of the genetic architecture underlying SUDs and point to promising future prevention and treatment strategies.

## Supplementary information

Supplemental figures and tables legends.

Supplemental tables.

Supplemental Figures

## Data Availability

1kg-EUR-like Problematic alcohol use, 1kg-AFR-like alcohol use disorder GWAS (Zhou et al. [23]), and 1kg-EUR-like and 1kg-AFR-like cannabis use disorder GWAS (Levey et al. [24]): https://medicine.yale.edu/lab/gelernter/stats/. 1kg-AMR-like alcohol use disorder GWAS (Zhou et al. [23]) and opioid use disorder GWAS (Kember et al. [25]) are available through dbGaP: https://www.ncbi.nlm.nih.gov/projects/gap/cgi-bin/study.cgi?study_id=phs001672. 1kg-EUR-like and 1kg-AFR-like opioid use disorder GWAS (Polimanti et al. [26]): https://figshare.com/articles/dataset/sud2020-op/14672211. 1kg-EUR-like and 1kg-AFR-like tobacco use disorder GWAS (Toikumo et al. [27]) are available by emailing the corresponding author. FinnGen Substance abuse: https://www.finngen.fi/en/access_results. The Human Brain Cell Atlas v1.0 generated by BRAIN Initiative cell census network: https://cellxgene.cziscience.com/collections/283d65eb-dd53-496d-adb7-7570c7caa443. All of Us research program: https://www.researchallofus.org/data-tools/workbench/. Indiana Biobank: https://indianabiobank.org/. Indiana biobank substance use disorder cohort is also available from dbGaP: https://www.ncbi.nlm.nih.gov/projects/gap/cgi-bin/study.cgi?study_id=phs003025.v1.p1. All GWAS summary statistics from this study will be deposited to GWAS catalog: https://www.ebi.ac.uk/gwas/. All variants and their weights used to calculate PGS will be available from PGS catalog: https://www.pgscatalog.org/.
