# Supplementary material for: Genome-wide meta-analyses of cross substance use disorders in diverse populations
Source: Mol Psychiatry. 2025 Oct 7;31(3):1619–33. doi: 10.1038/s41380-025-03294-5 (PMC12916498; doi:10.1038/s41380-025-03294-5)
Supplement: Supplementary file 1 — Supplemental figures and tables legends. [file 41380_2025_3294_MOESM1_ESM.docx]

**Supplemental Figure 1**. LocusZoom plots of each locus in different populations. Note: Only concordant variants were plotted.

**Supplemental Figure 2**: Manhattan plots of gene-based analyses. A: 1kg-EUR-like; B: 1kg-AFR-like; C: 1kg-AMR-like; D: meta-analysis of 1kg-AFR-like and 1kg-EUR-like; E: meta-analysis of 1kg-EUR-like and 1kg-AMR-like; F: meta-analysis of 1kg-AFR-like, 1kg-EUR-like, and 1kg-AMR-like.

Supplemental Table 1 Genetic correlations among different SUDs in 1kg-EUR-like

Supplemental Table 2 Genome-wide significant variants in 1kg-EUR-like

Supplemental Table 3 Genome-wide significant variants in 1kg-AFR-like

Supplemental Table 4 Genome-wide significant variants in 1kg-AMR-like

Supplemental Table 5 Genome-wide significant variants in 1kg-AFR+EUR-like

Supplemental Table 6 Genome-wide significant variants in 1kg-AMR+EUR-like

Supplemental Table 7 Genome-wide significant variants in 1kg-AFR+AMR+EUR-like

Supplemental Table 8 1kg-EUR-like gene-based results

Supplemental Table 9 1kg-AFR-like gene-based results

Supplemental Table 10 1kg-AMR-like gene-based results

Supplemental Table 11 1kg-AFR+EUR-like gene-based results

Supplemental Table 12 1kg-AMR+EUR-like gene-based results

Supplemental Table 13 1kg-AFR+AMR+EUR-like gene-based results

Supplemental Table 14 Mapped genes in 1kg-EUR-like

Supplemental Table 15 Mapped genes in 1kg-AMR-like

Supplemental Table 16 Mapped genes in 1kg-AFR+EUR-like

Supplemental Table 17 Mapped genes in 1kg-AMR+EUR-like

Supplemental Table 18 Mapped genes in 1kg-AFR+AMR+EUR-like

Supplemental Table 19 Prioritized genes

Supplemental Table 20 Enrichment analysis results of highly expressed genes in brain dissections

Supplemental Table 21 Enrichment analysis results of lowly expressed genes in brain dissections

Supplemental Table 22 Enrichment analysis results of highly expressed genes in brain cell types

Supplemental Table 23 Enrichment analysis results of lowly expressed genes in brain cell types

Supplemental Table 24 SNP-heritability estimation and SNP-heritability explained

Supplemental Table 25 Genetic correlations with other traits in 1kg-EUR-like

Supplemental Table 26 PGS analysis results

Supplemental Table 27 Drugs and their target genes identified

Supplemental Table 28 Description of Clinformatics data
