# Supplementary material for: Genome-wide meta-analyses of cross substance use disorders in diverse populations
Source: Mol Psychiatry. 2025 Oct 7;31(3):1619–33. doi: 10.1038/s41380-025-03294-5 (PMC12916498; doi:10.1038/s41380-025-03294-5)
Supplement: Supplementary file 3 — Supplemental Figures [file 41380_2025_3294_MOESM3_ESM.docx]

**Supplemental Figure 1**. LocusZoom plots of each locus in different populations. Note: Only concordant variants were plotted.

**Supplemental Figure 2**: Manhattan plots of gene-based analyses. A: 1kg-EUR-like; B: 1kg-AFR-like; C: 1kg-AMR-like; D: meta-analysis of 1kg-AFR-like and 1kg-EUR-like; E: meta-analysis of 1kg-EUR-like and 1kg-AMR-like; F: meta-analysis of 1kg-AFR-like, 1kg-EUR-like, and 1kg-AMR-like.

**Supplemental Figure 1**. LocusZoom plots of each locus in different populations. Note: Only concordant variants were plotted.

Locus 1:
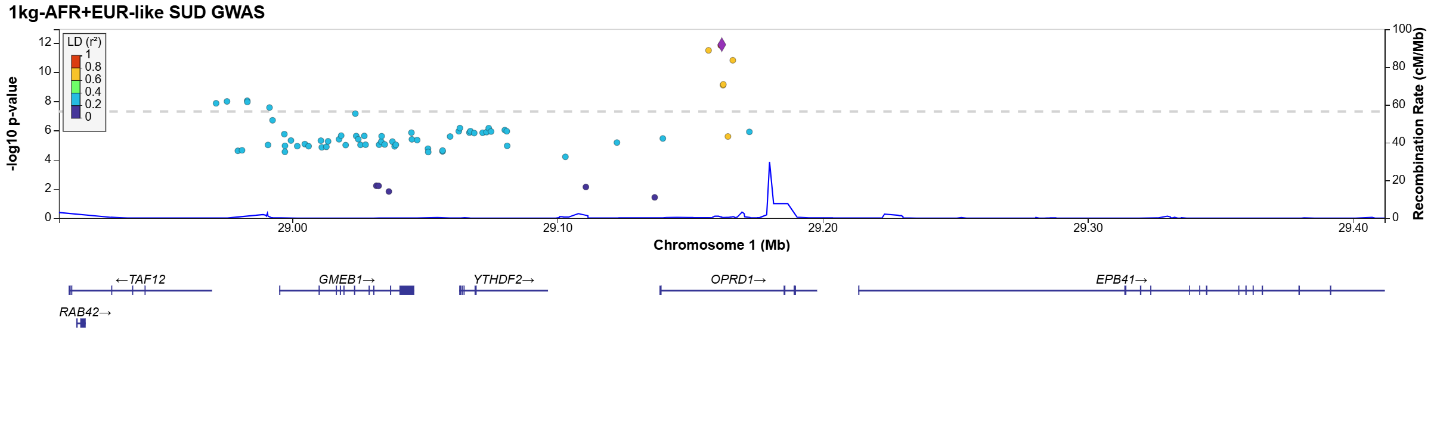

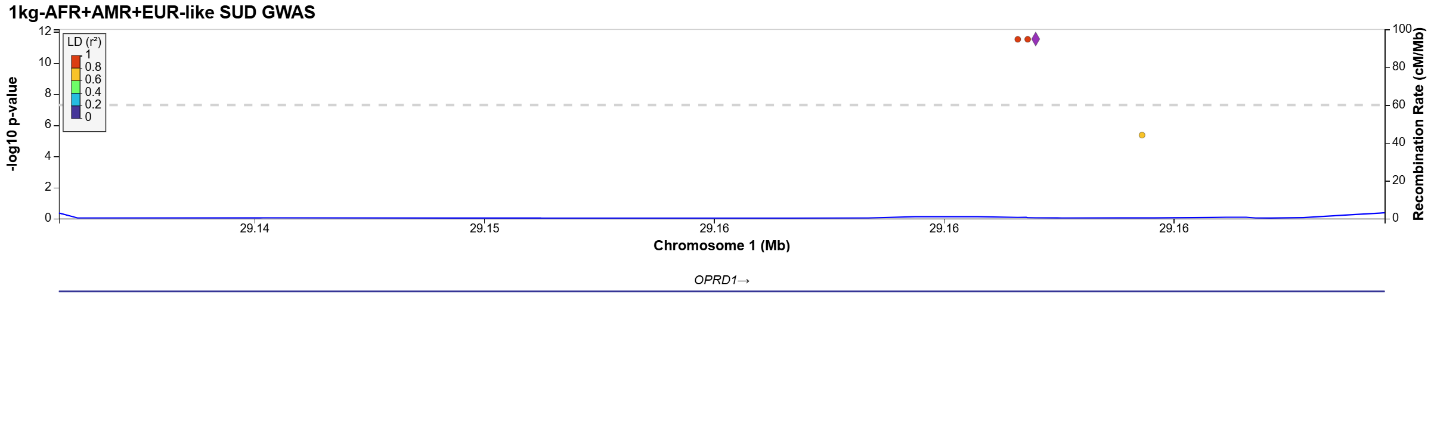

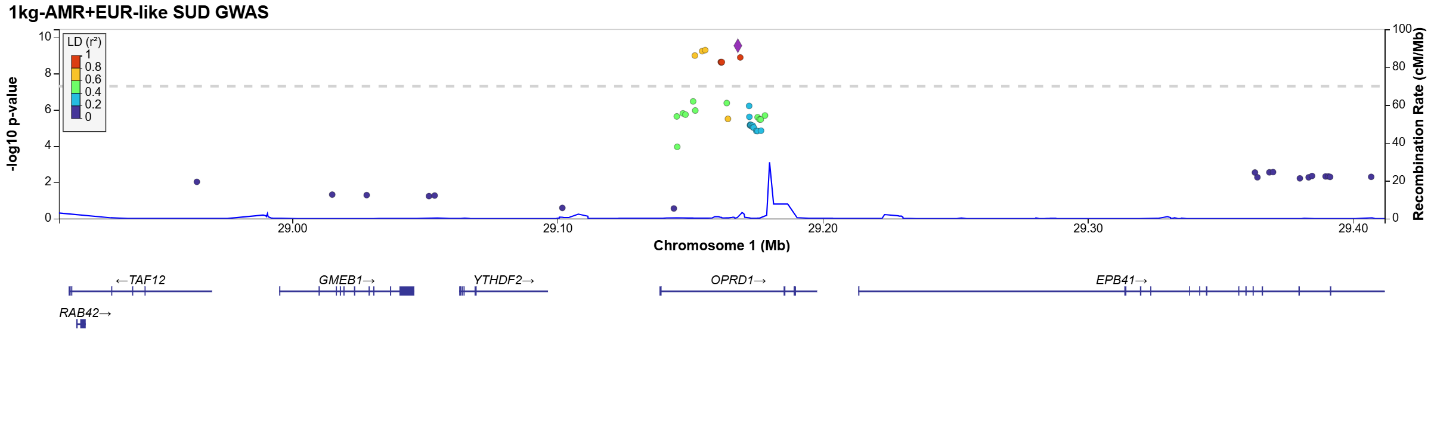


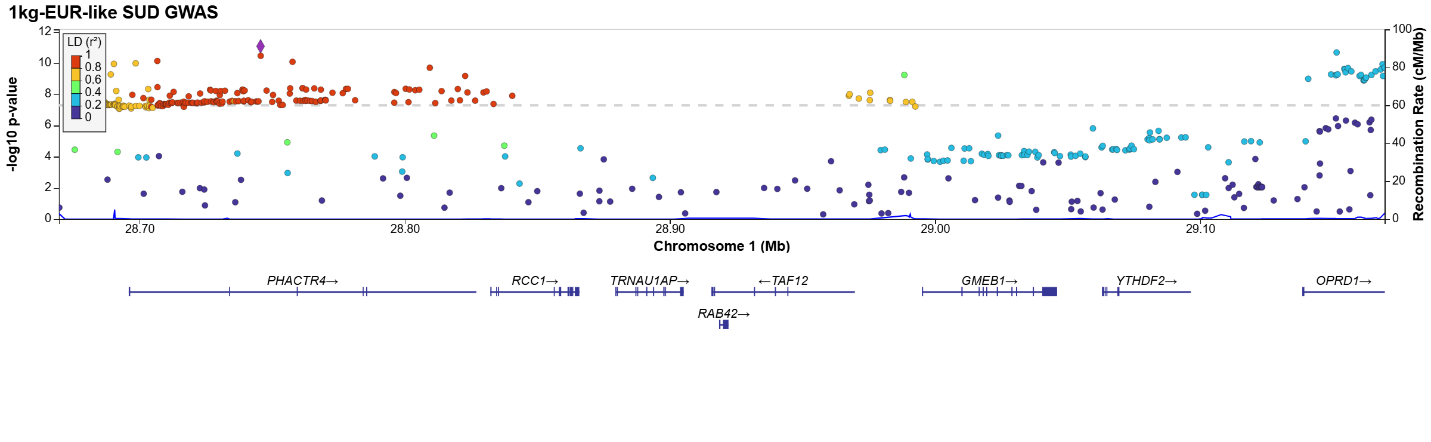


Locus 2:


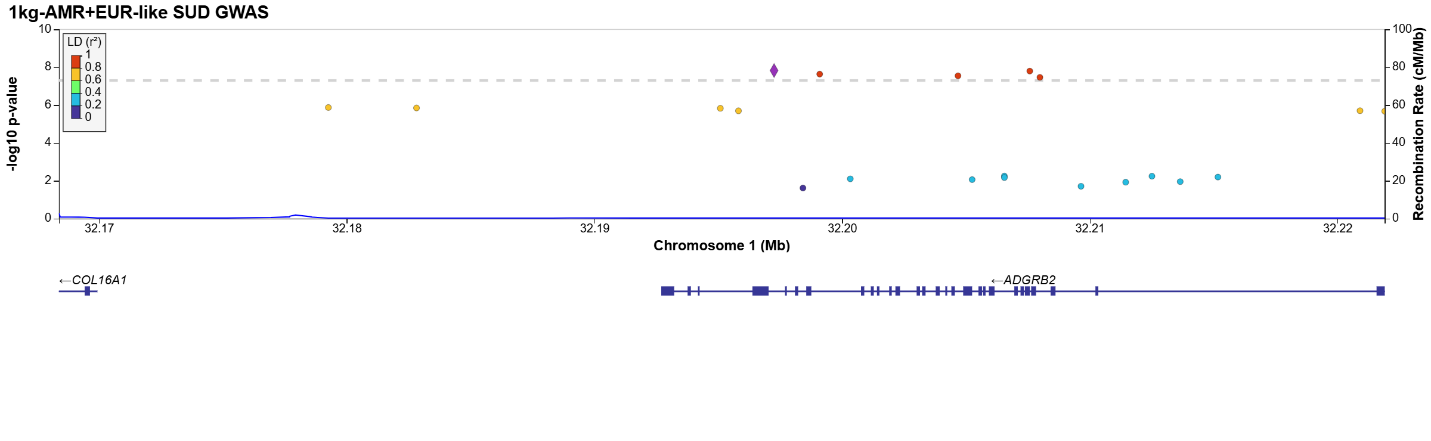


Locus 3:


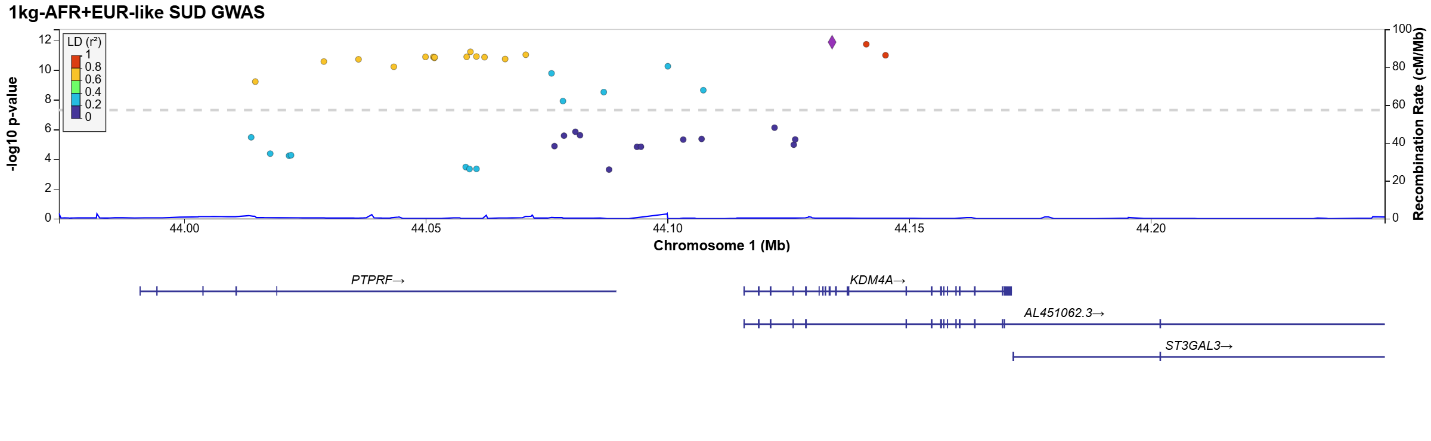


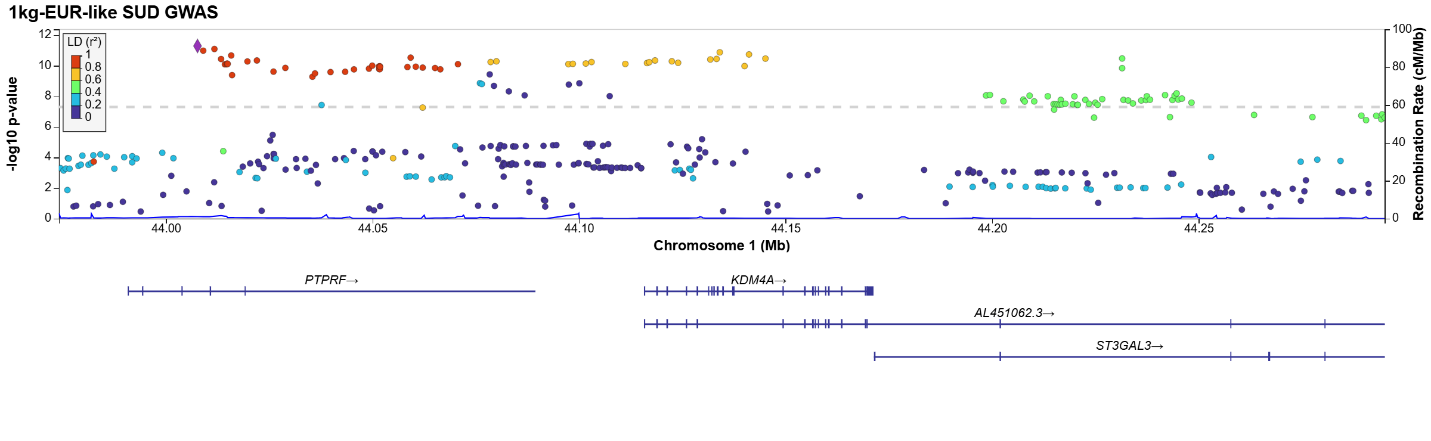

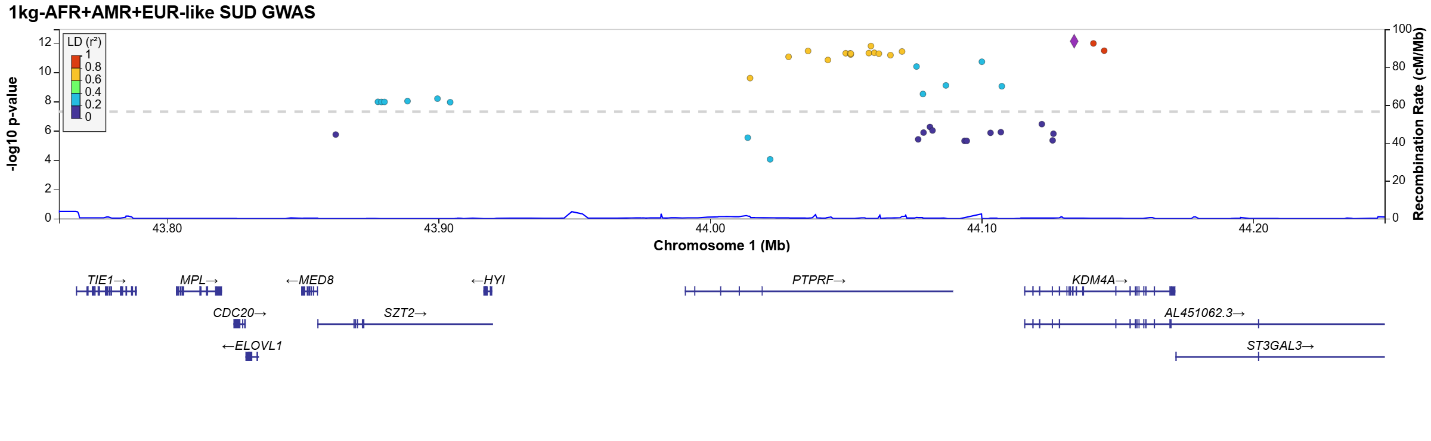


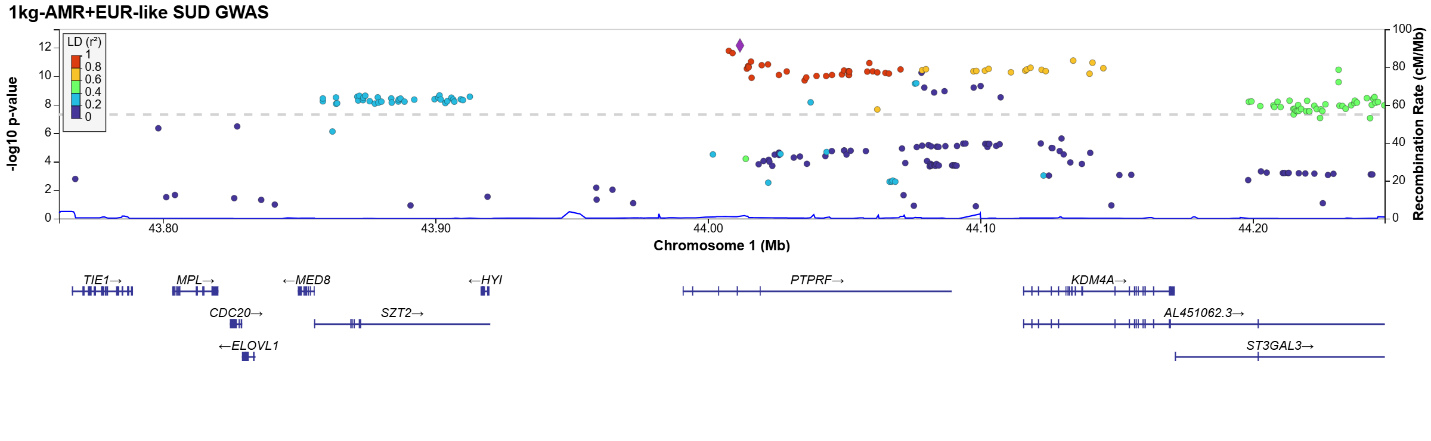


Locus 4:


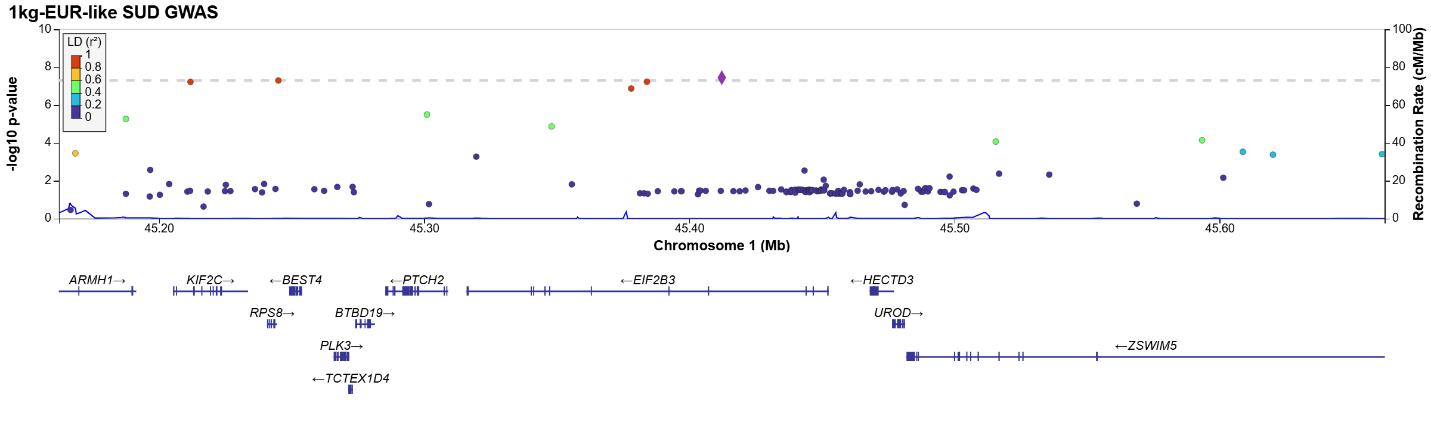


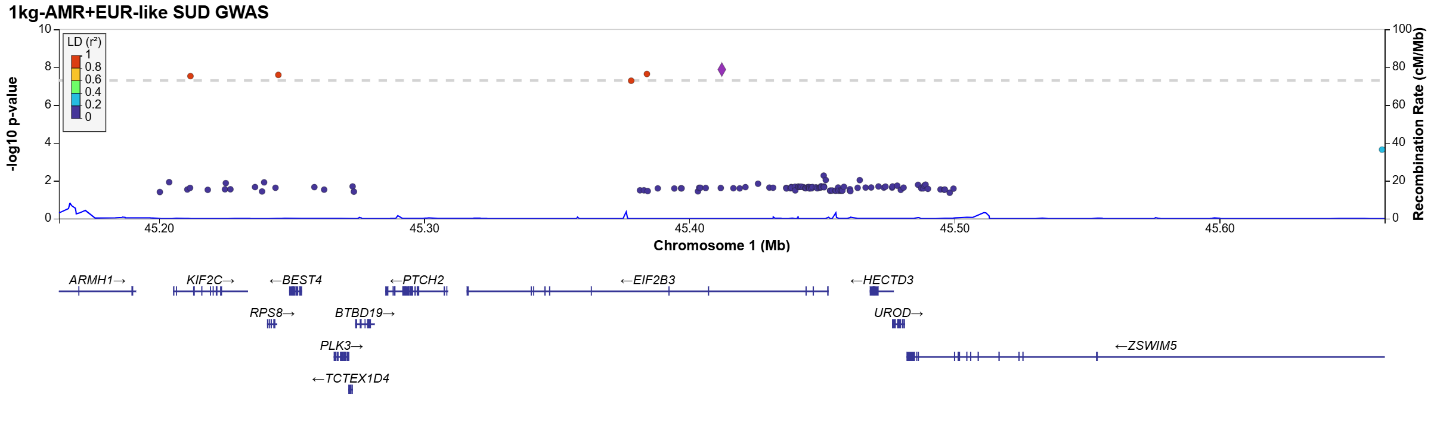


Locus 5:


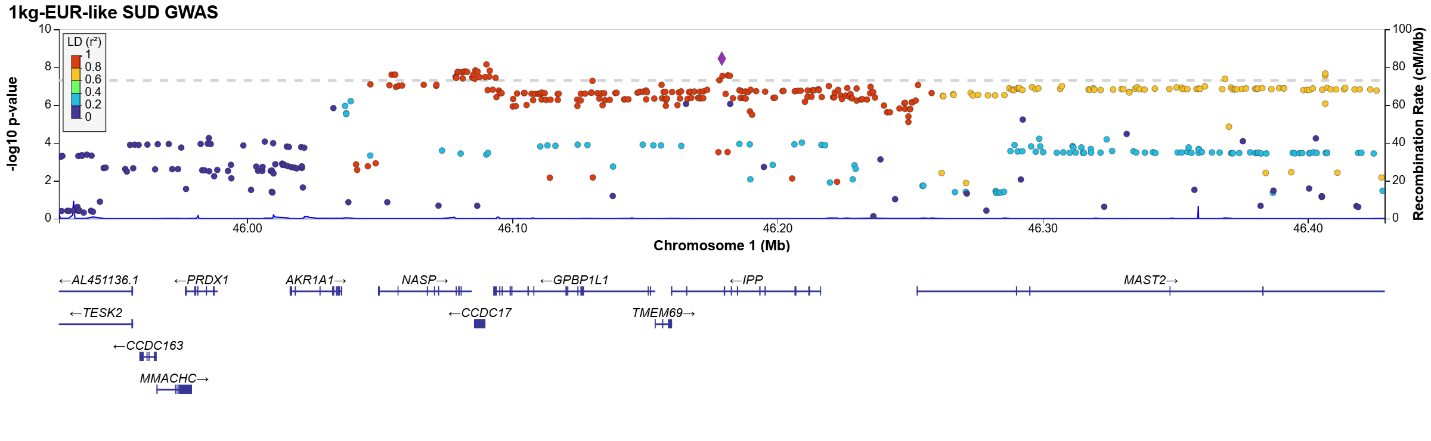

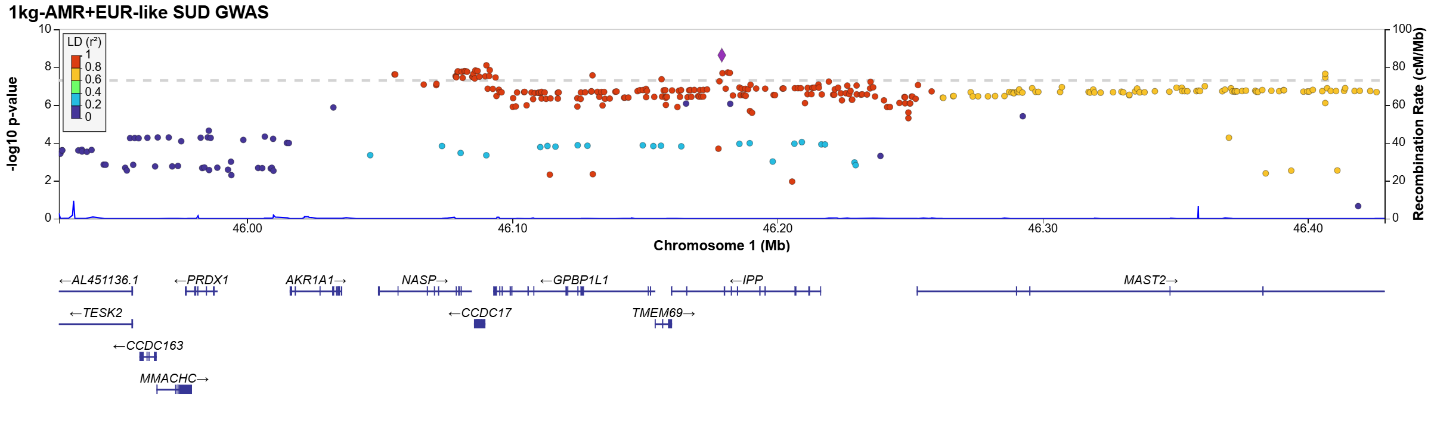

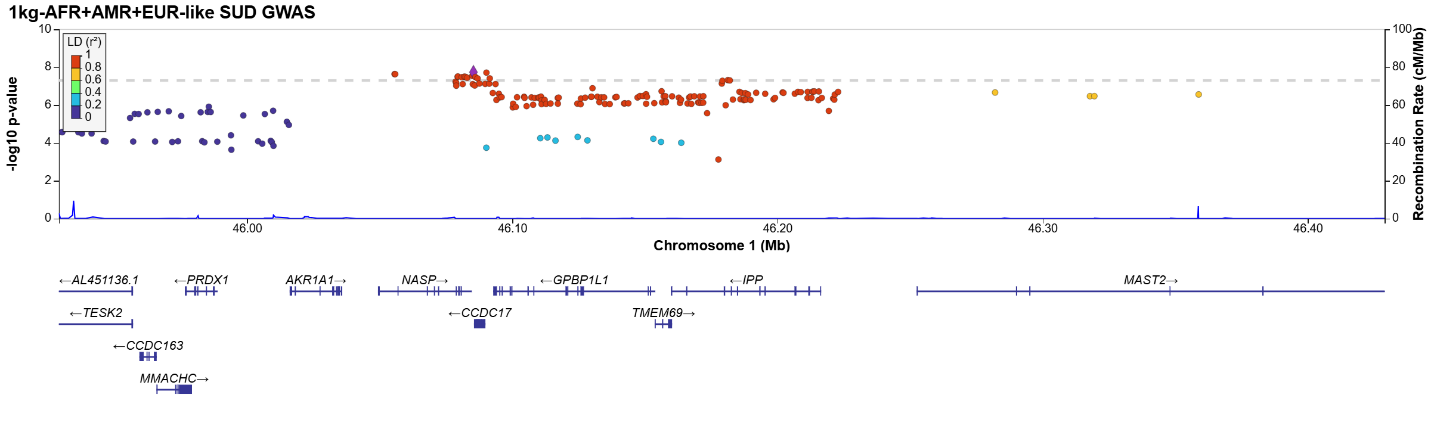

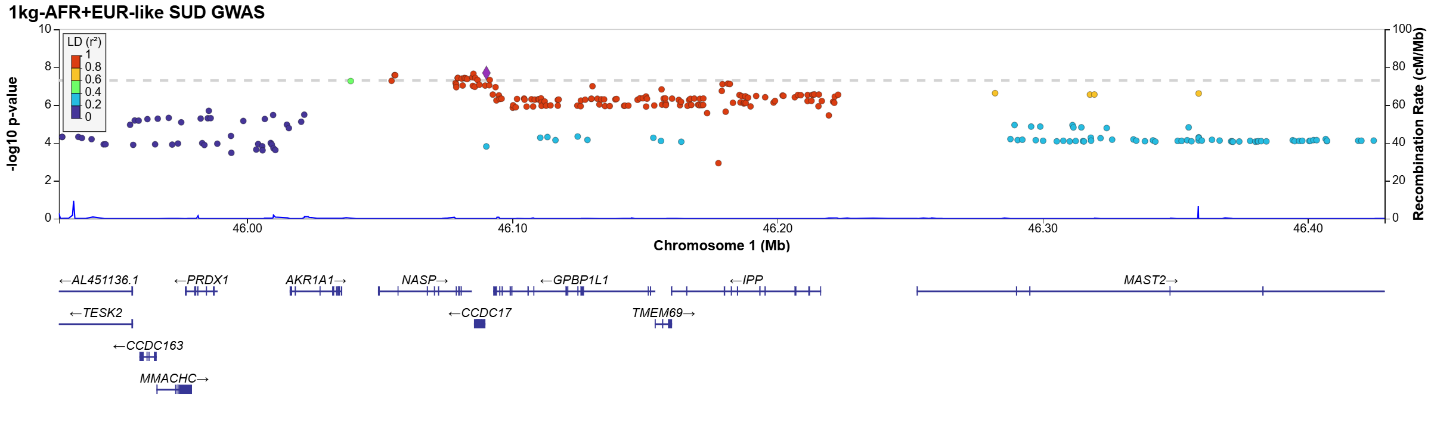


Locus 6:


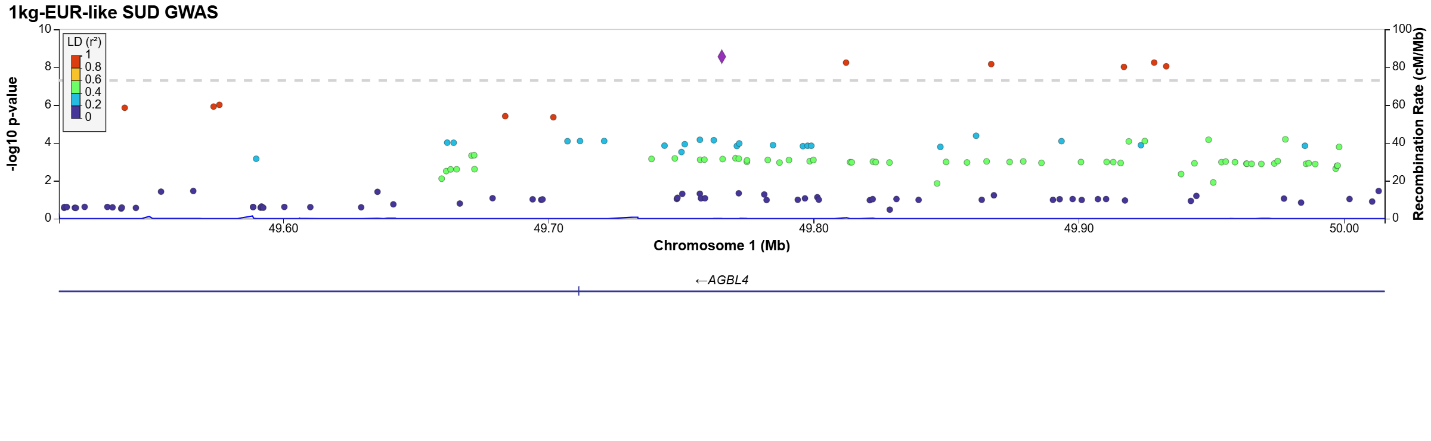


Locus 7:


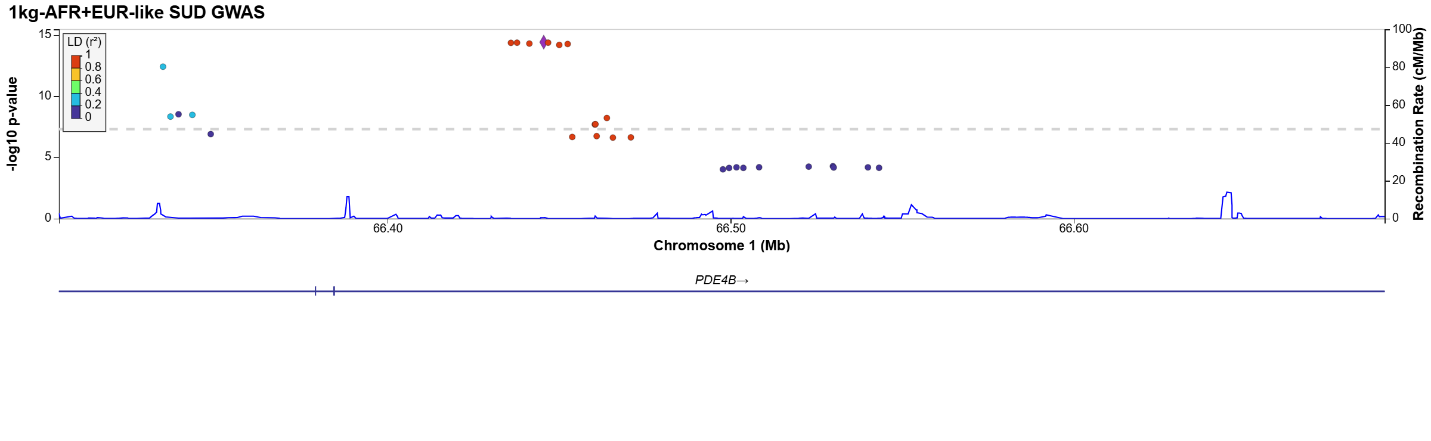


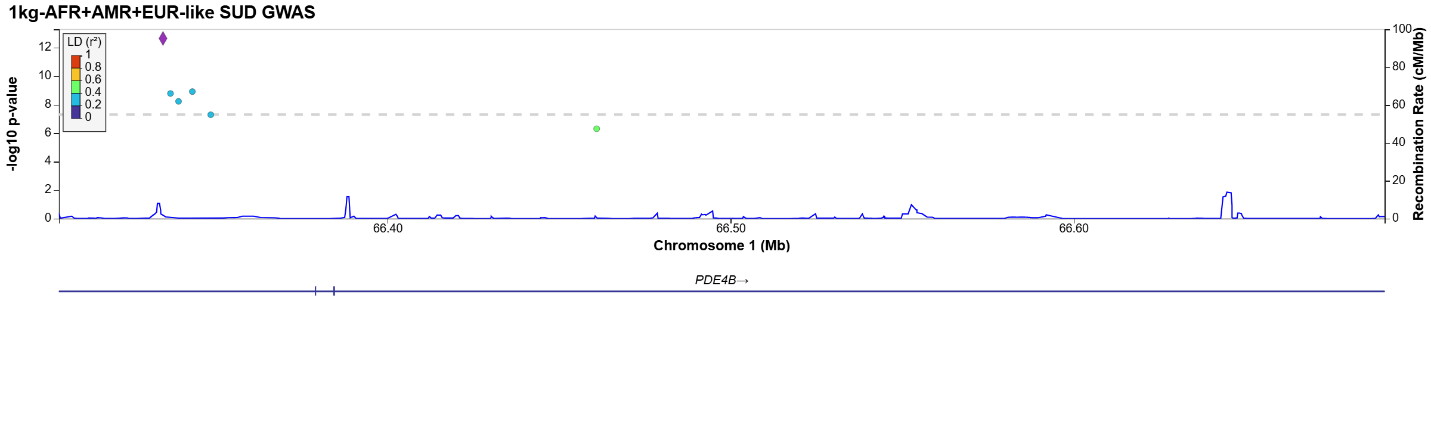


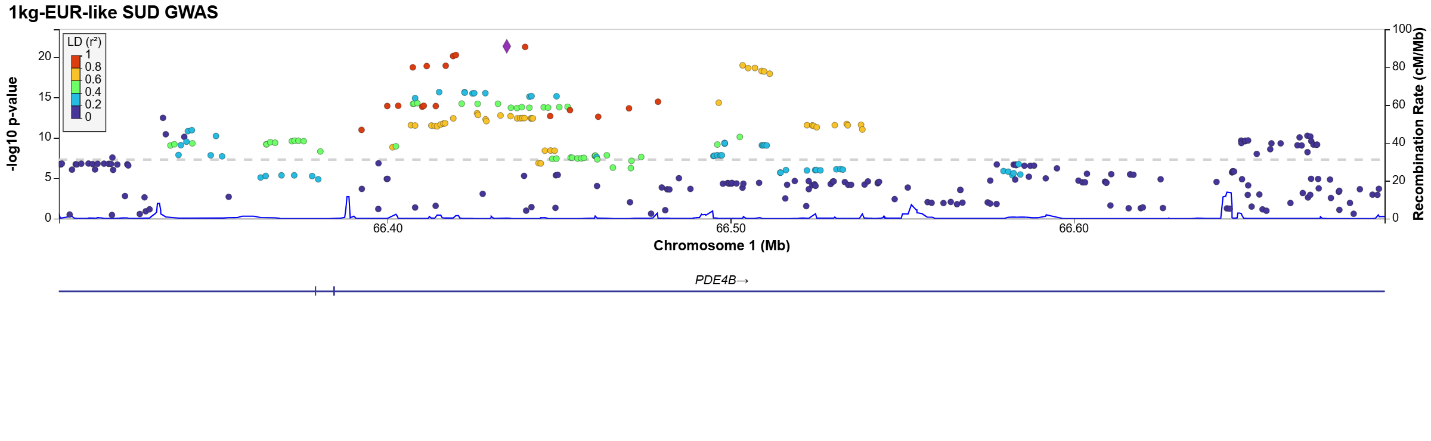


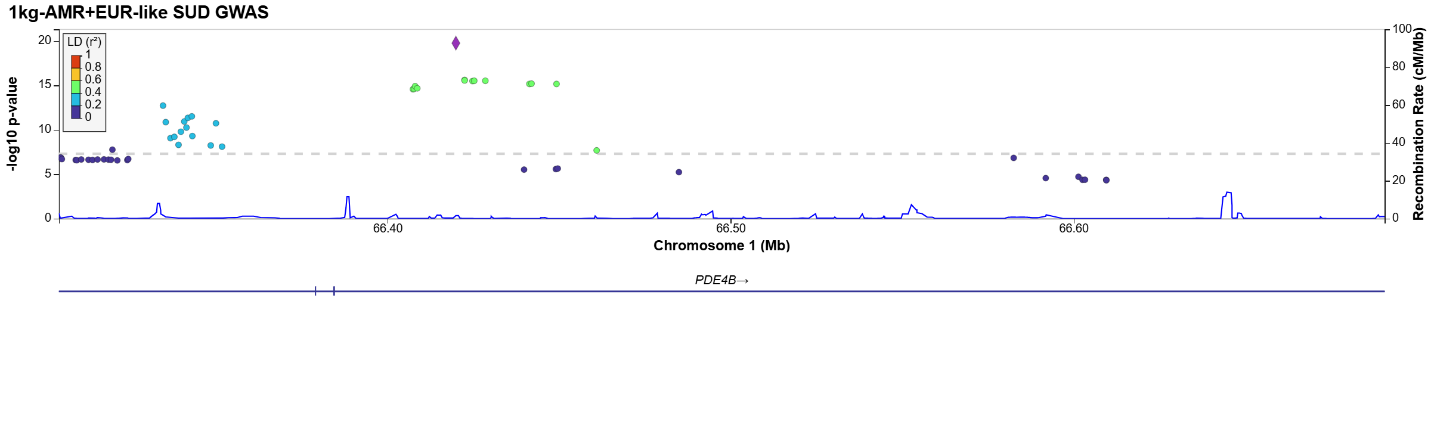


Locus 8:


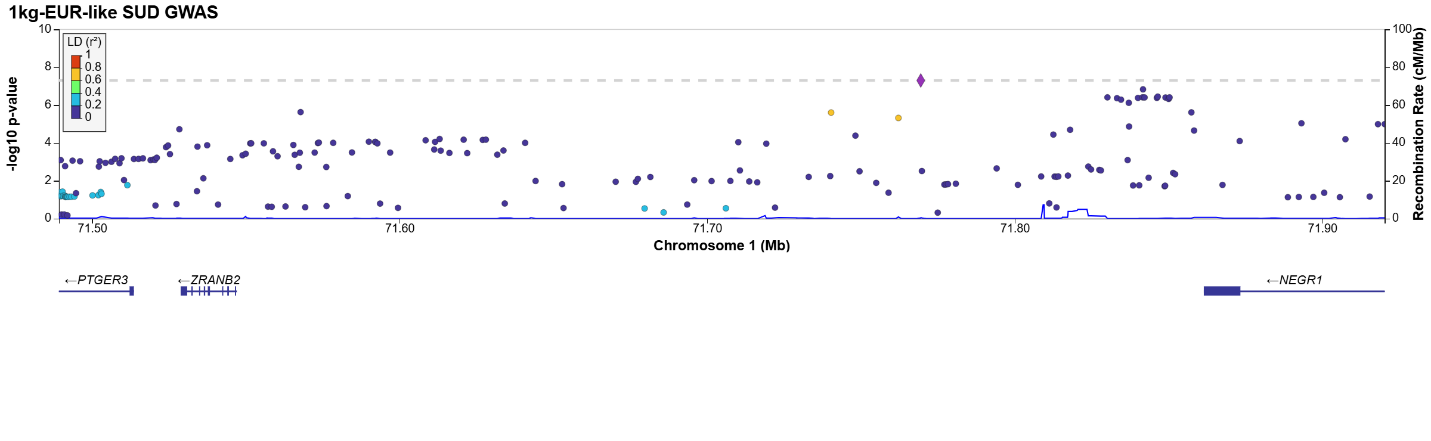


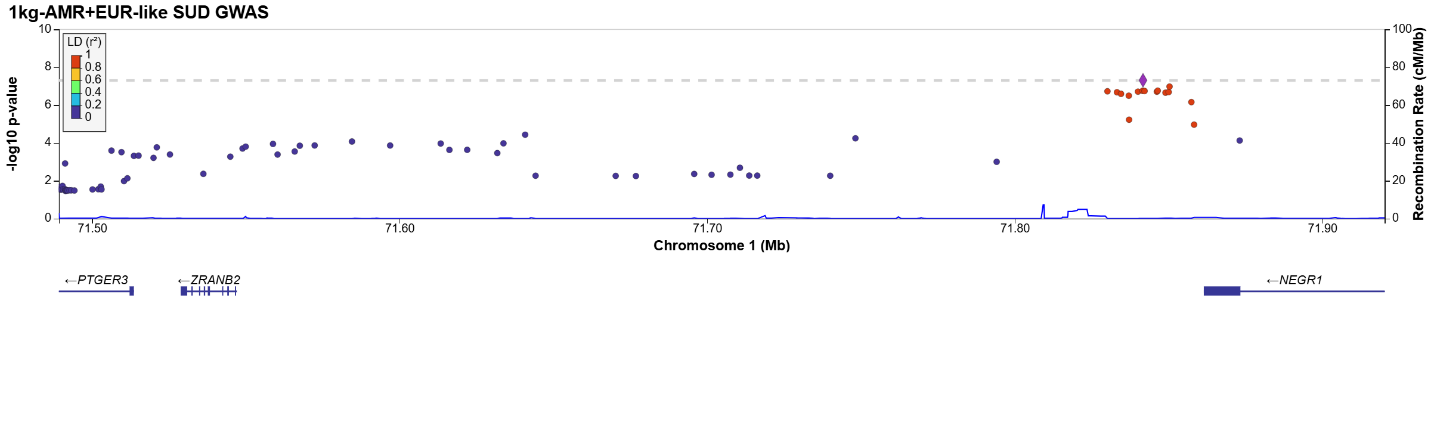


Locus 9:


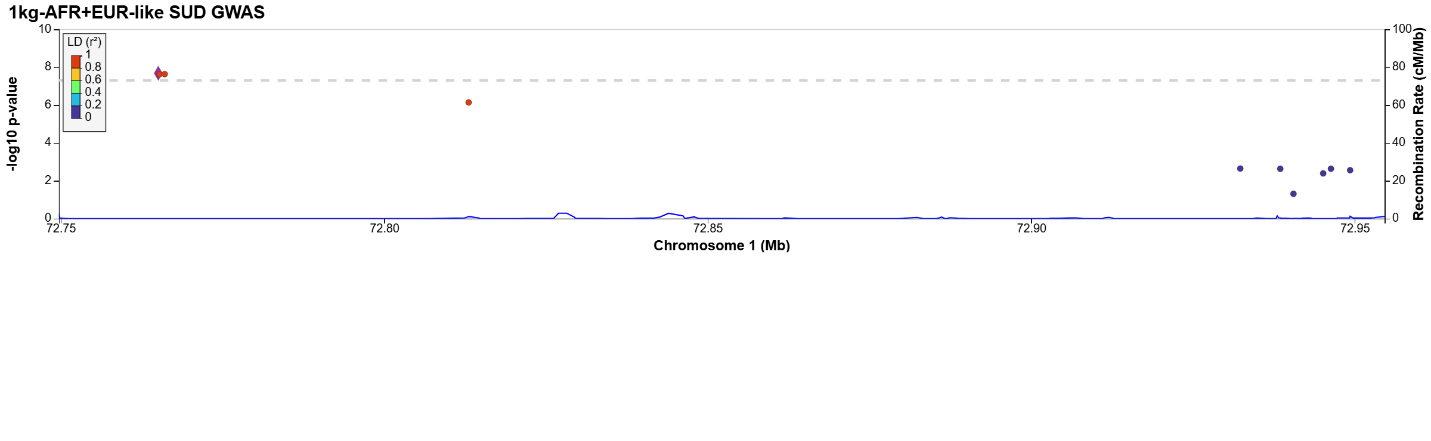


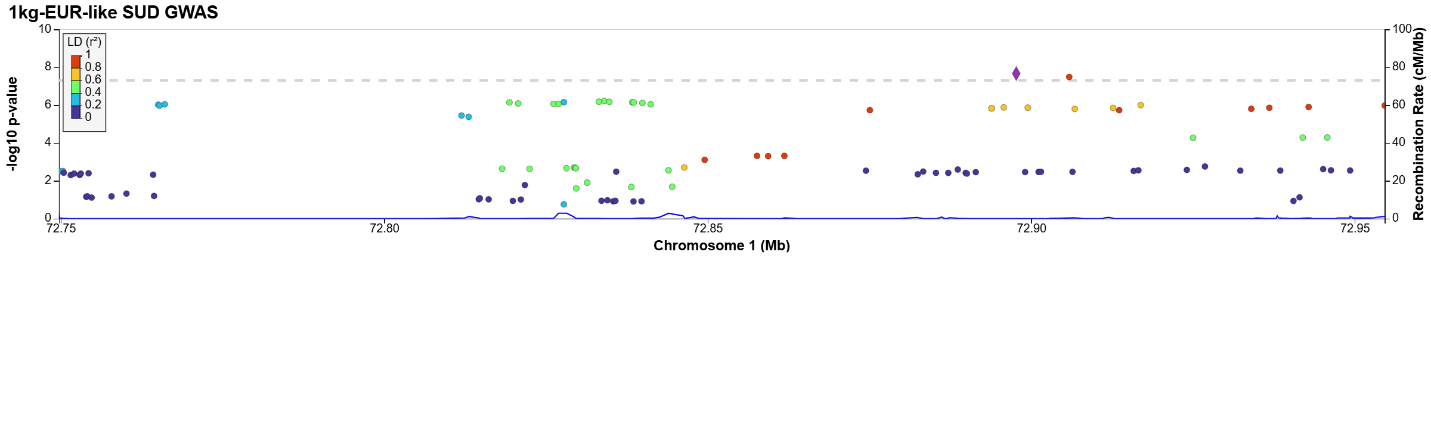


Locus 10:


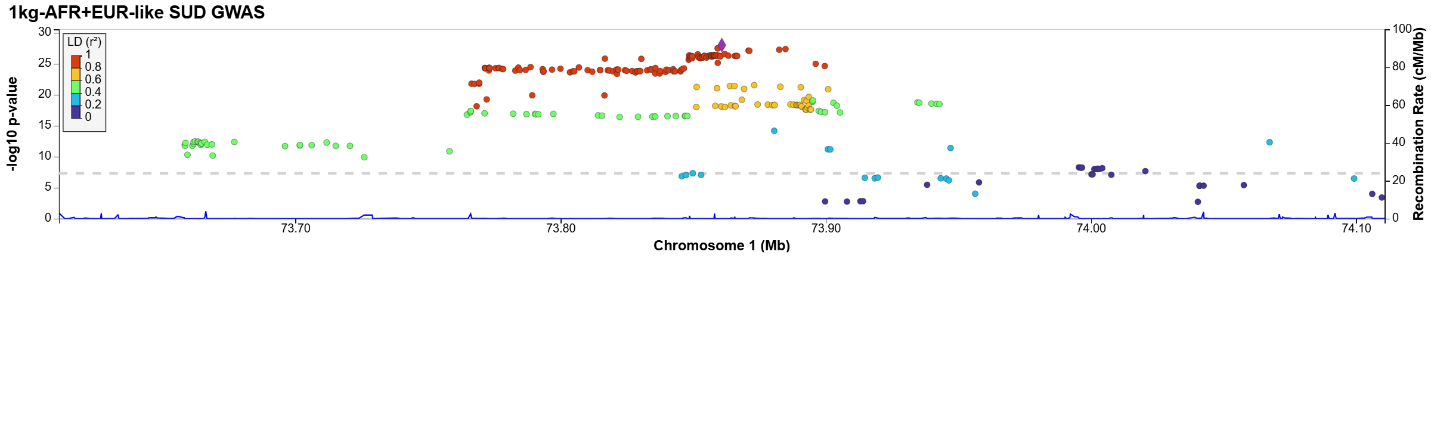


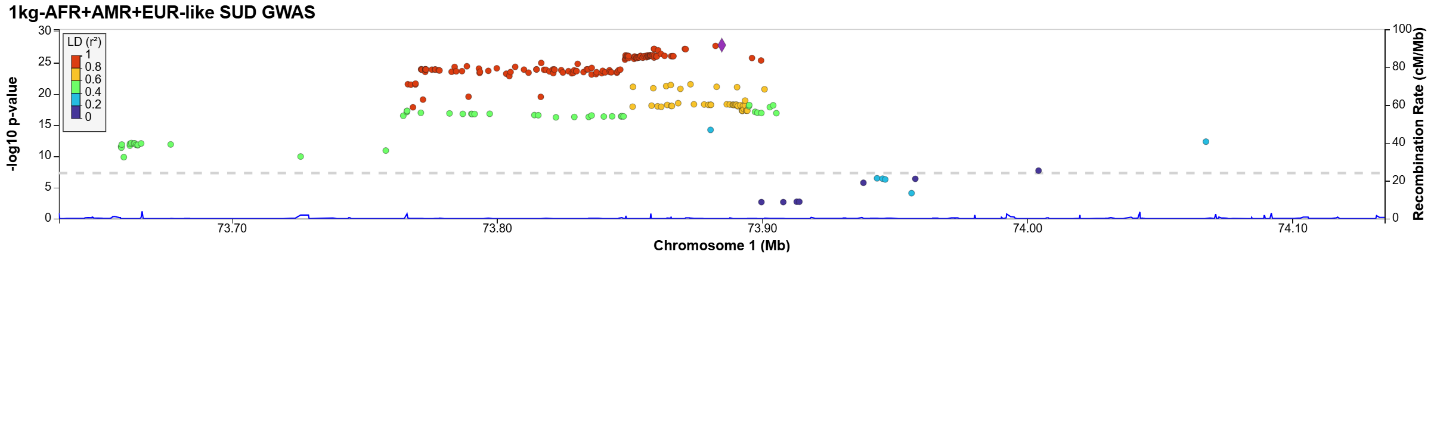


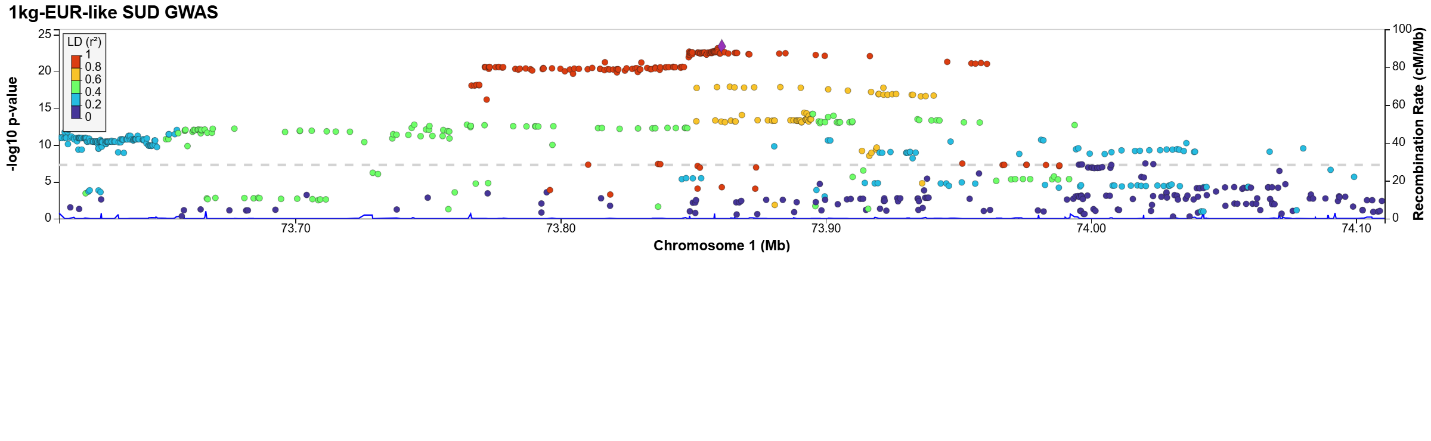


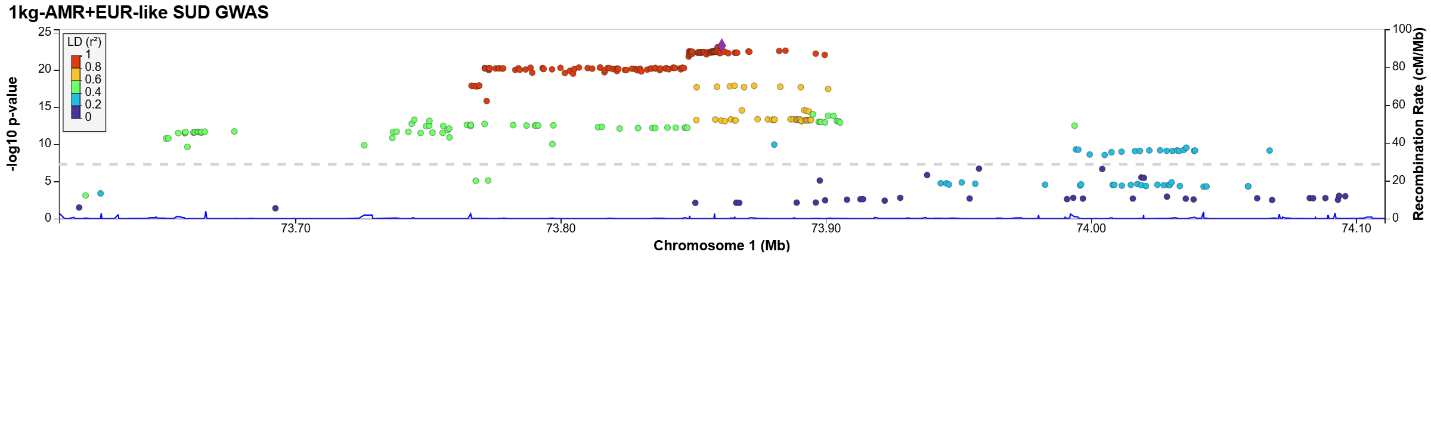


Locus 11:


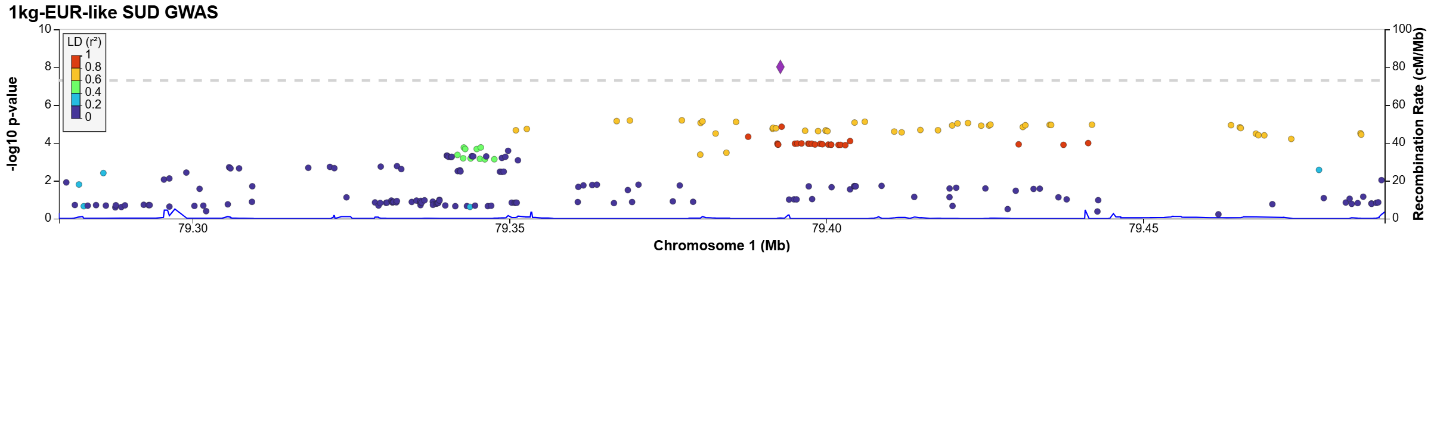


Locus 12:


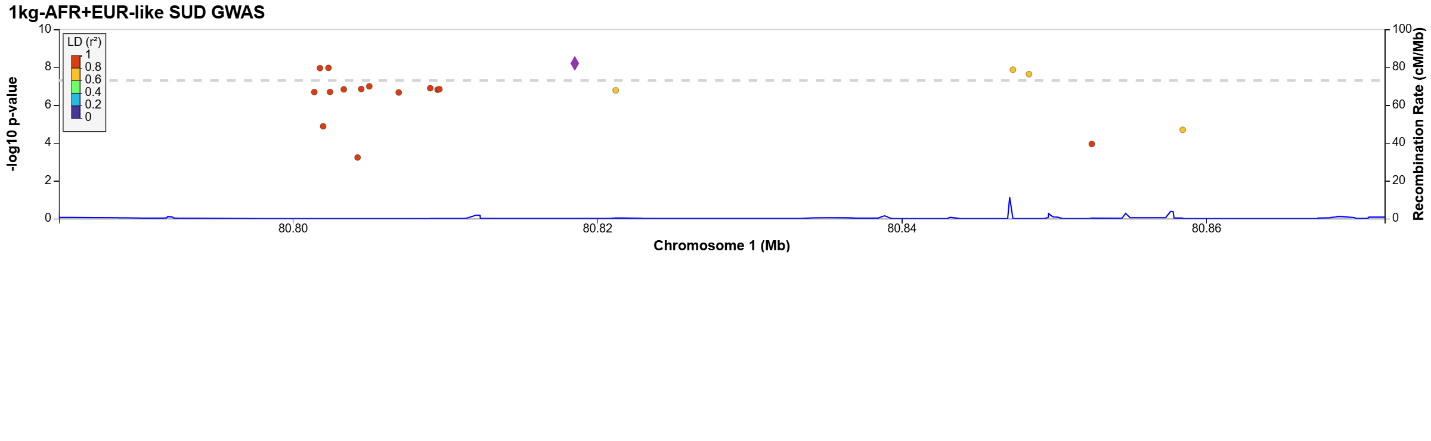


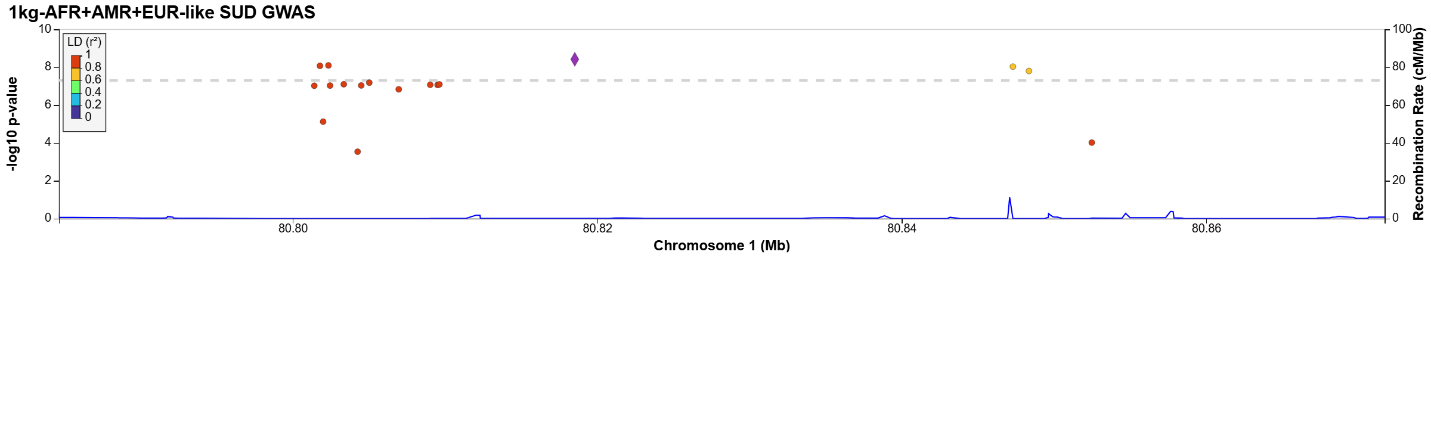


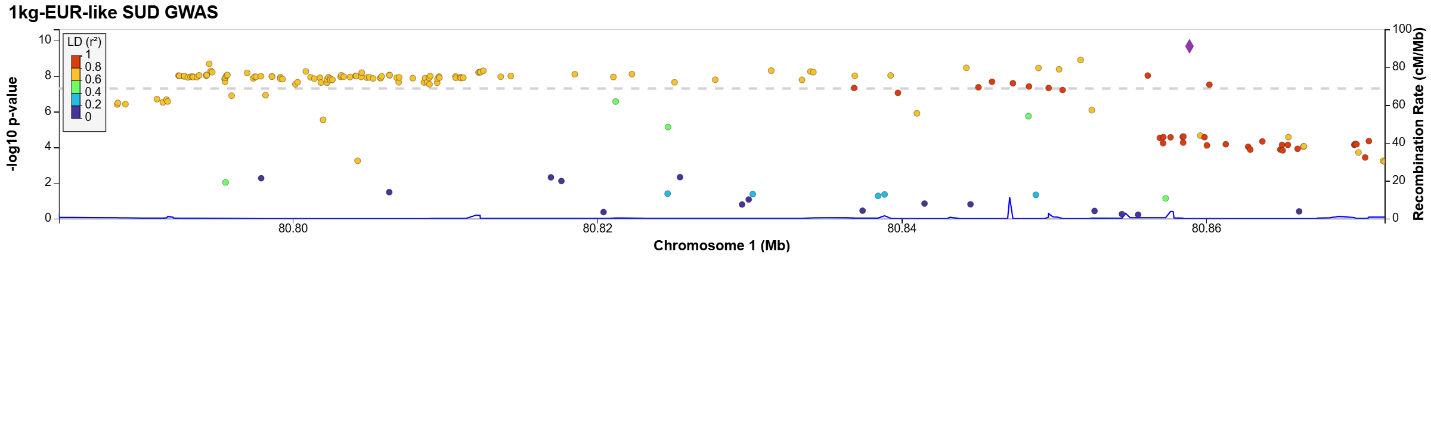


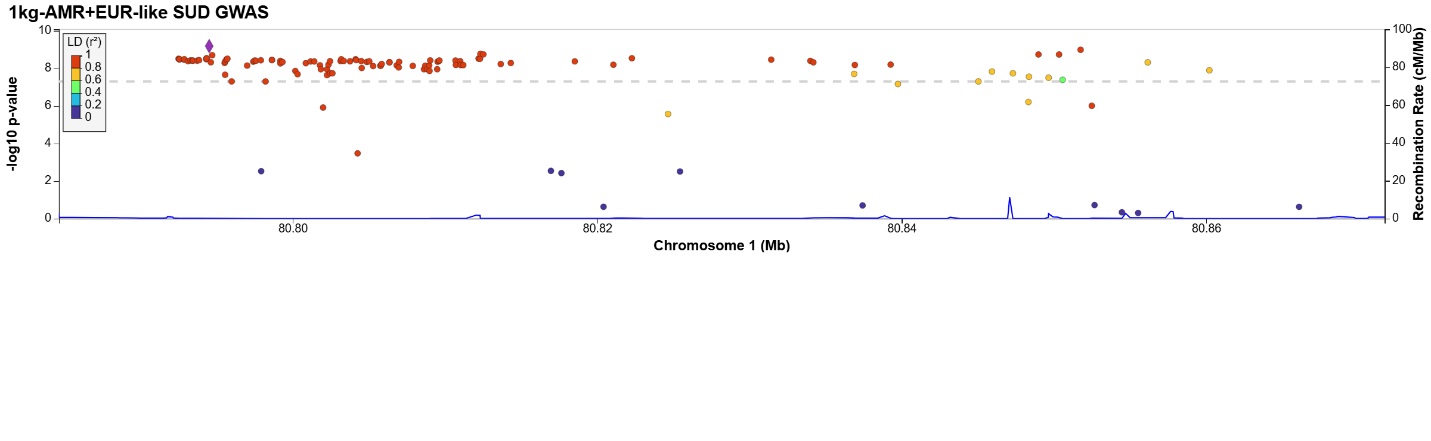


Locus 13:


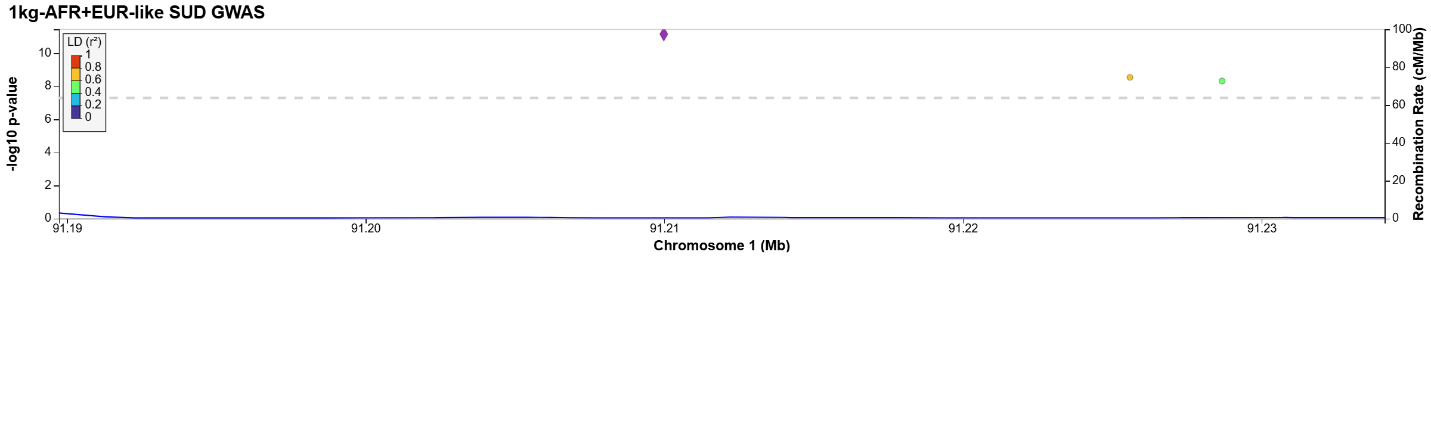


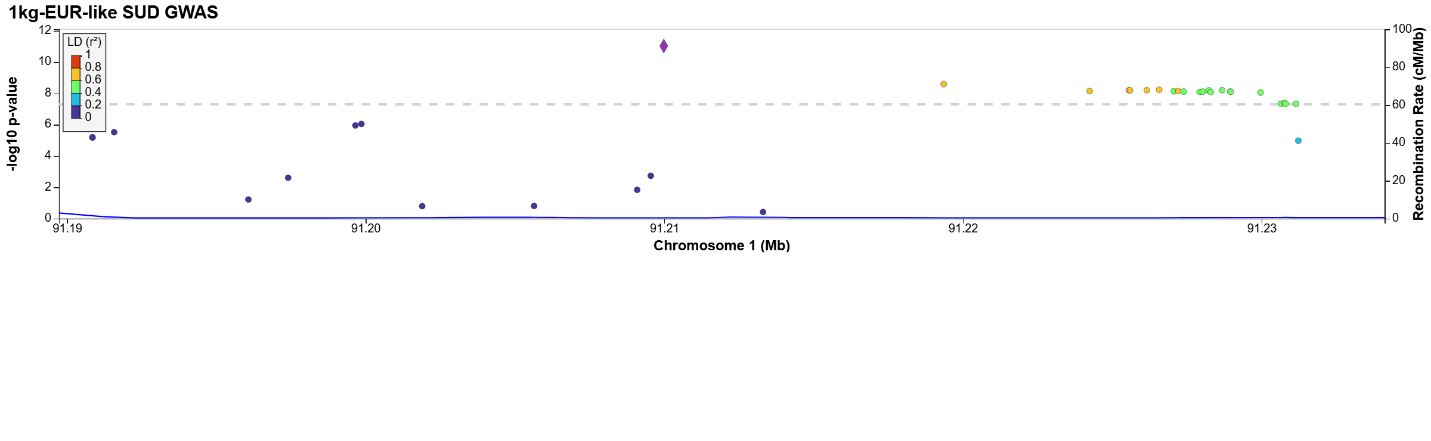


Locus 14:


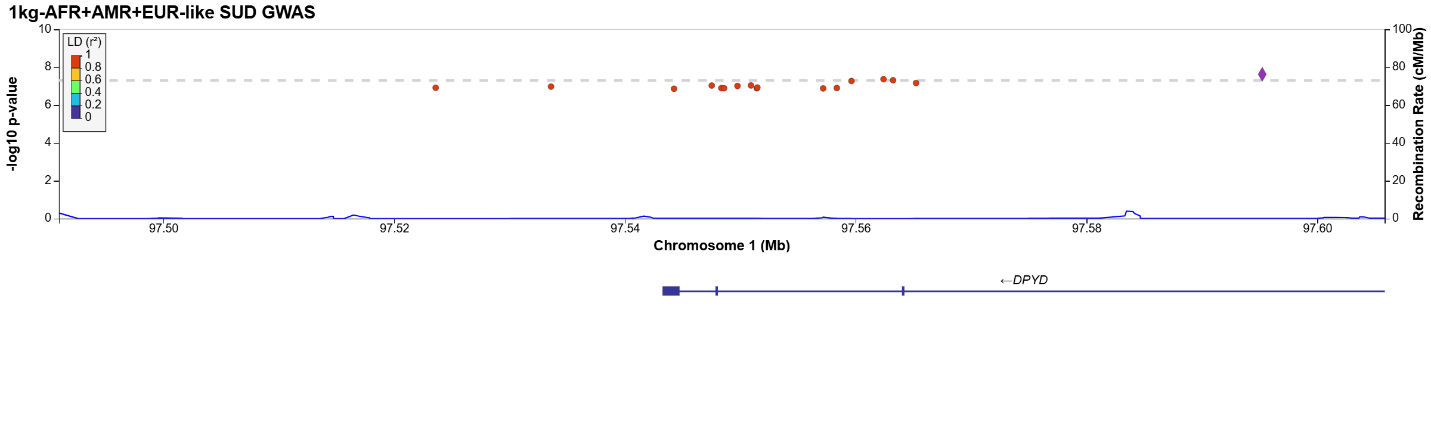


Locus 15:


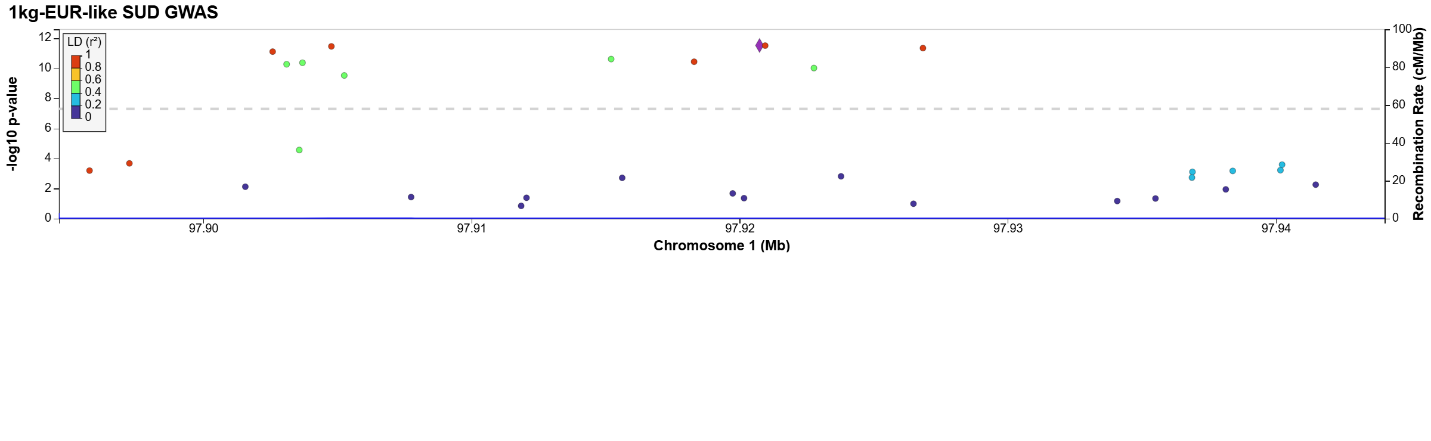


Locus 16:


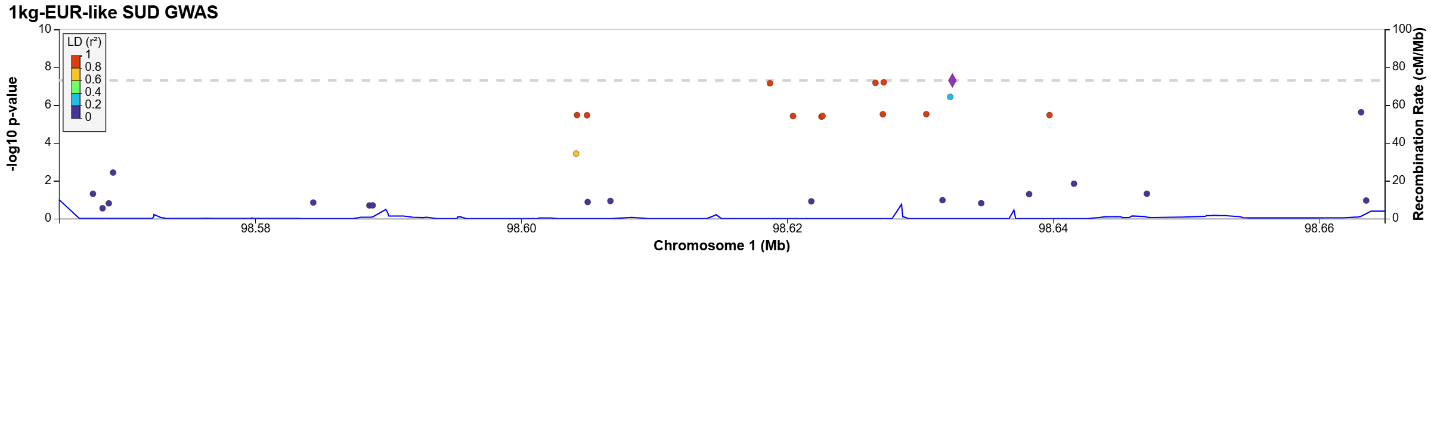


Locus 17:


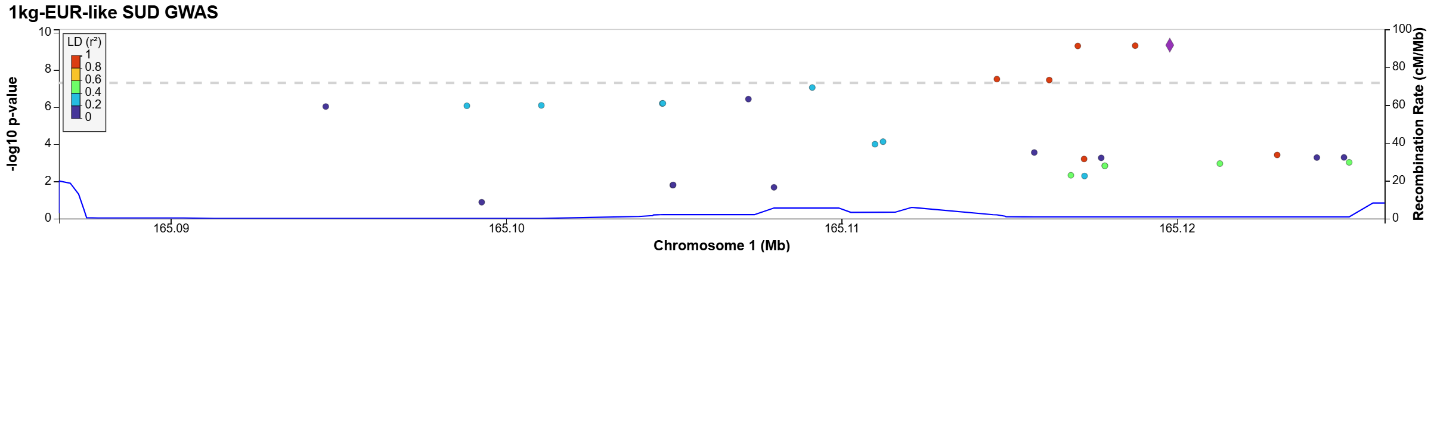


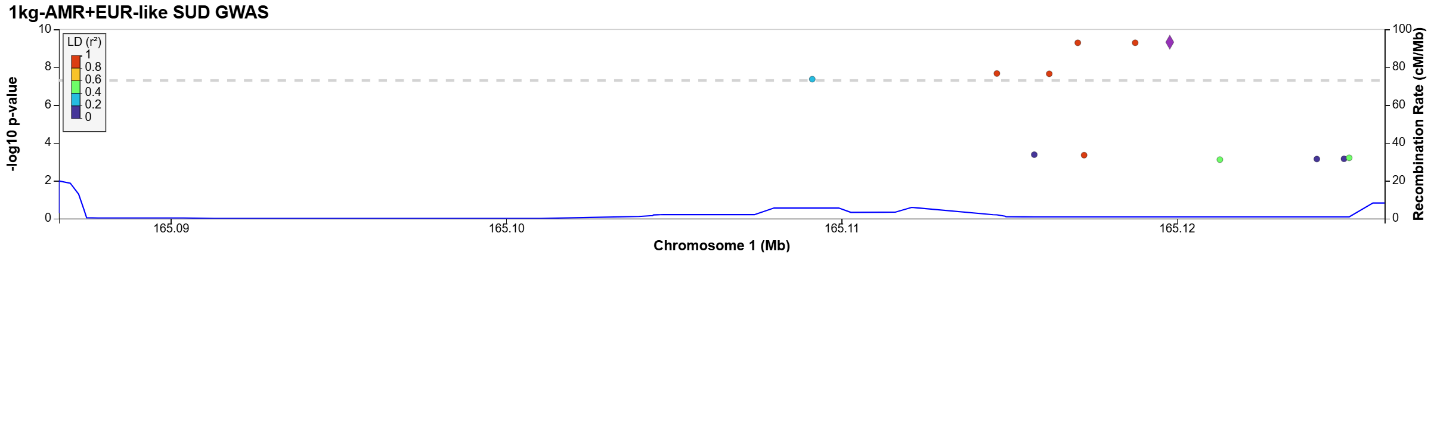


Locus 18:


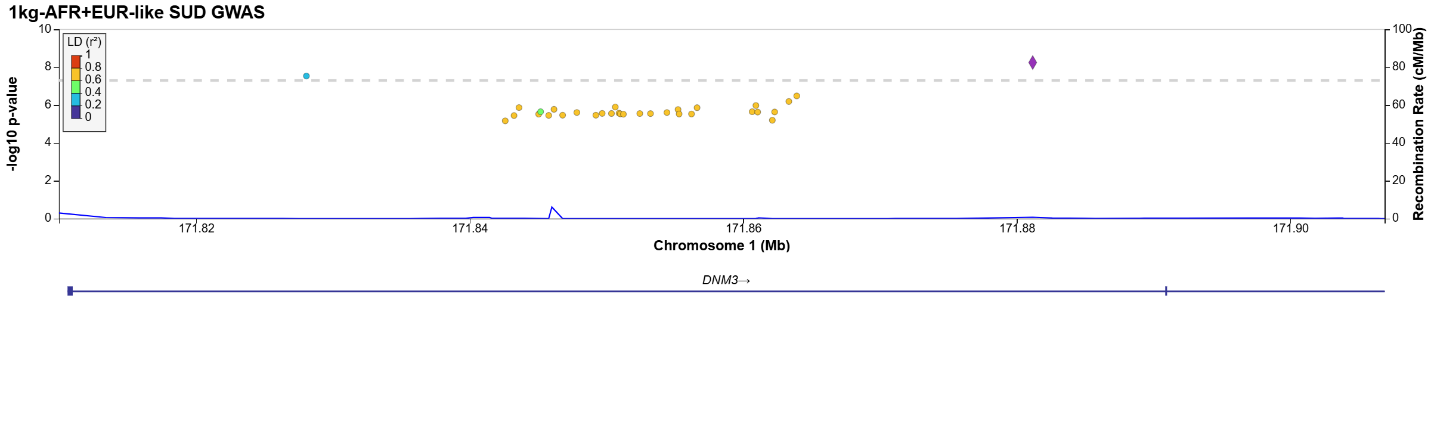


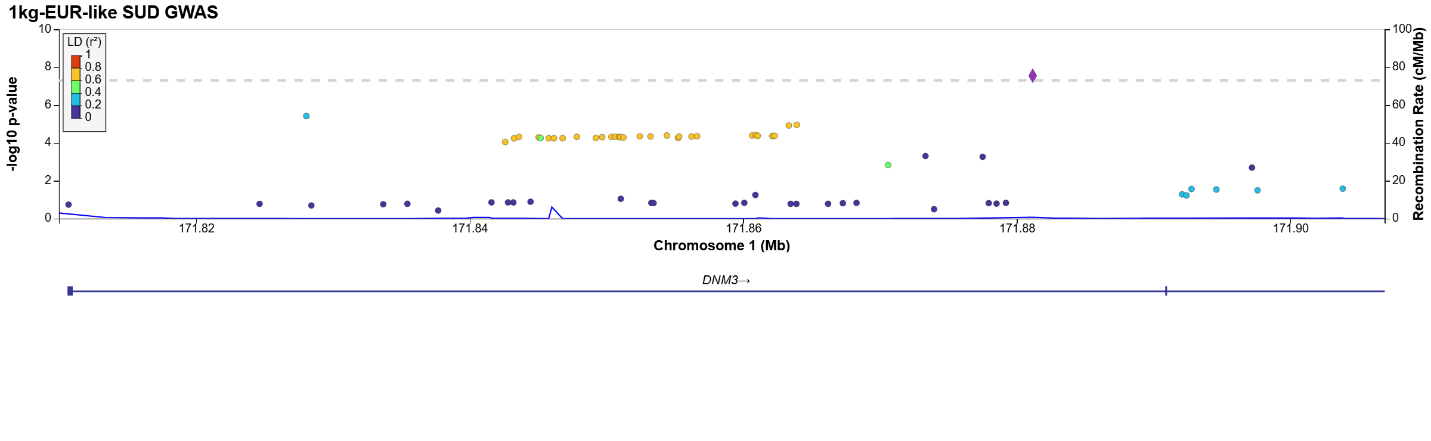


Locus 19
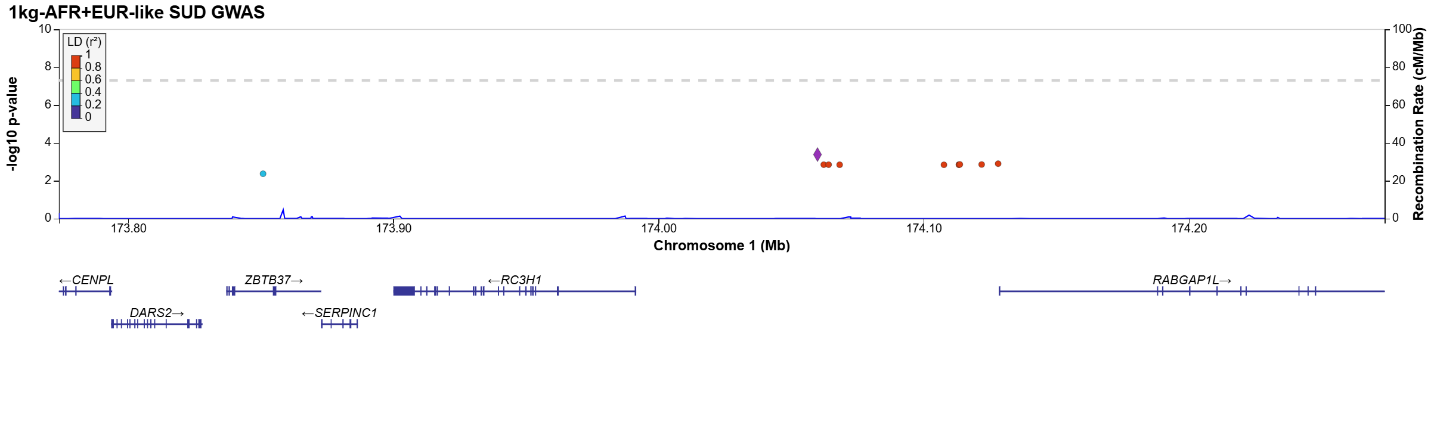


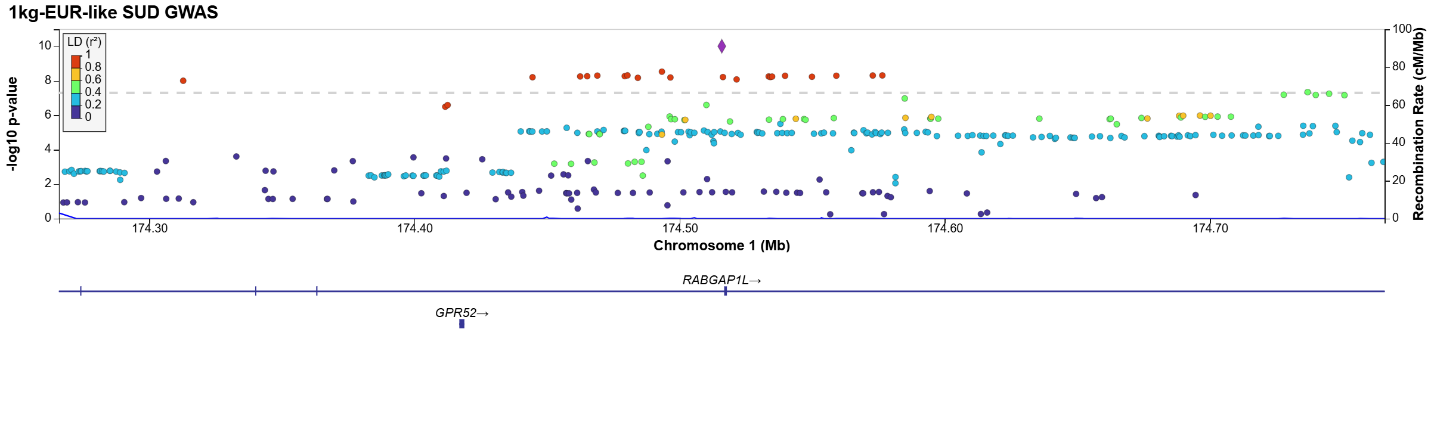


Locus 20:


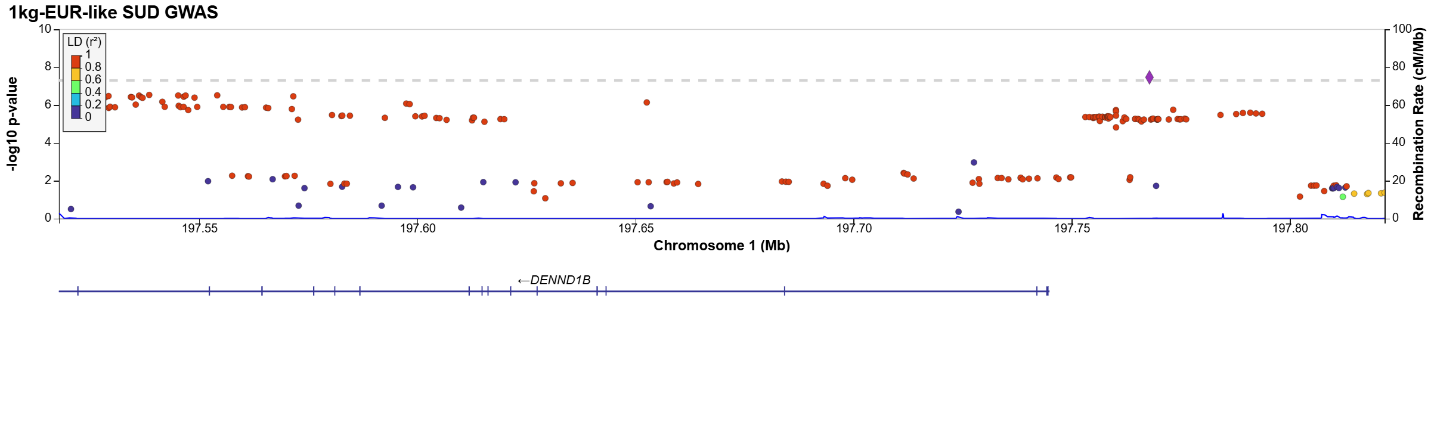


Locus 21:


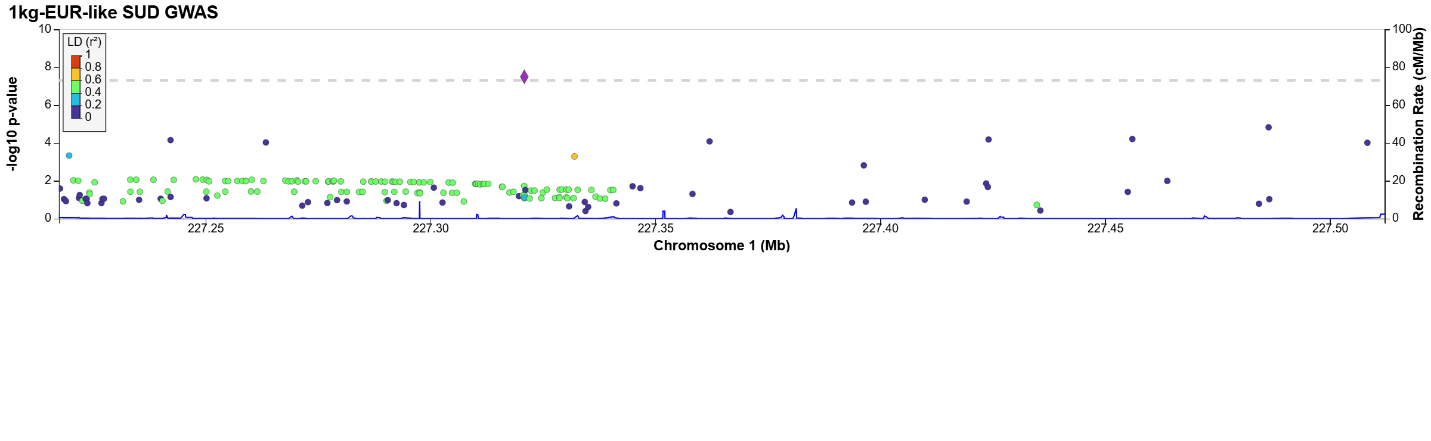


Locus 22:


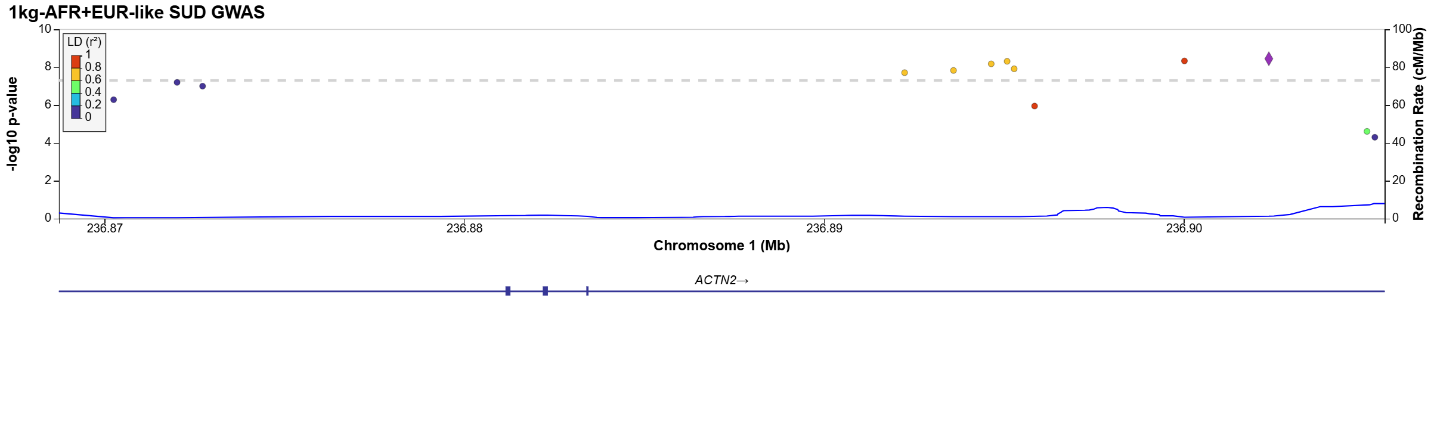


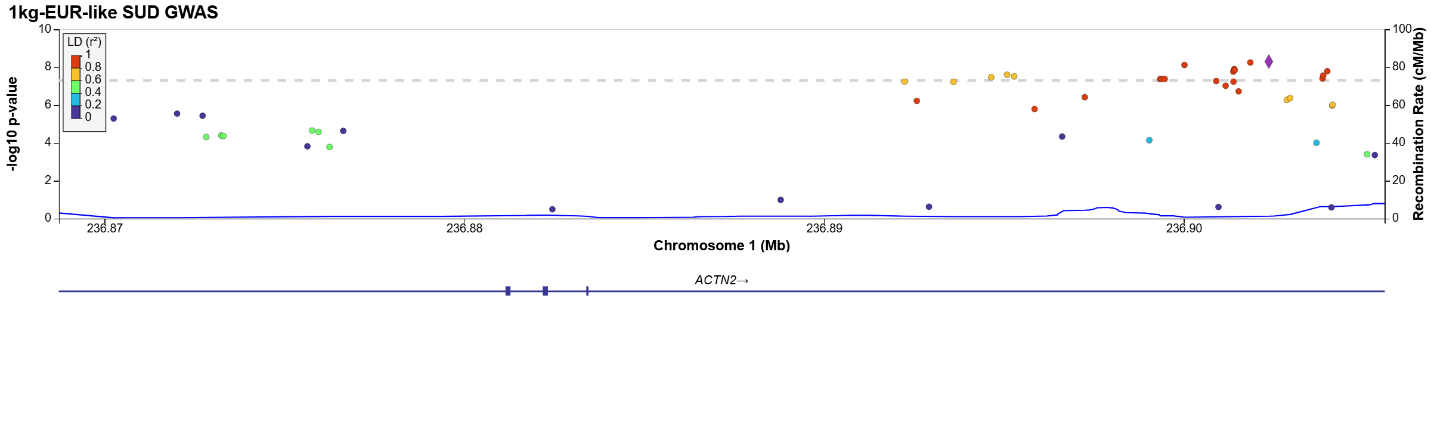


Locus 23:


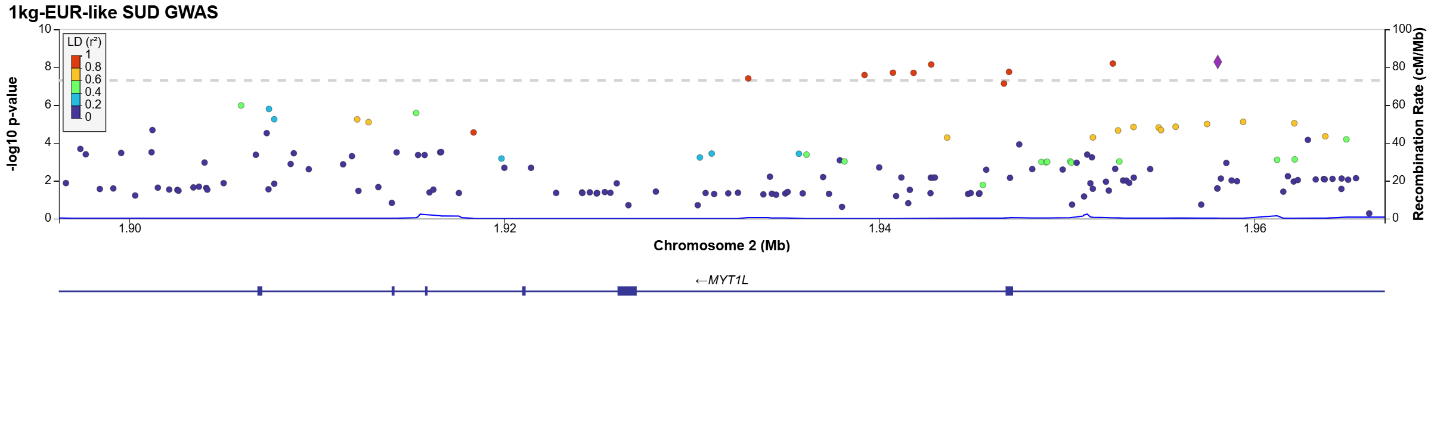


Locus 24:


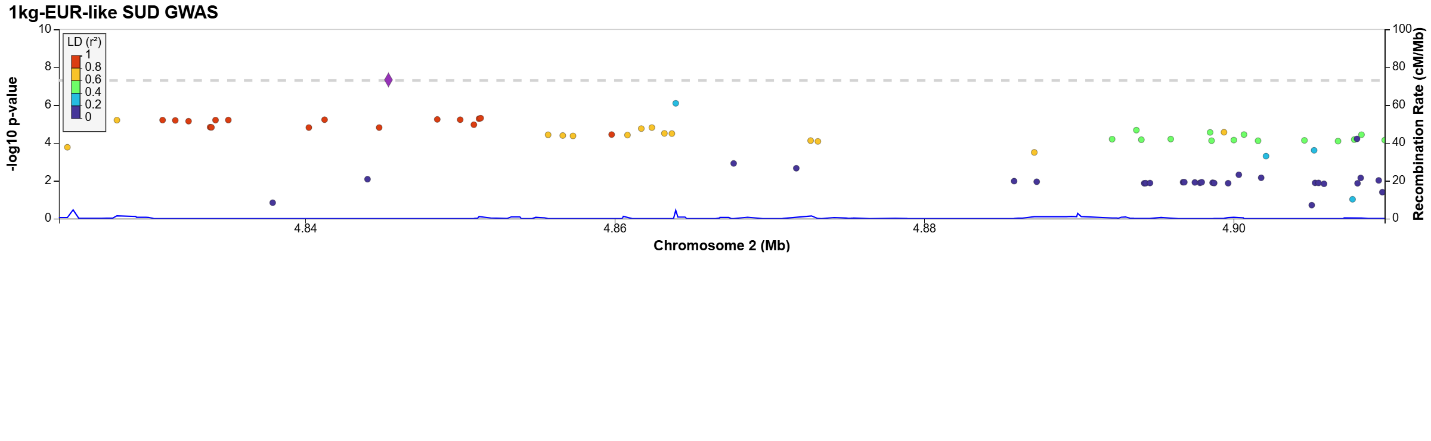


Locus 25:


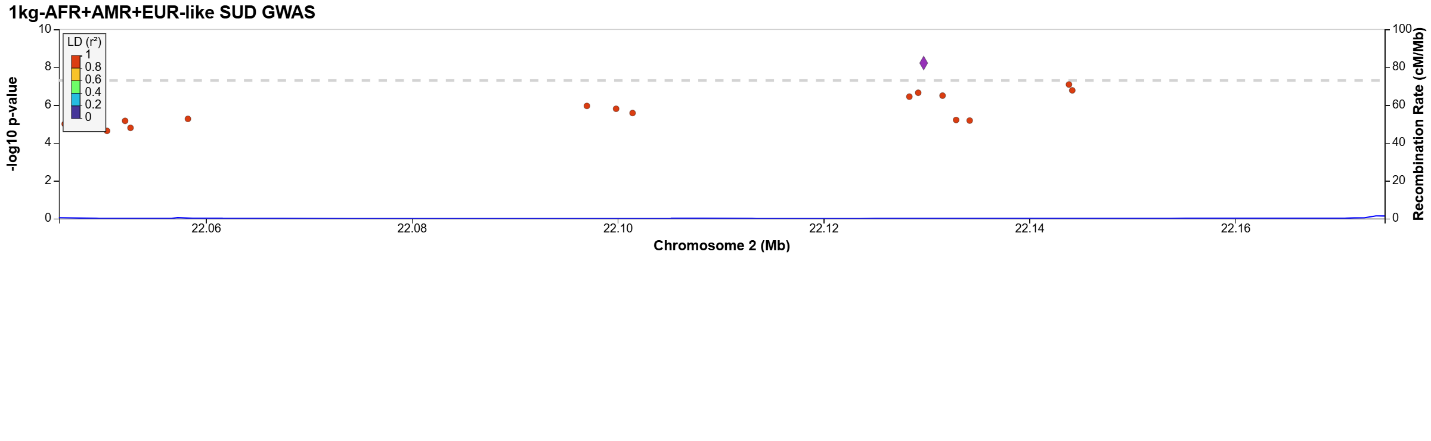

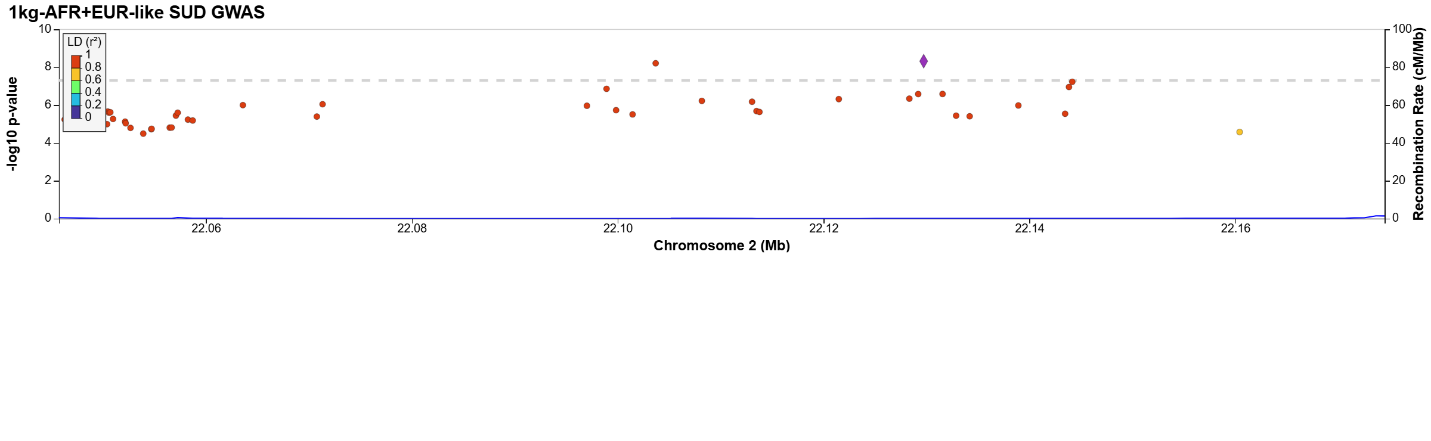


Locus 26:


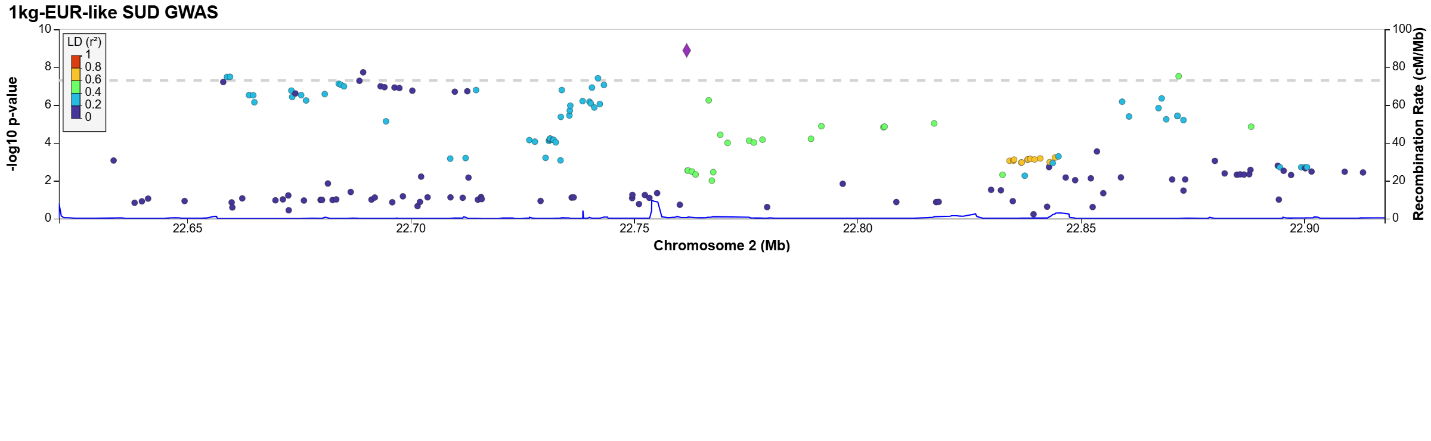


Locus 27:


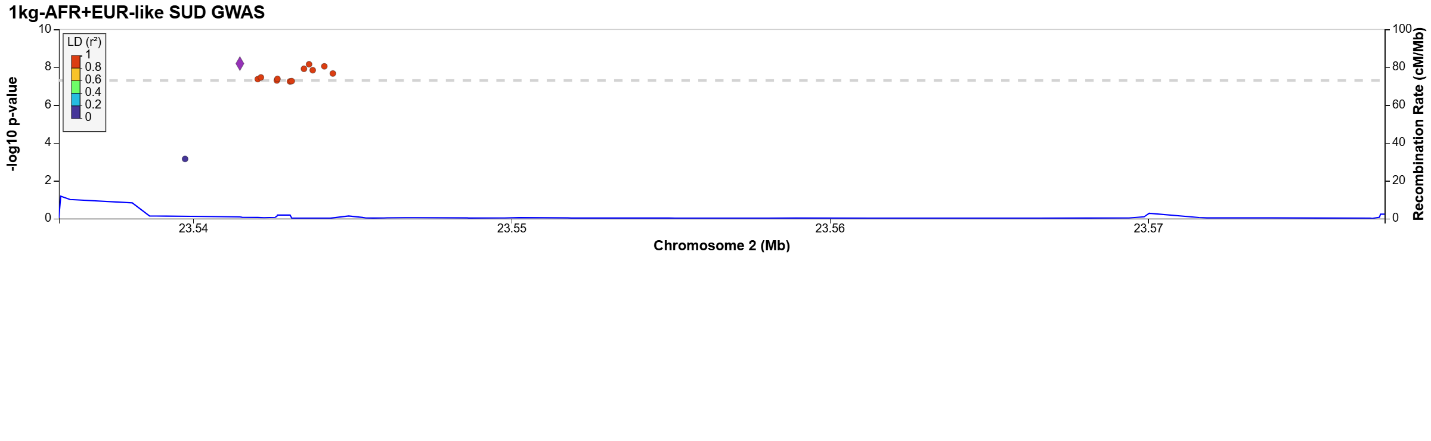


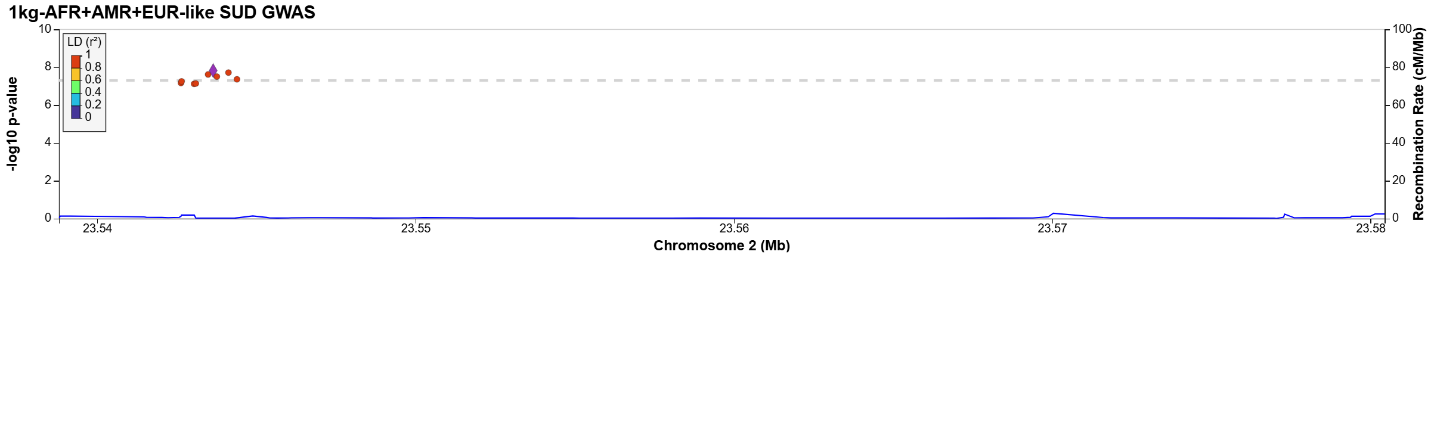


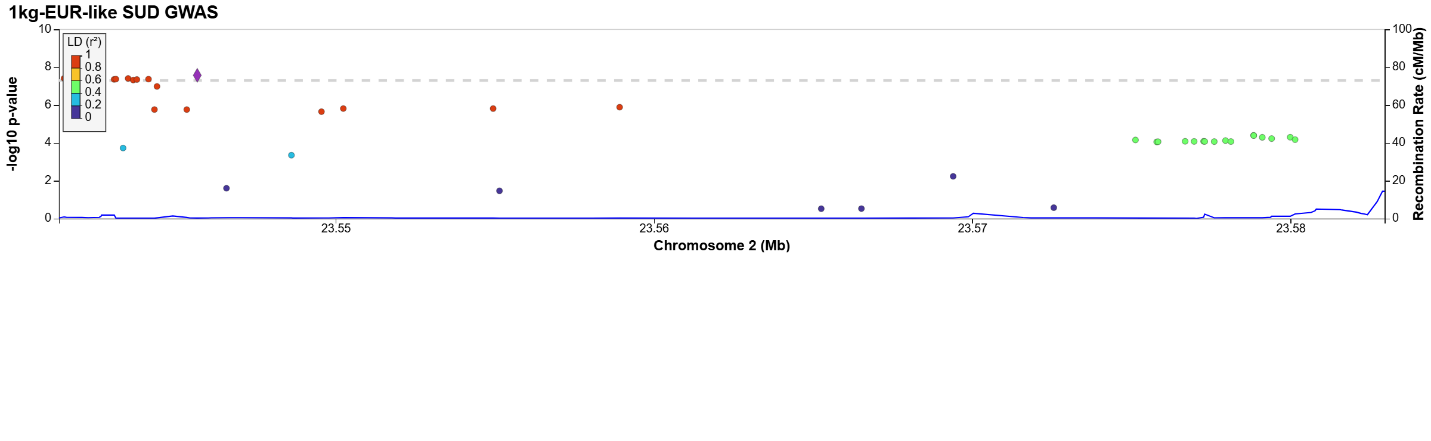


Locus 28:


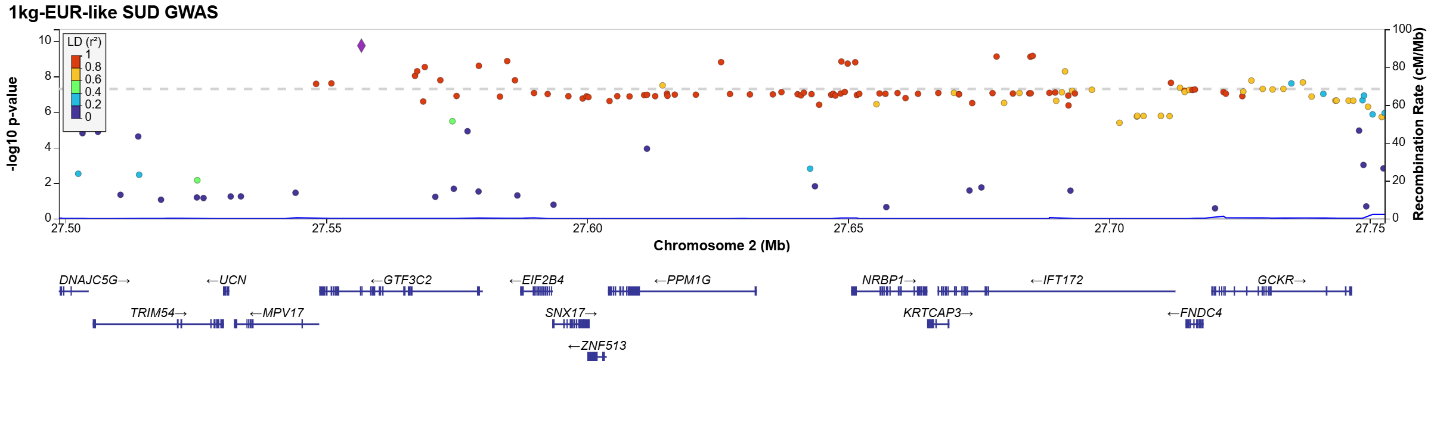


Locus 29:


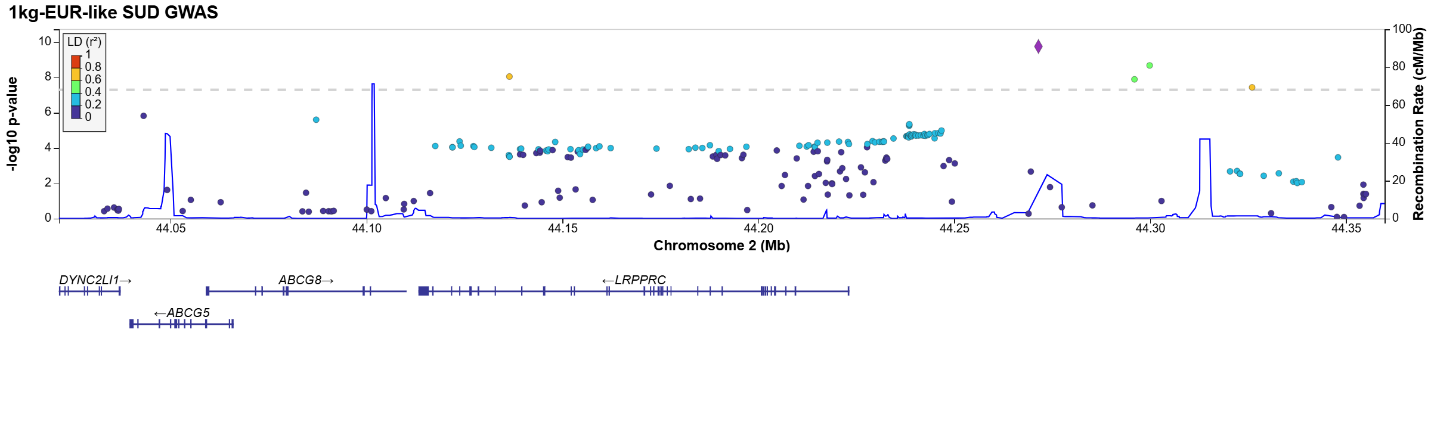


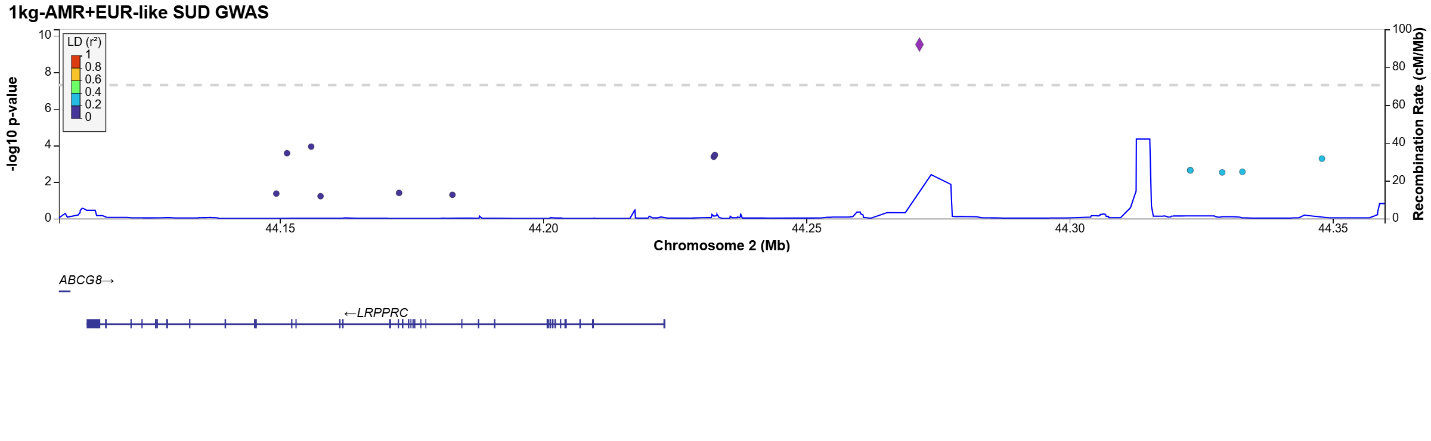


Locus 30:


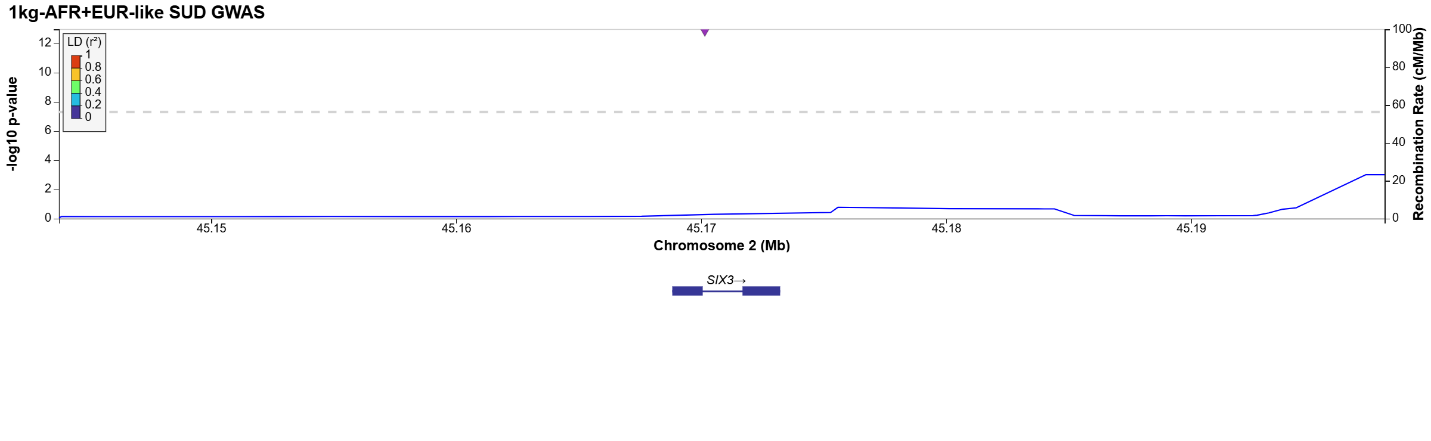


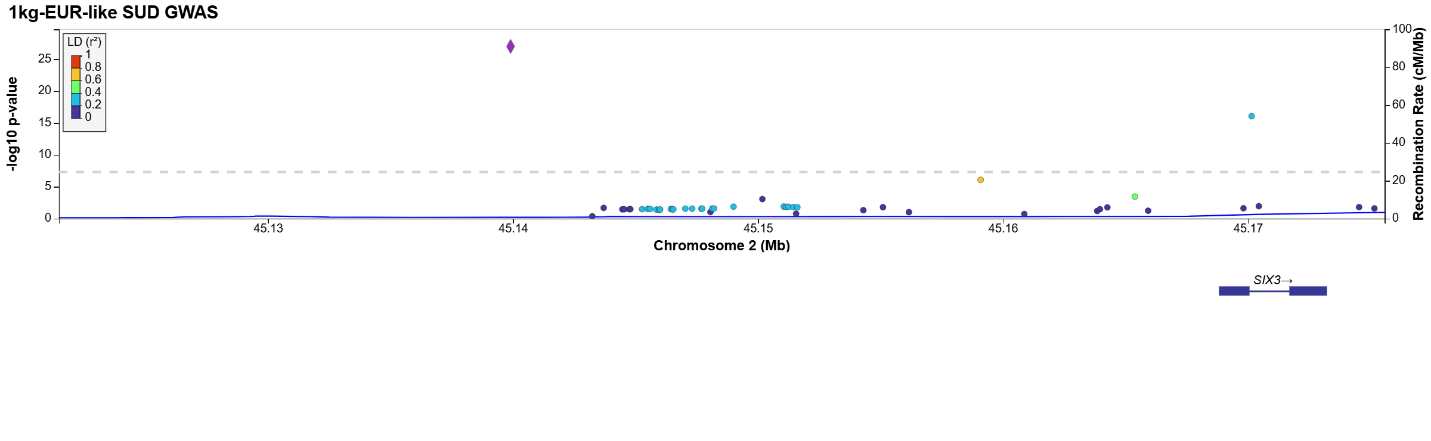


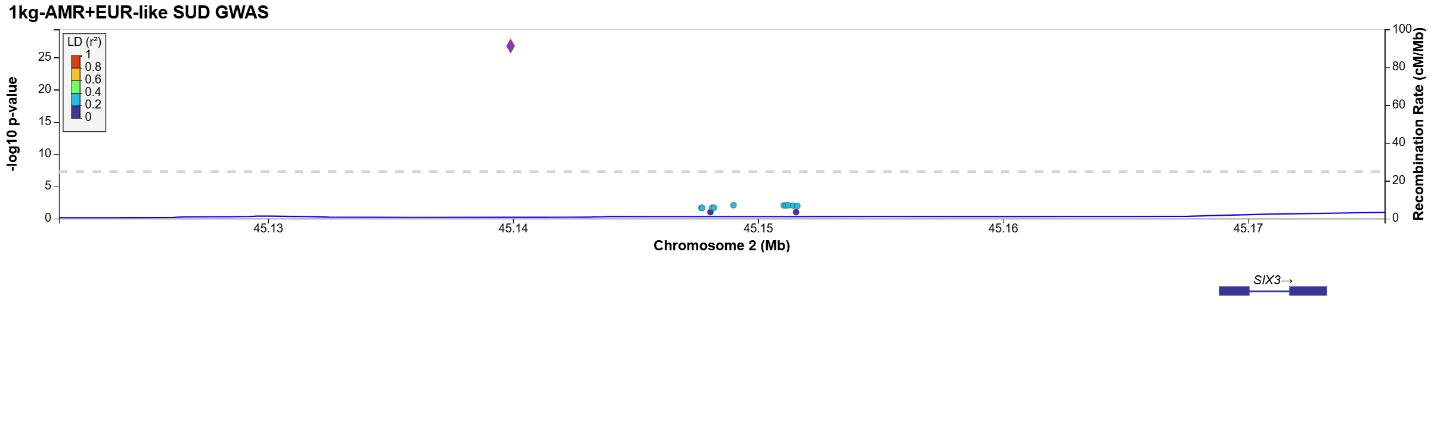


Locus 31:


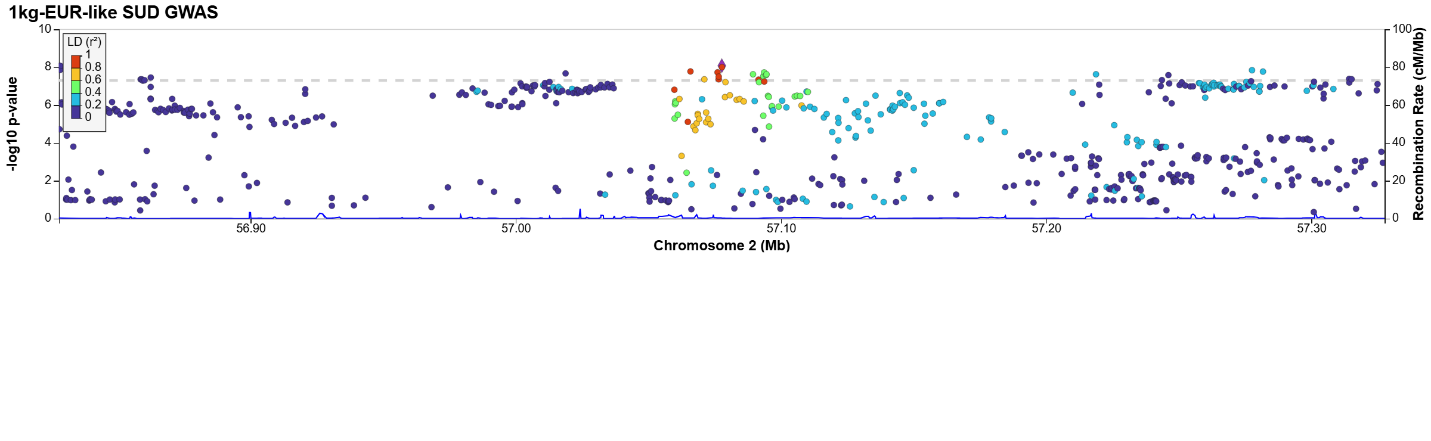


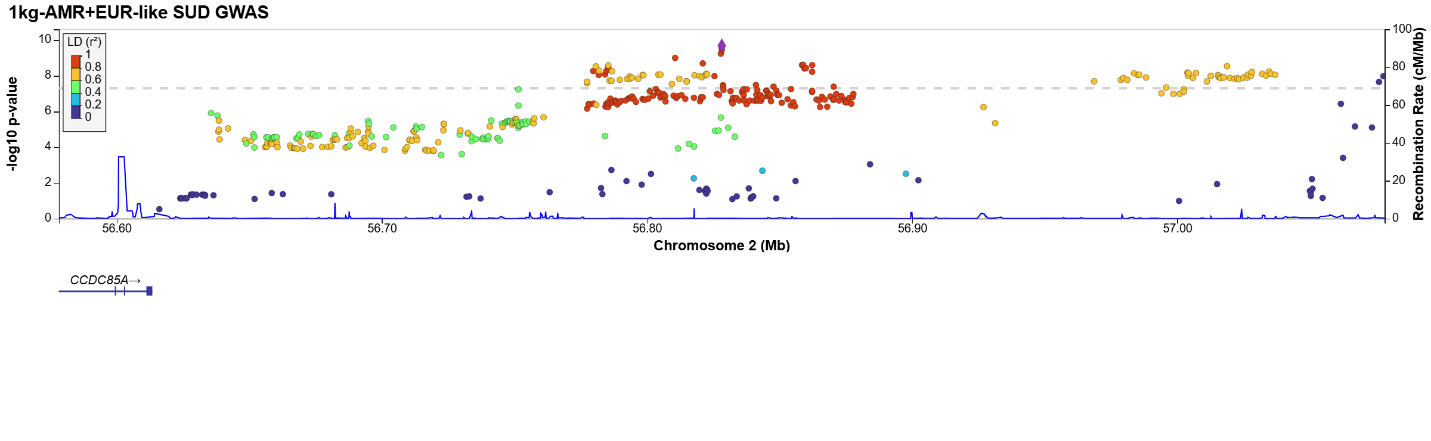


Locus 32:


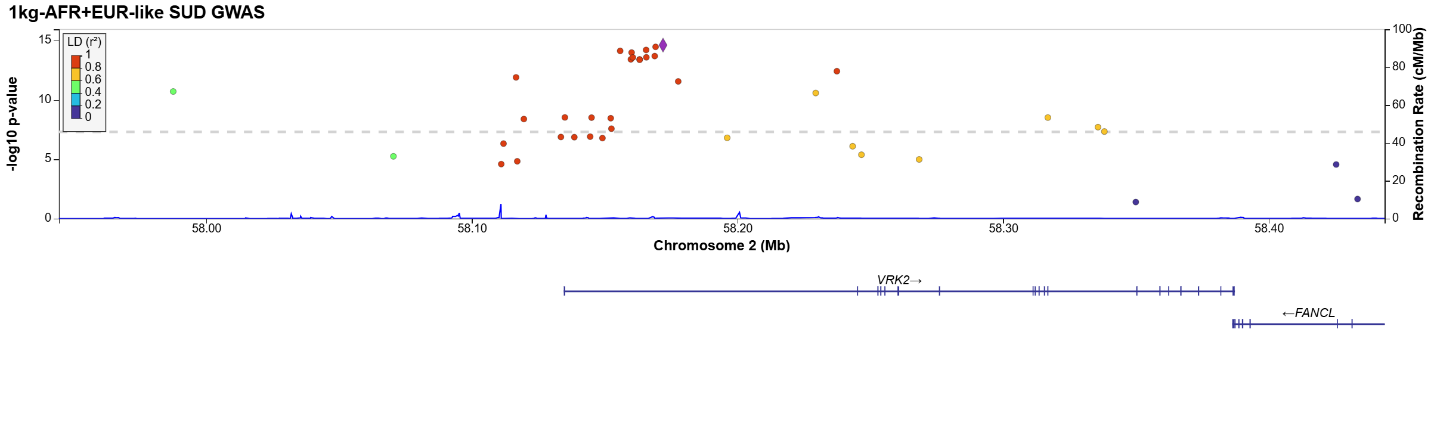


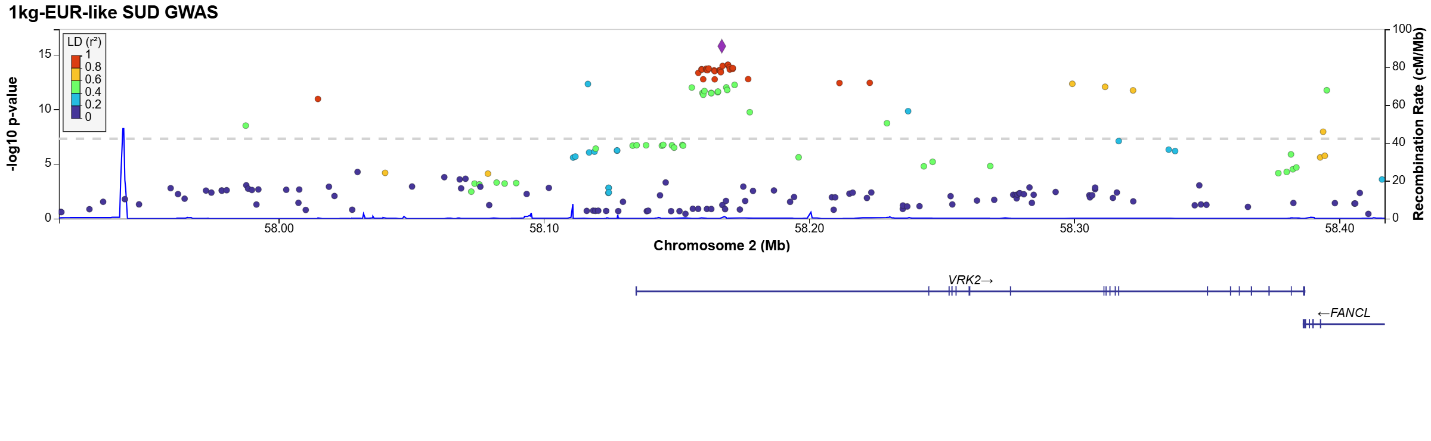


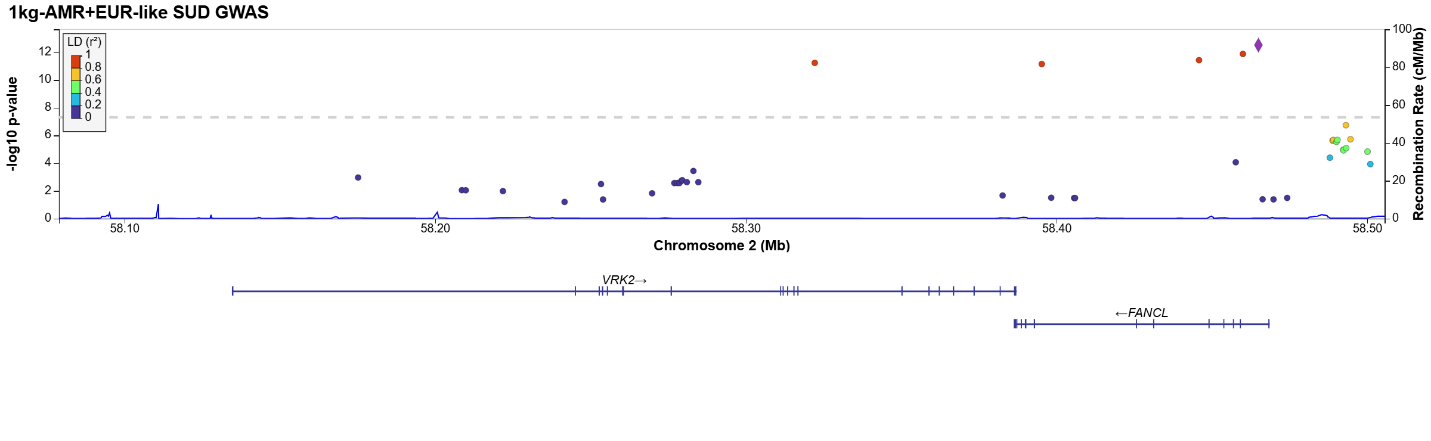


Locus 33:


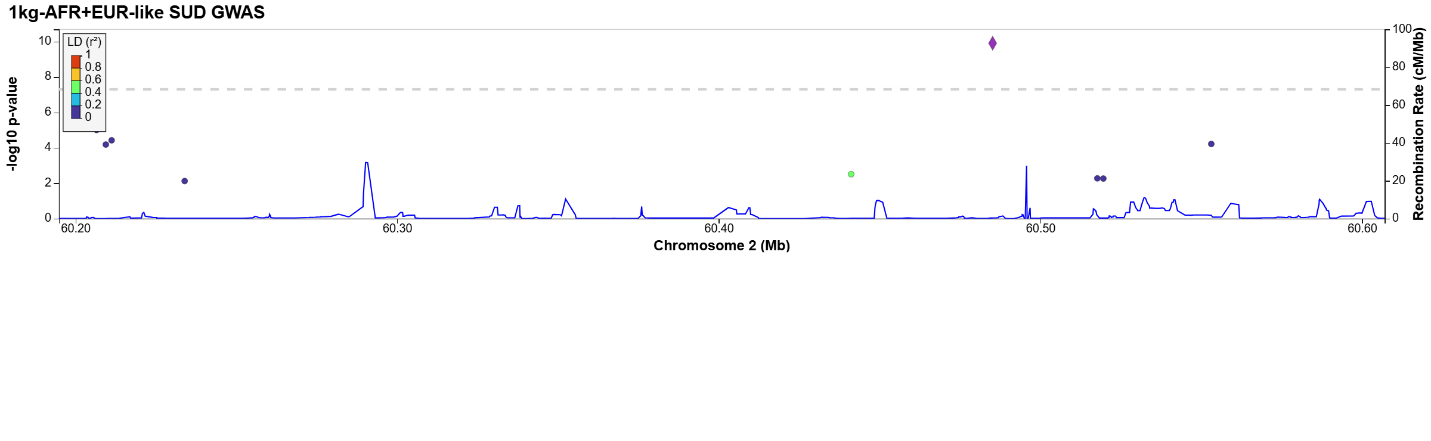


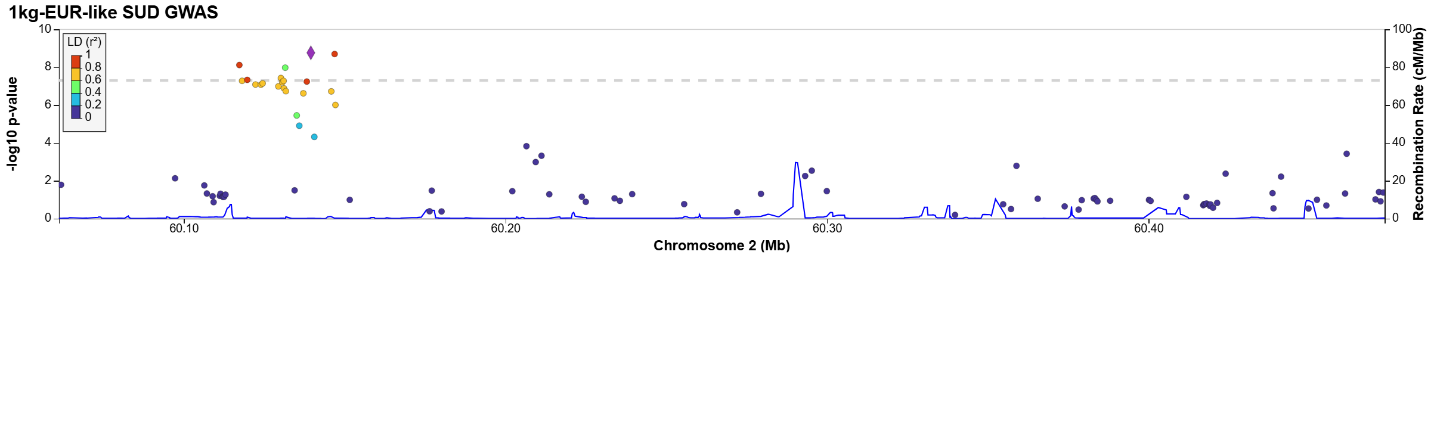


Locus 34:


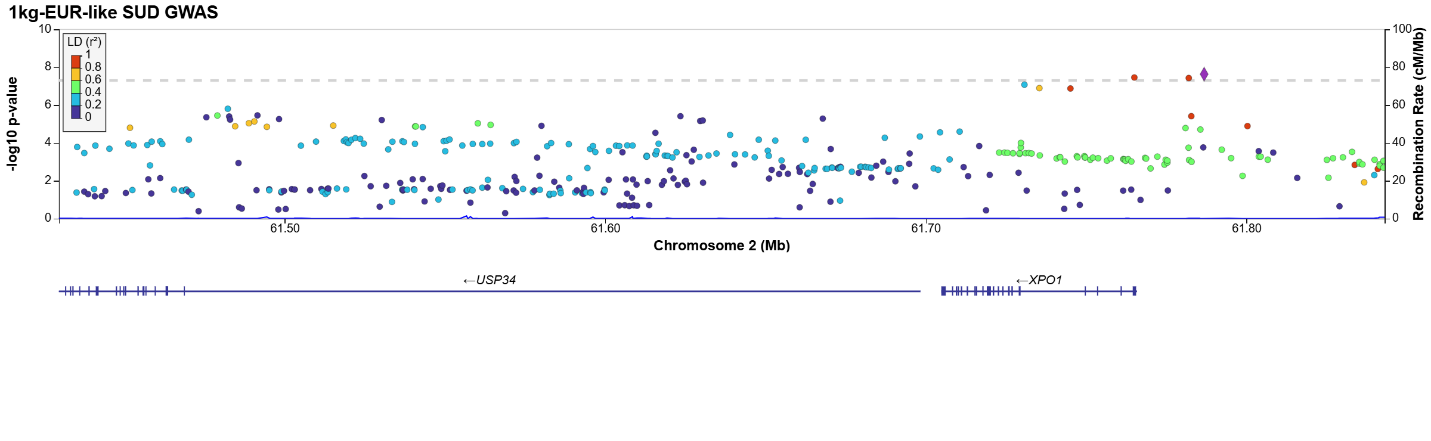


Locus 35:


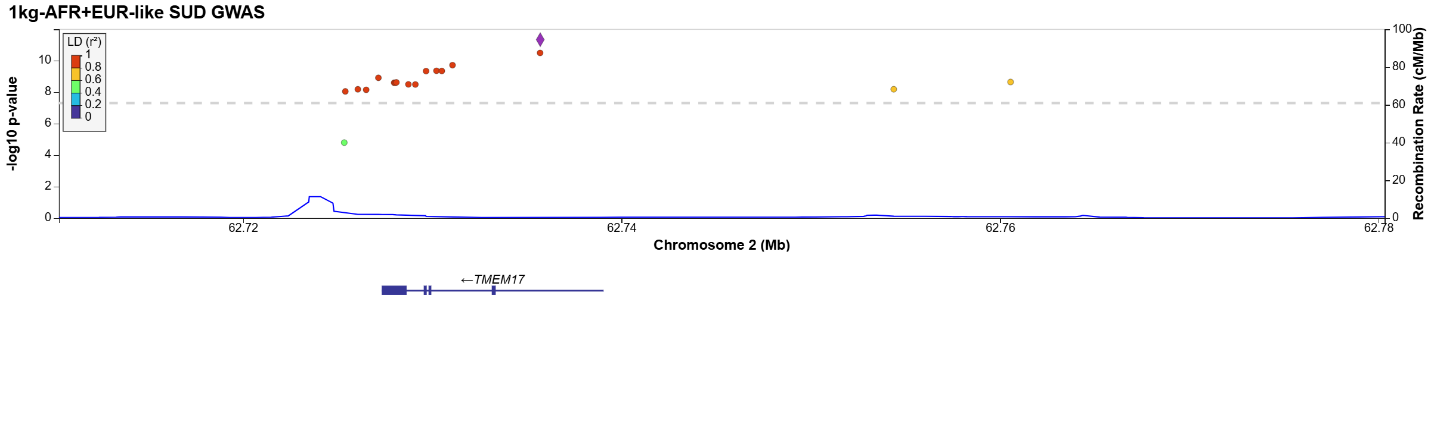


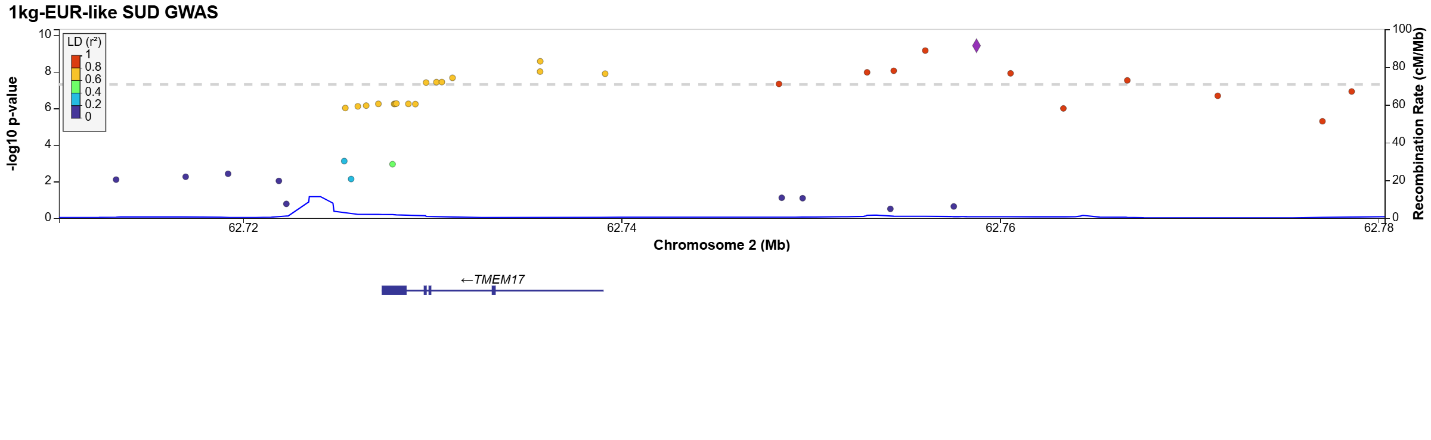


Locus 36:


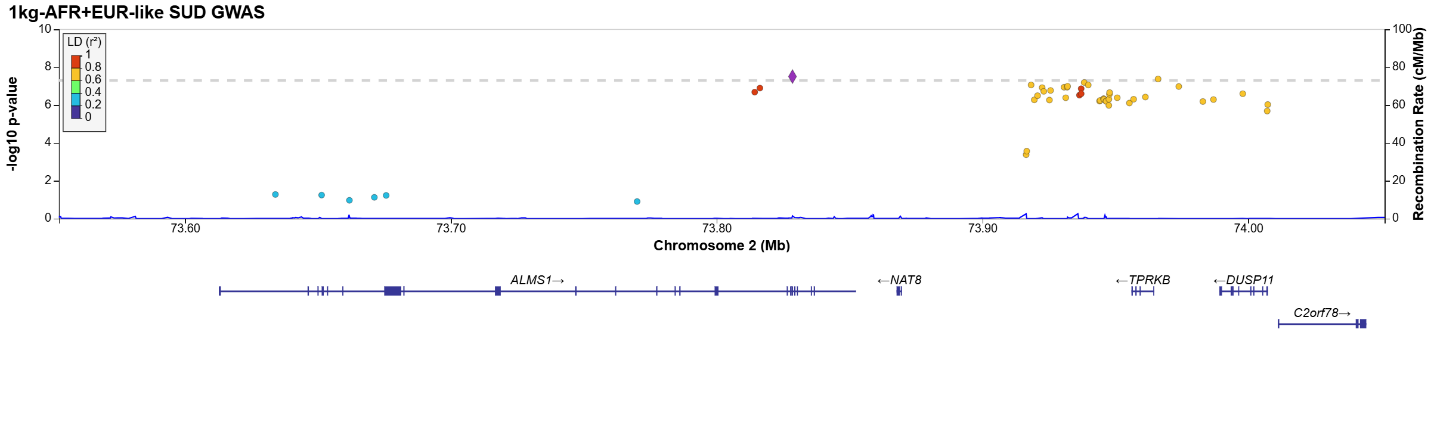


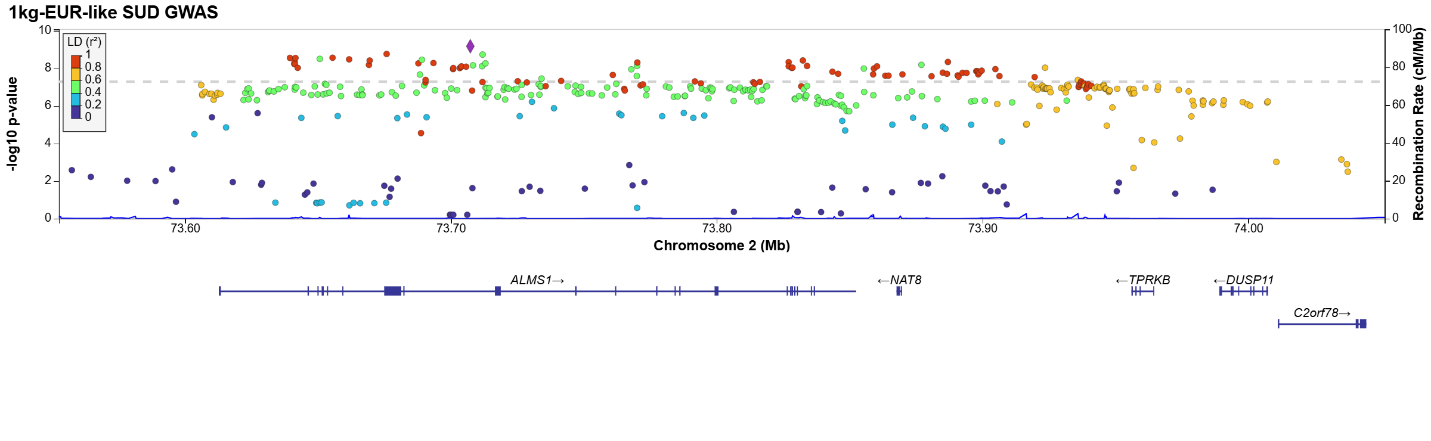


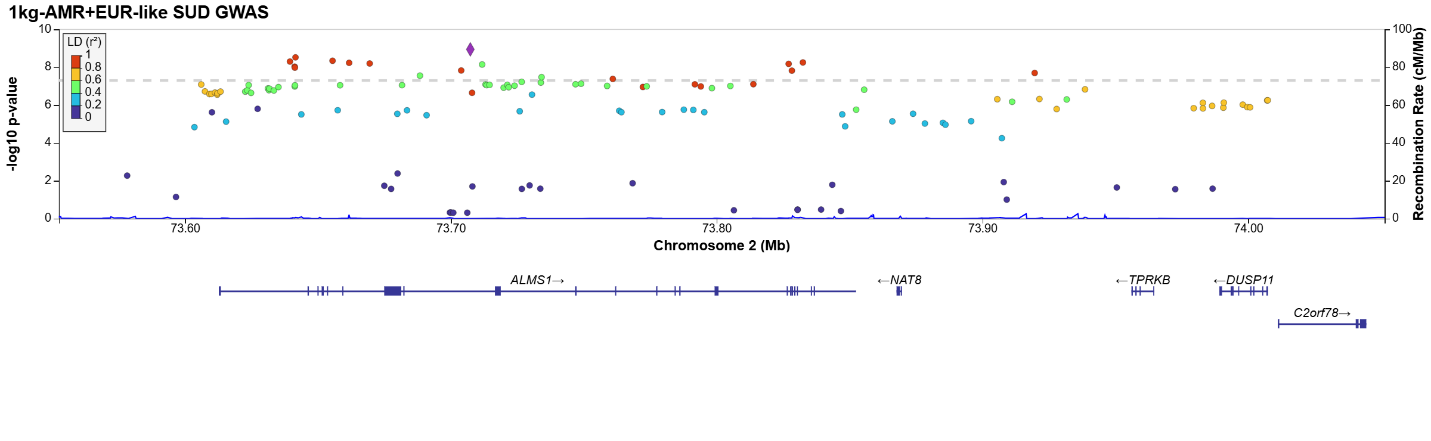


Locus 37:


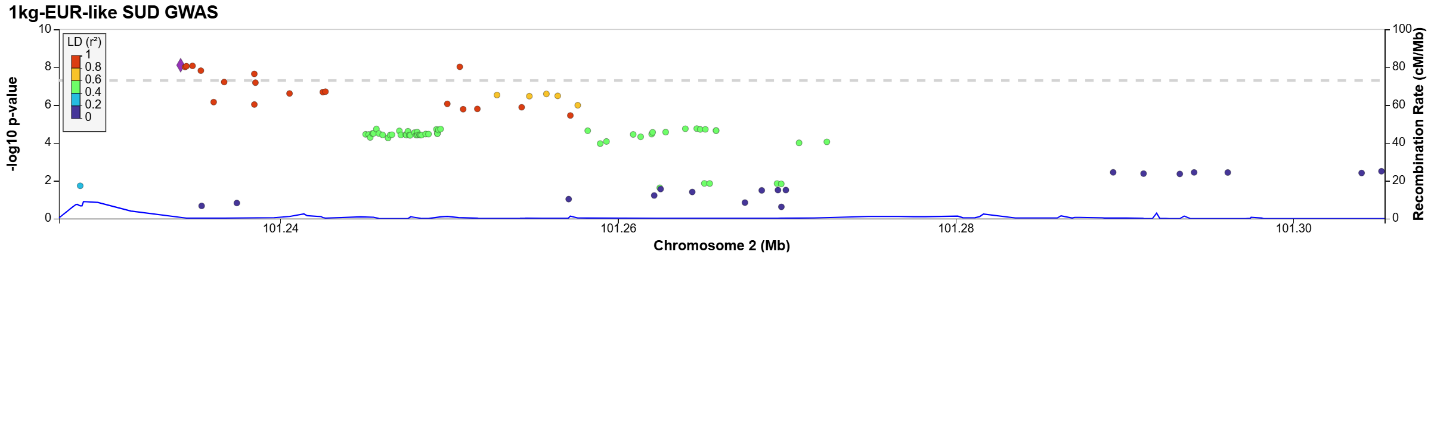


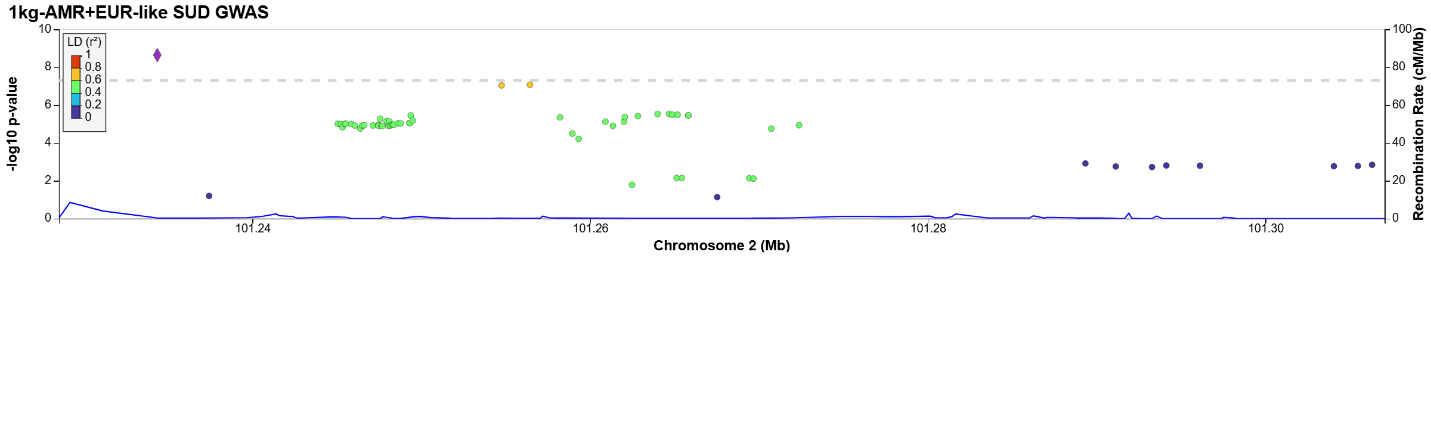


Locus 38:


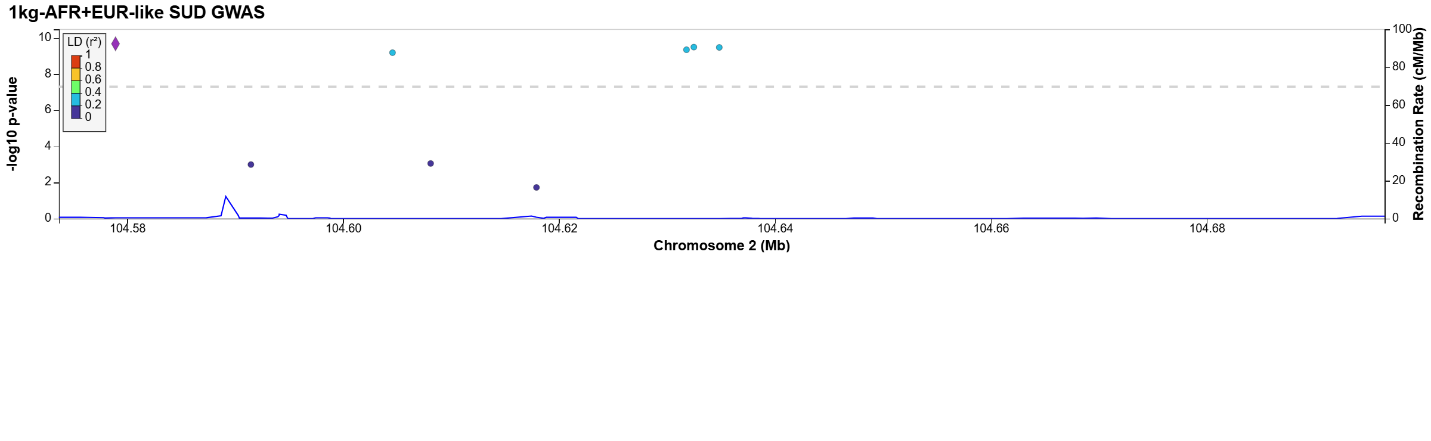


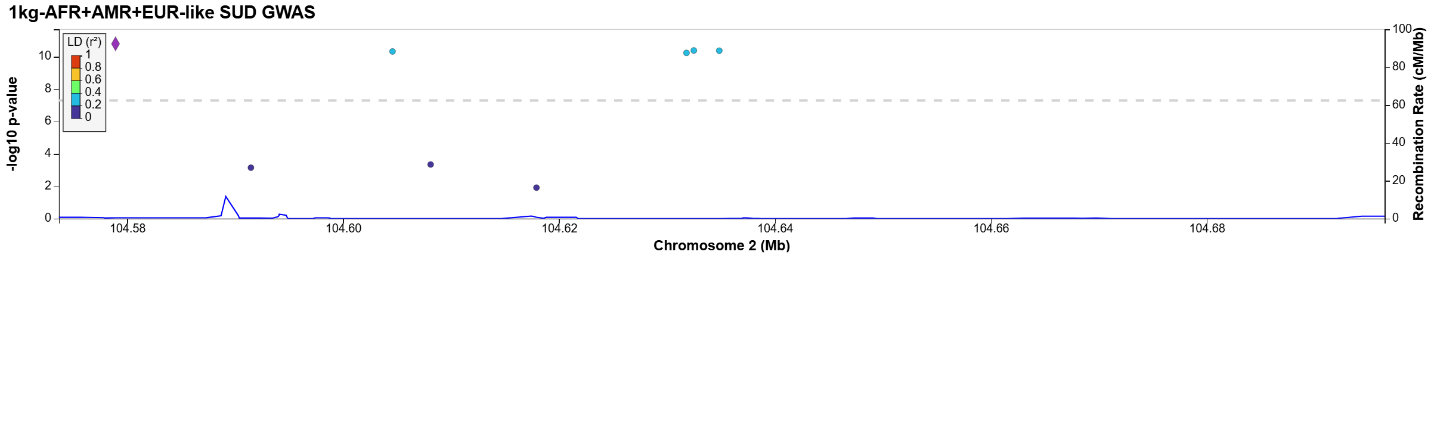


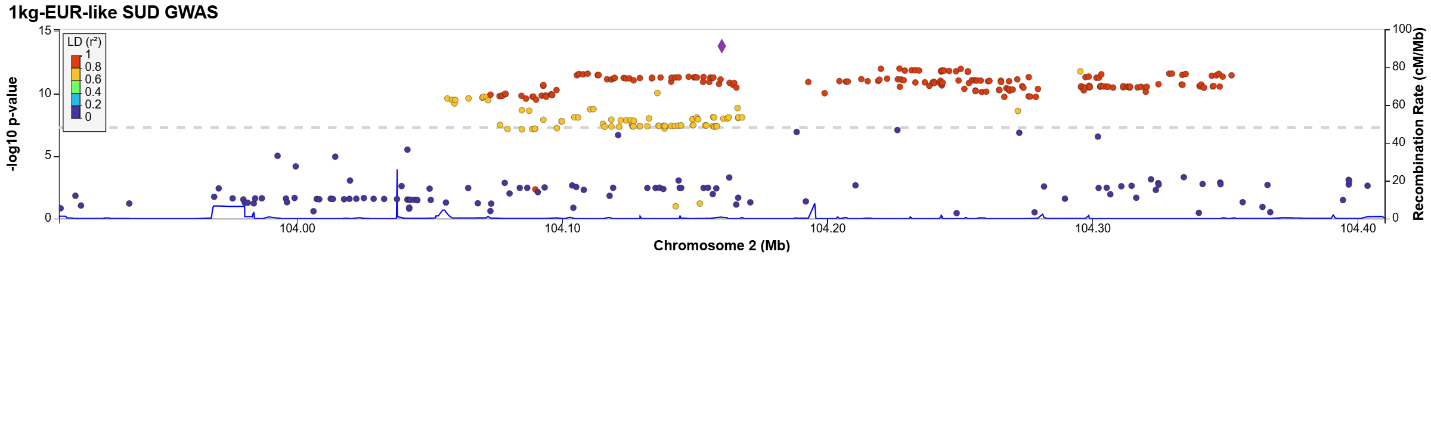


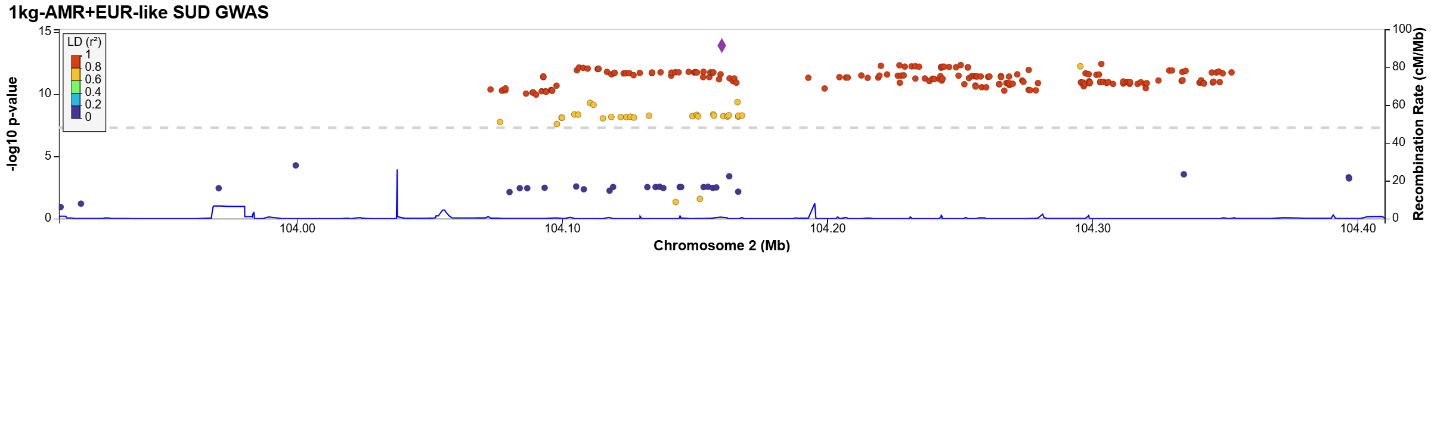


Locus 39:


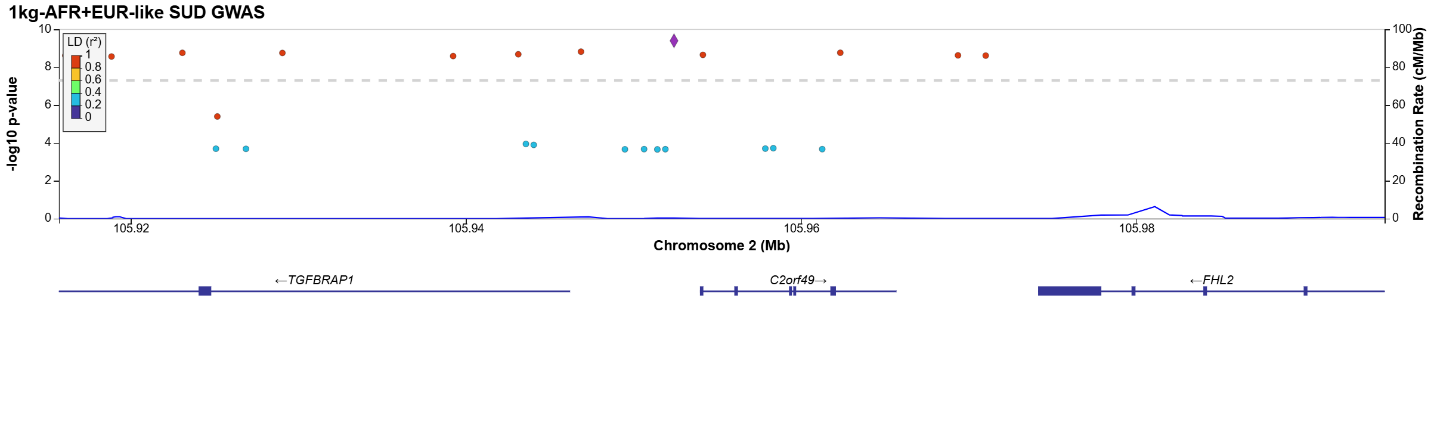


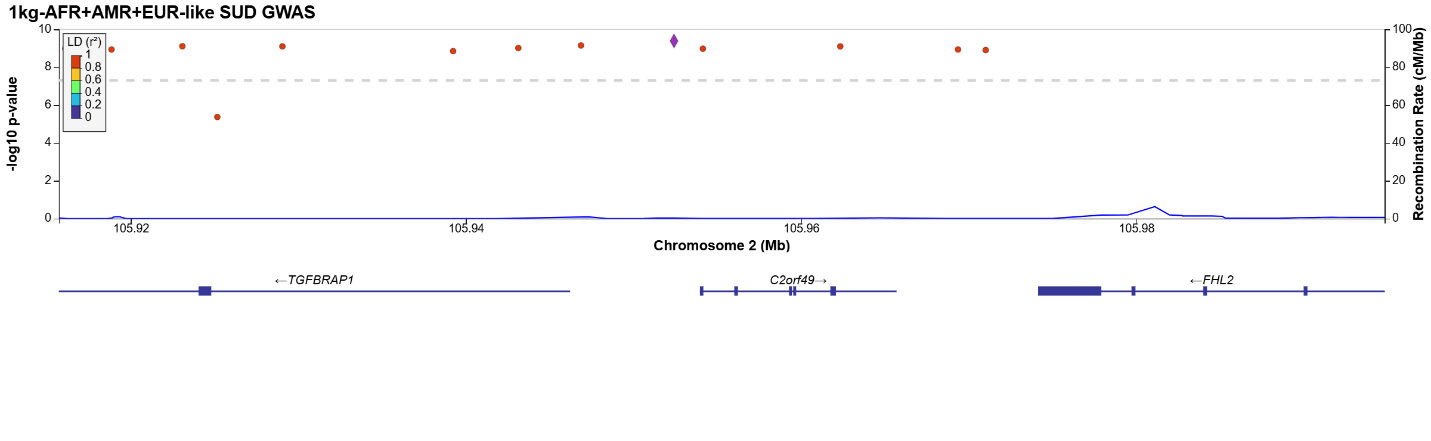


Locus 40:


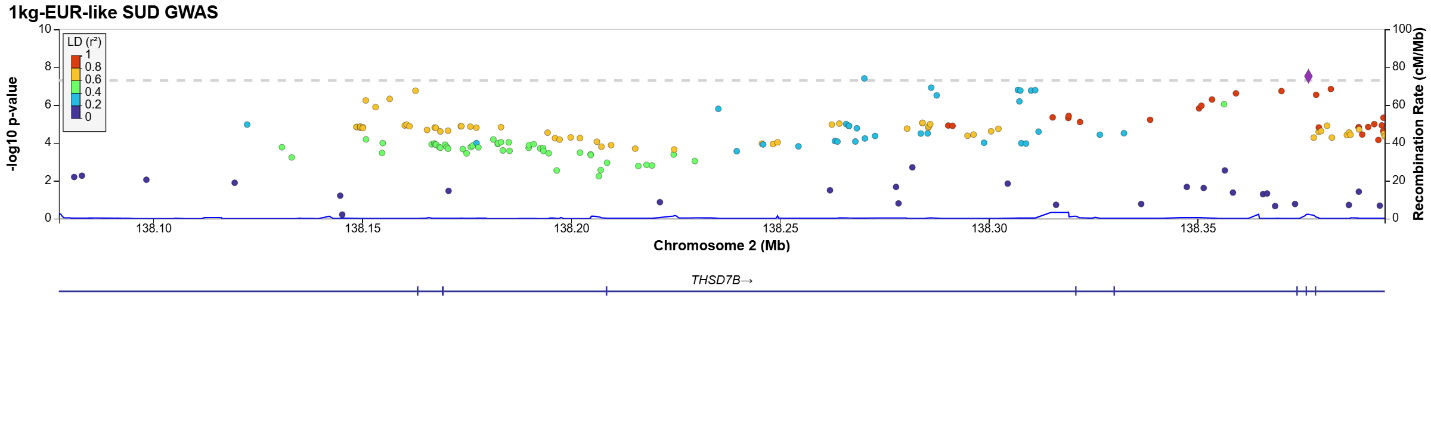


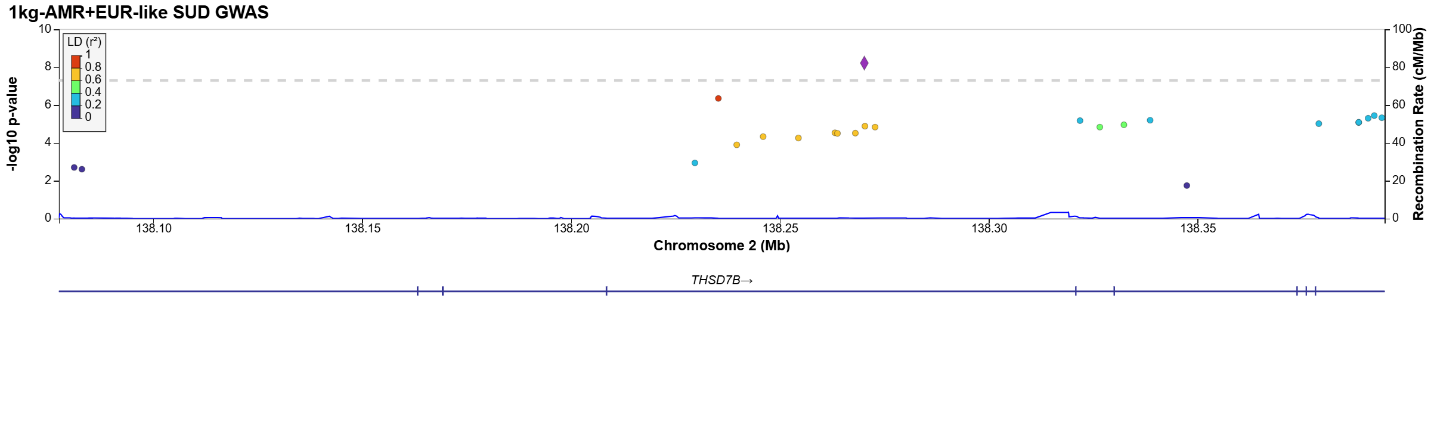


Locus 41:


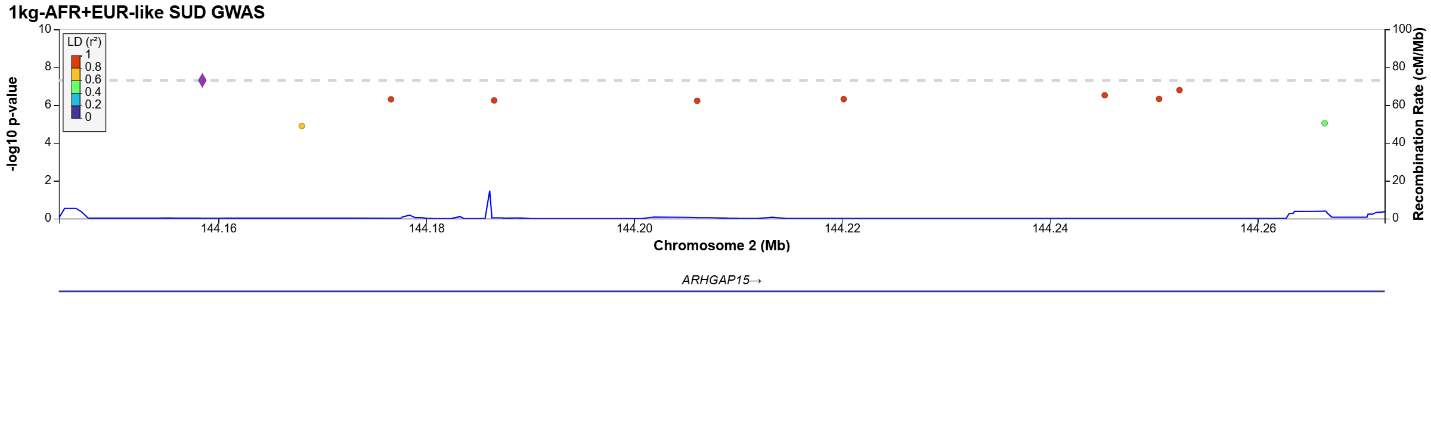


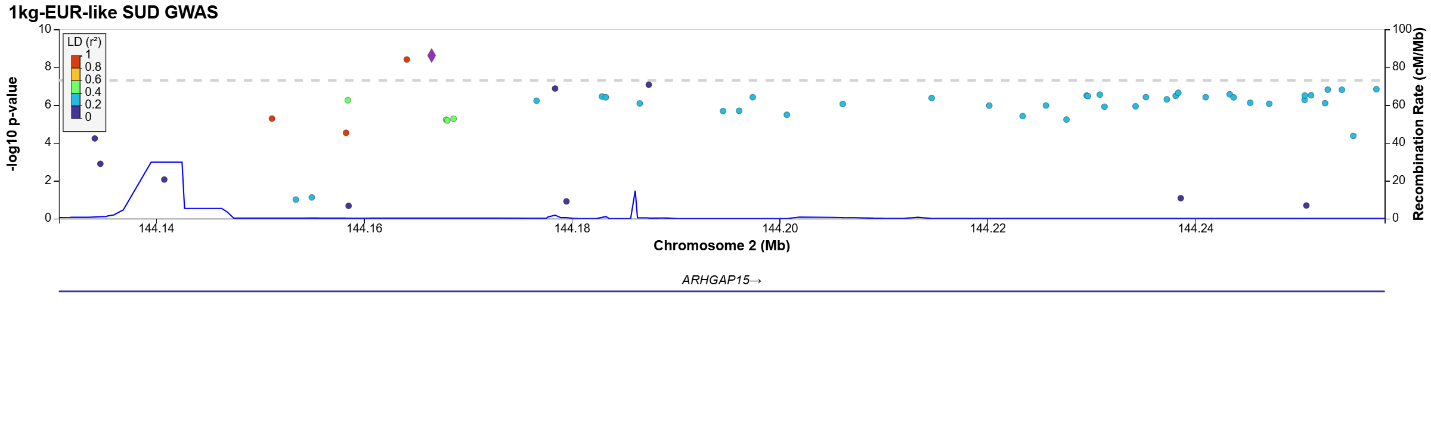


Locus 42:


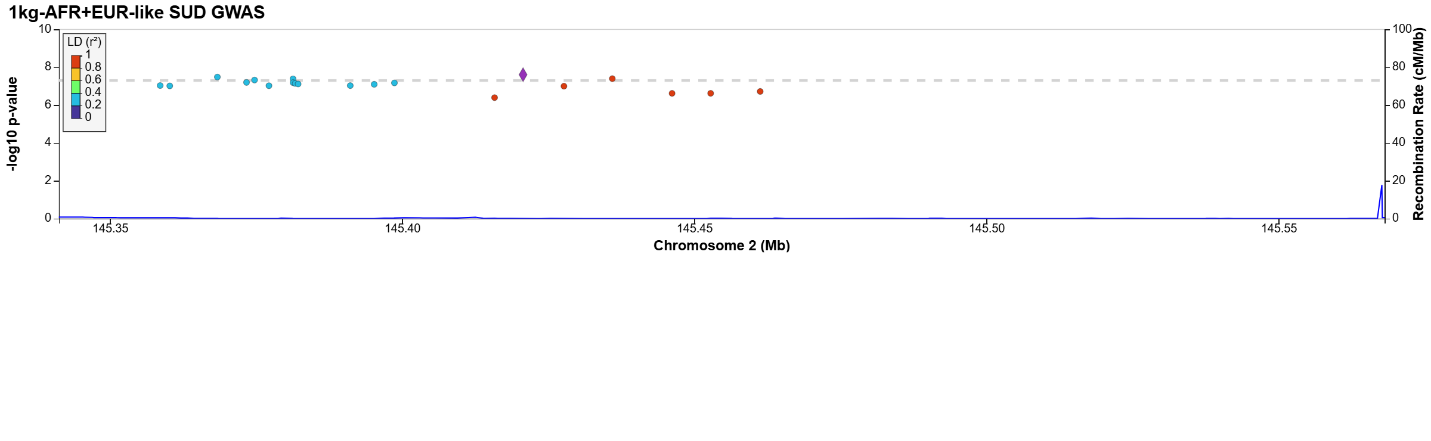


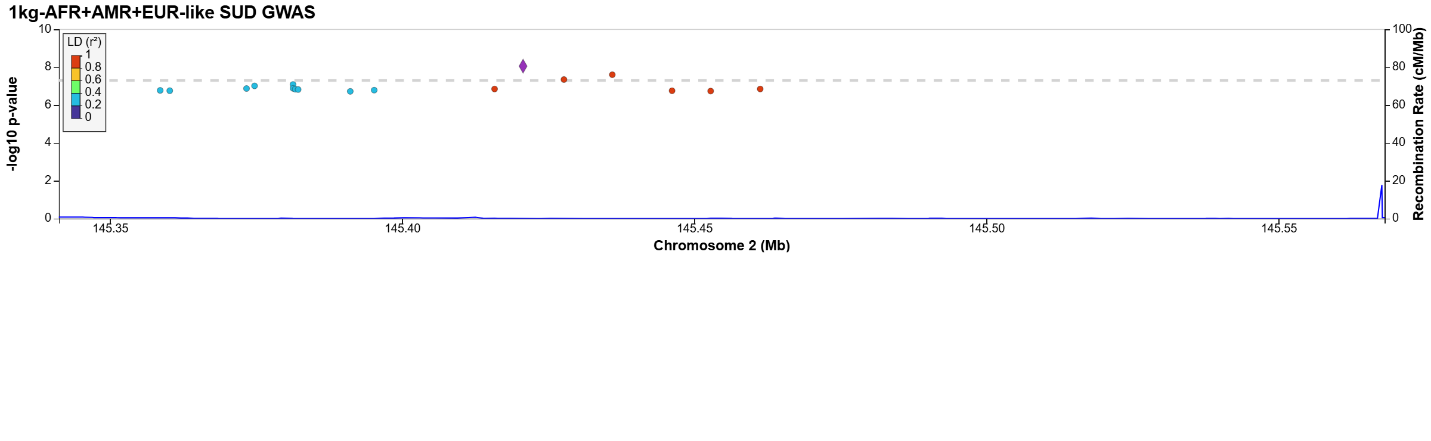


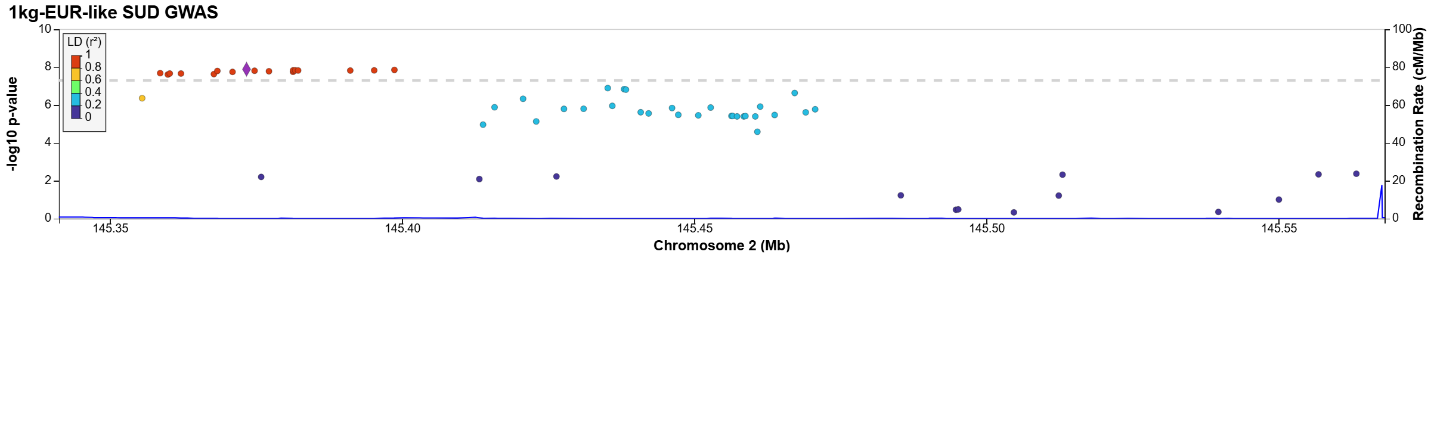


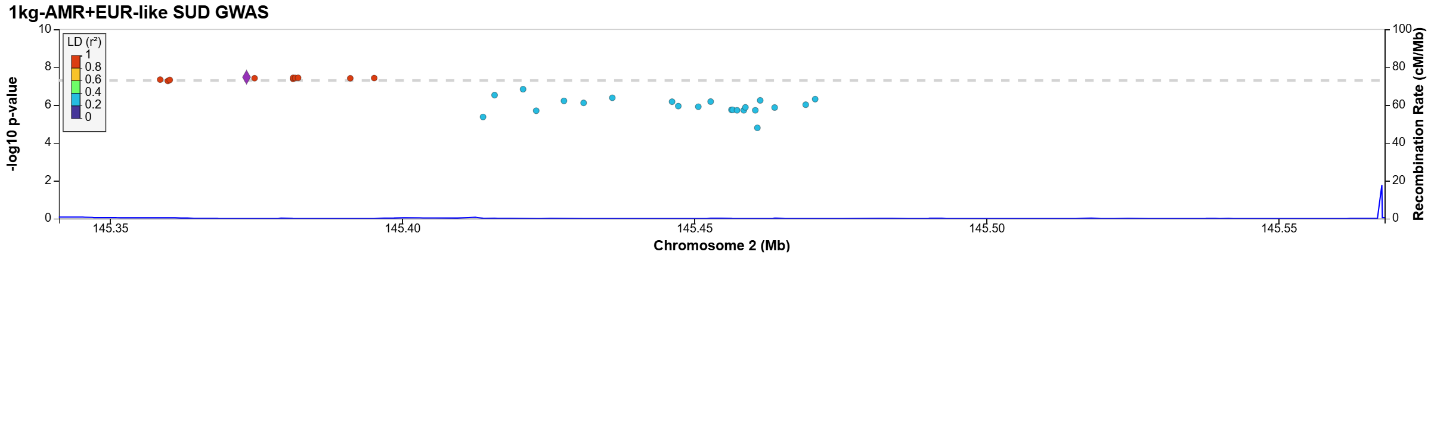


Locus 43:


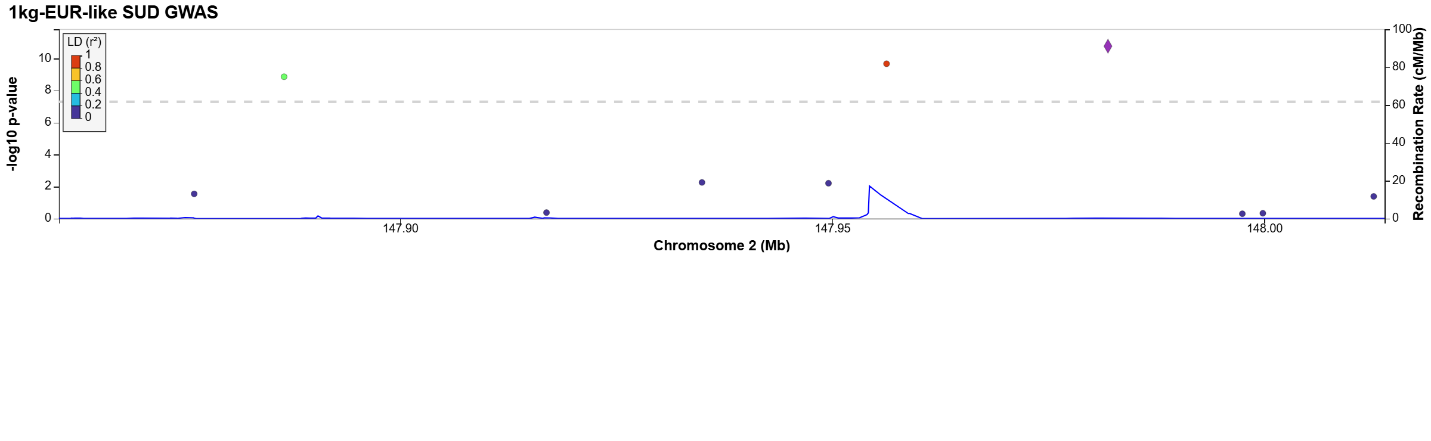


Locus 44:


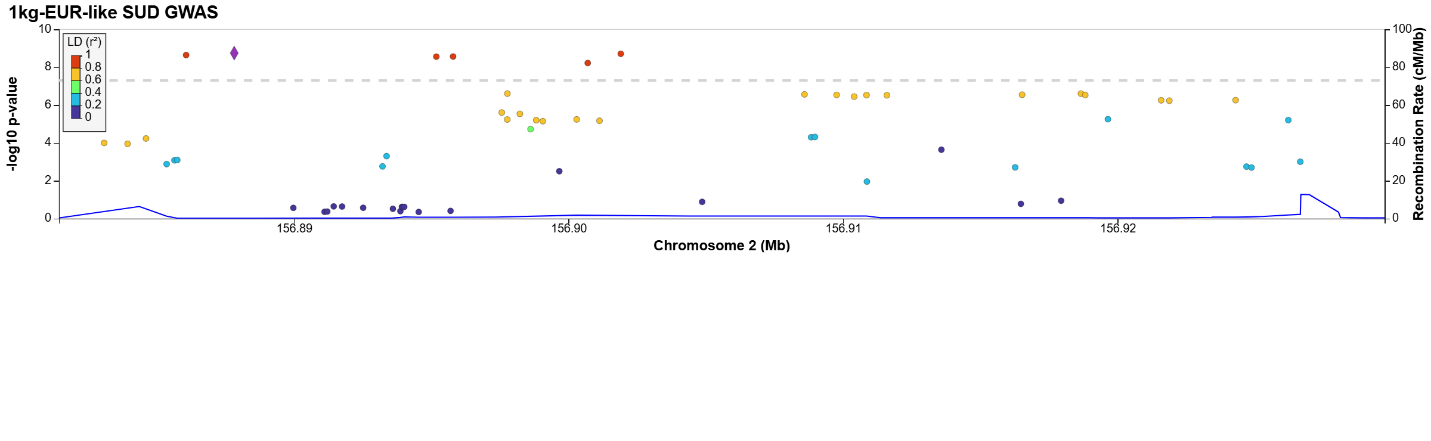


Locus 45:


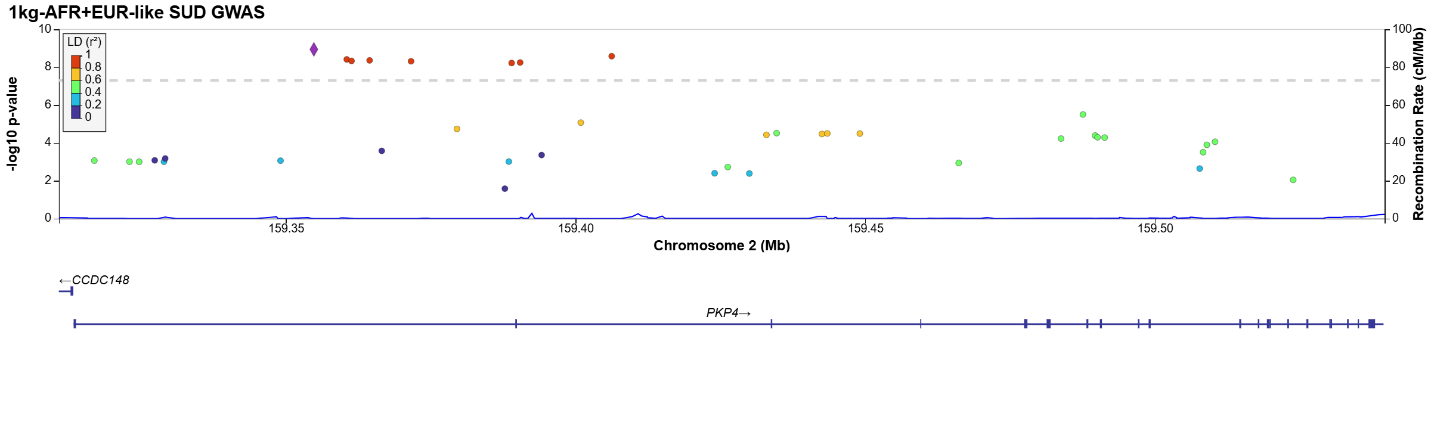


Locus 46:


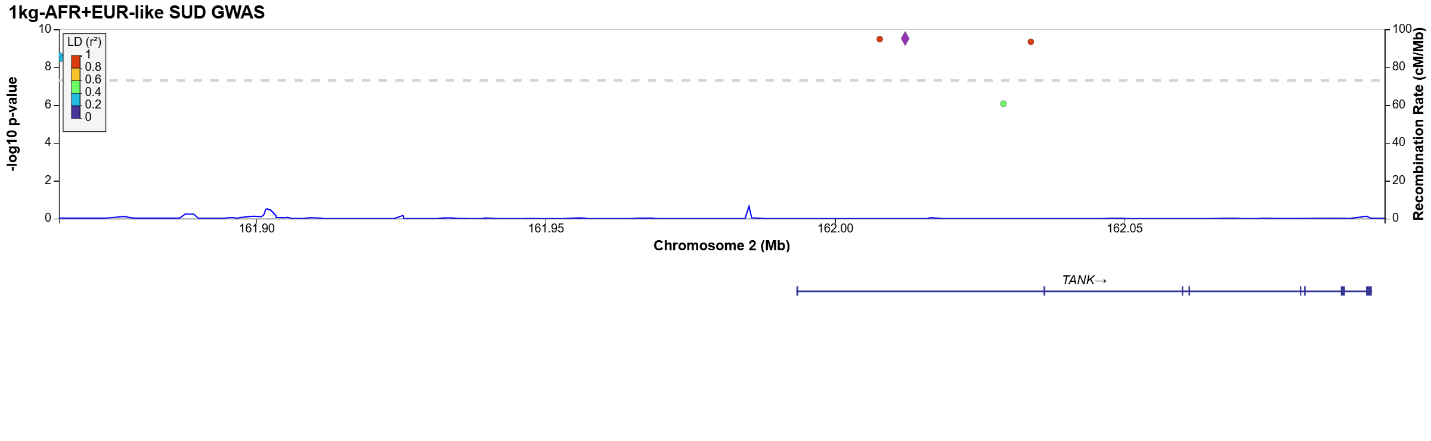


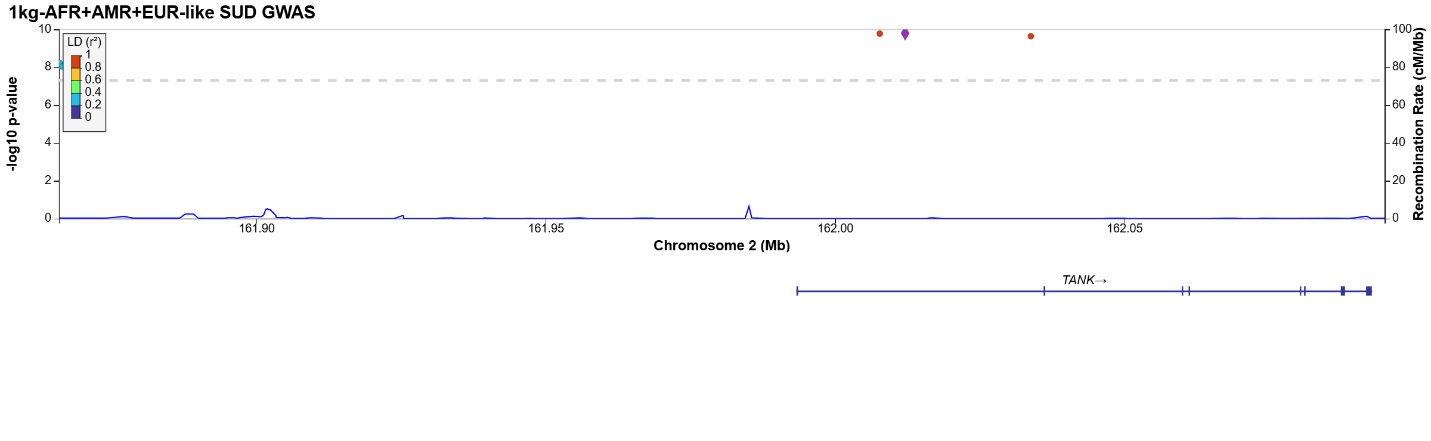


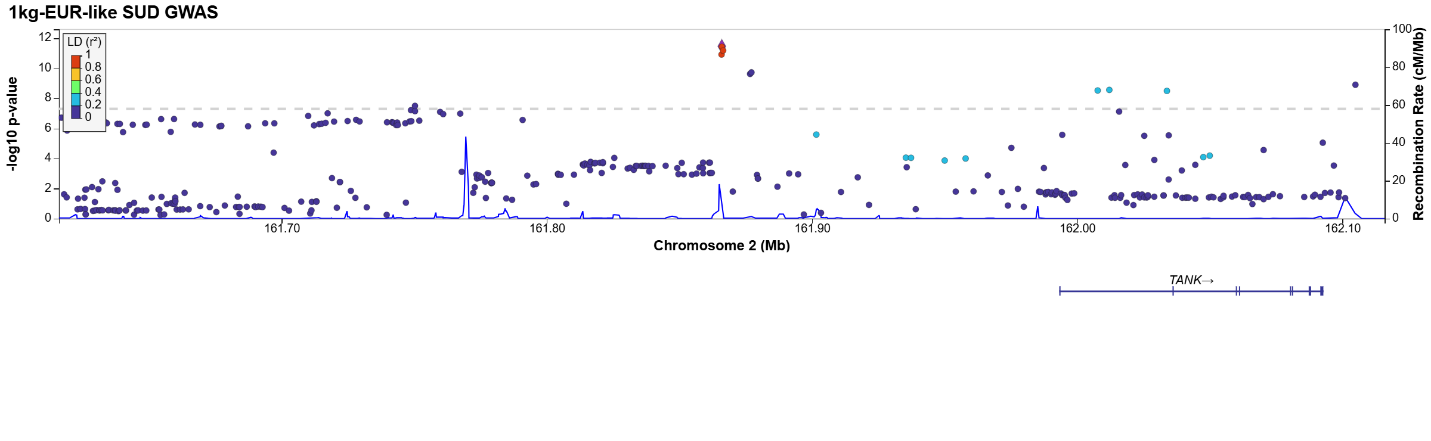


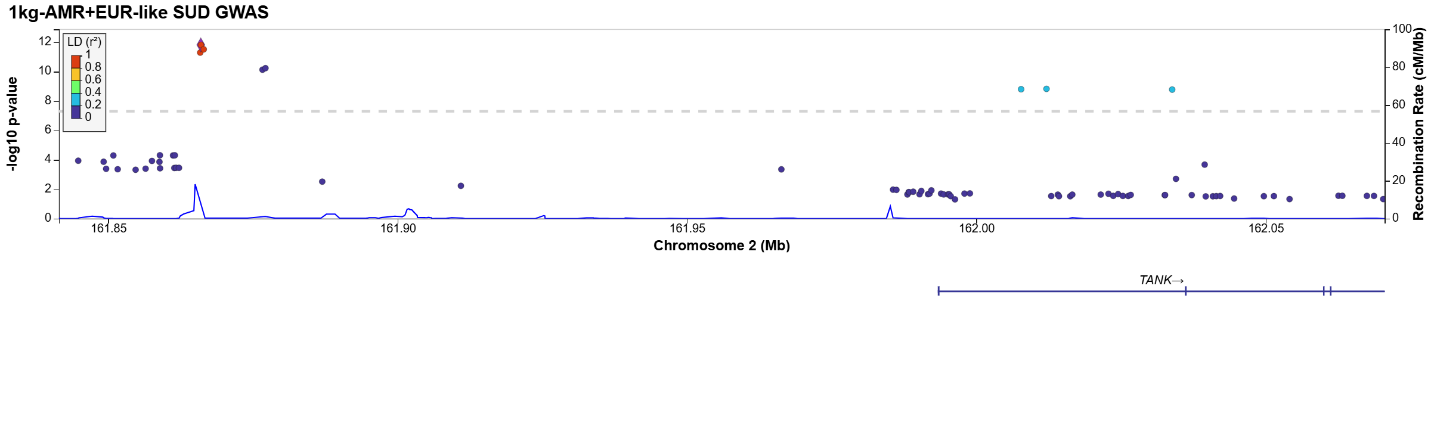


Locus 47:


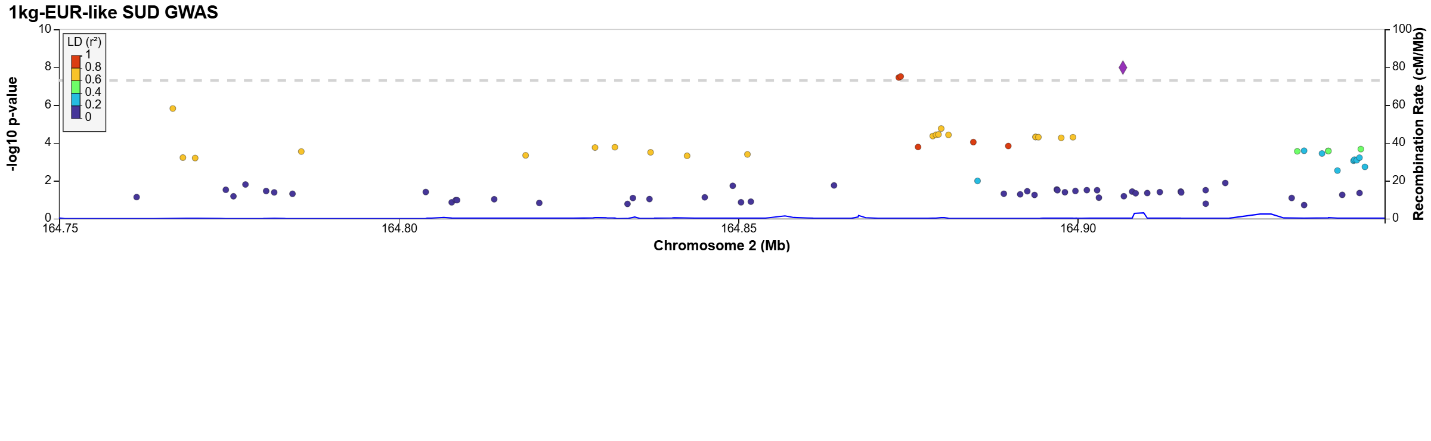


Locus 48:


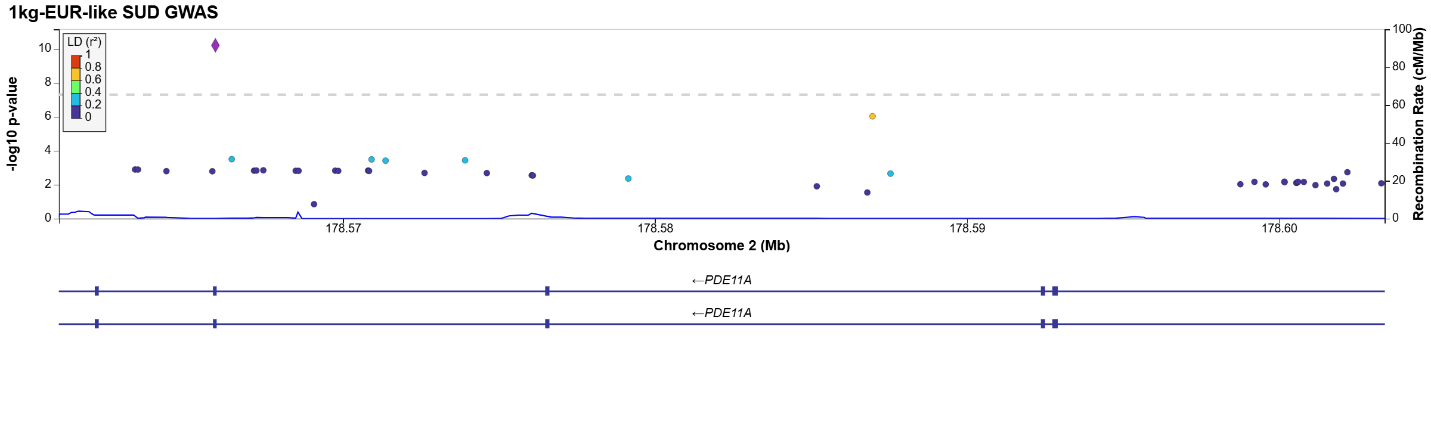


Locus 49:

Locus 50:

Locus 51:

Locus 52:

Locus 53:

Locus 54:

Locus 55:

Locus 56:

Locus 57:

Locus 58:

Locus 59:

Locus 60:

Locus 61:

Locus 62:

Locus 63:

Locus 64:

Locus 65:

Locus 66:

Locus 67:

Locus 68:

Locus 69:

Locus 70:

Locus 71:

Locus 72:

Locus 73:

Locus 74:

Locus 75:

Locus 76:

Locus 77:

Locus 78:

Locus 79:

Locus 80:

Locus 81:

Locus 82:

Locus 83:

Locus 84:

Locus 85:

Locus 86:

Locus 87:

Locus 88:

Locus 89:

Locus 90:

Locus 91:

Locus 92:

Locus 93:

Locus 94:

Locus 95:

Locus 96:

Locus 97:

Locus 98:

Locus 99:

Locus 100:

Locus 101:

Locus 102:

Locus 103:

Locus 104:

Locus 105:

Locus 106:

Locus 107:

Locus 108:

Locus 109:

Locus 110:

Locus 111:

Locus 112:

Locus 113:

Locus 114:

Locus 115:

Locus 116:

Locus 117:

Locus 118:

Locus 119:

Locus 120:

Locus 121:

Locus 122:

Locus 123:

Locus 124:

Locus 125:

Locus 126:

Locus 127:

Locus 128:

Locus 129:

Locus 130:

Locus 131:

Locus 132:

Locus 133:

Locus 134:

Locus 135:

Locus 136:

Locus 137:

Locus 138:

Locus 139:

Locus 140:

Locus 141:

Locus 142:

Locus 143:

Locus 144:

Locus 145:

Locus 146:

Locus 147:

Locus 148:

Locus 149:

Locus 150:

Locus 151:

Locus 152:

Locus 153:

Locus 154:

Locus 155:

Locus 156:

Locus 157:

Locus 158:

Locus 159:

Locus 160:

Locus 161:

Locus 162:

Locus 163:

Locus 164:

Locus 165:

Locus 166:

Locus 167:

Locus 168:

Locus 169:

Locus 170:

Locus 171:

Locus 172:

Locus 173:

Locus 174:

Locus 175:

Locus 176:

Locus 177:

Locus 178:

Locus 179:

Locus 180:

Locus 181:

Locus 182:

Locus 183:

Locus 184:

Locus 185:

Locus 186:

Locus 187:

Locus 188:

Locus 189:

Locus 190:

Locus 191:

Locus 192:

Locus 193:

Locus 194:

Locus 195:

Locus 196:

Locus 197:

Locus 198:

Locus 199:

Locus 200:

Locus 201:

Locus 202:

Locus 203:

Locus 204:

Locus 205:

Locus 206:

Locus 207:

Locus 208:

Locus 209:

Locus 210:

Locus 211:

Locus 212:

Locus 213:

Locus 214:

Locus 215:

Locus 216:

Locus 217:

Locus 218:

Locus 219:

Locus 220:

**Supplemental Figure 2**: Manhattan plots of gene-based analyses. A: 1kg-EUR-like; B: 1kg-AFR-like; C: 1kg-AMR-like; D: meta-analysis of 1kg-AFR-like and 1kg-EUR-like; E: meta-analysis of 1kg-EUR-like and 1kg-AMR-like; F: meta-analysis of 1kg-AFR-like, 1kg-EUR-like, and 1kg-AMR-like.

A: 1kg-EUR-like

B: 1kg-AFR-like

C: 1kg-AMR-like

D: meta-analysis of 1kg-AFR-like and 1kg-EUR-like

E: meta-analysis 1kg-EUR-like and 1kg-AMR-like

F: meta-analysis of 1kg-AFR-like, 1kg-EUR-like, and 1kg-AMR-like
